# Supplementary material for: CC2D1B Coordinates ESCRT-III Activity during the Mitotic Reformation of the Nuclear Envelope
Source: Dev Cell. 2018 Dec 3;47(5):547–563.e6. doi: 10.1016/j.devcel.2018.11.012 (PMC6286407; doi:10.1016/j.devcel.2018.11.012)
Supplement: Document S1. Figures S1–S7 and Table S1 [file mmc1.pdf]

Developmental Cell, Volume *47*

Supplemental Information

CC2D1B Coordinates ESCRT-III Activity during the Mitotic Reformation of the Nuclear Envelope

Leandro N. Ventimiglia, Miguel Angel Cuesta-Geijo, Nicolas Martinelli, Anna Caballe, Pauline Macheboeuf, Nolwenn Miguet, Ian M. Parnham, Yolanda Olmos, Jeremy G. Carlton, Winfried Weissenhorn, and Juan Martin-Serrano

Figure S1

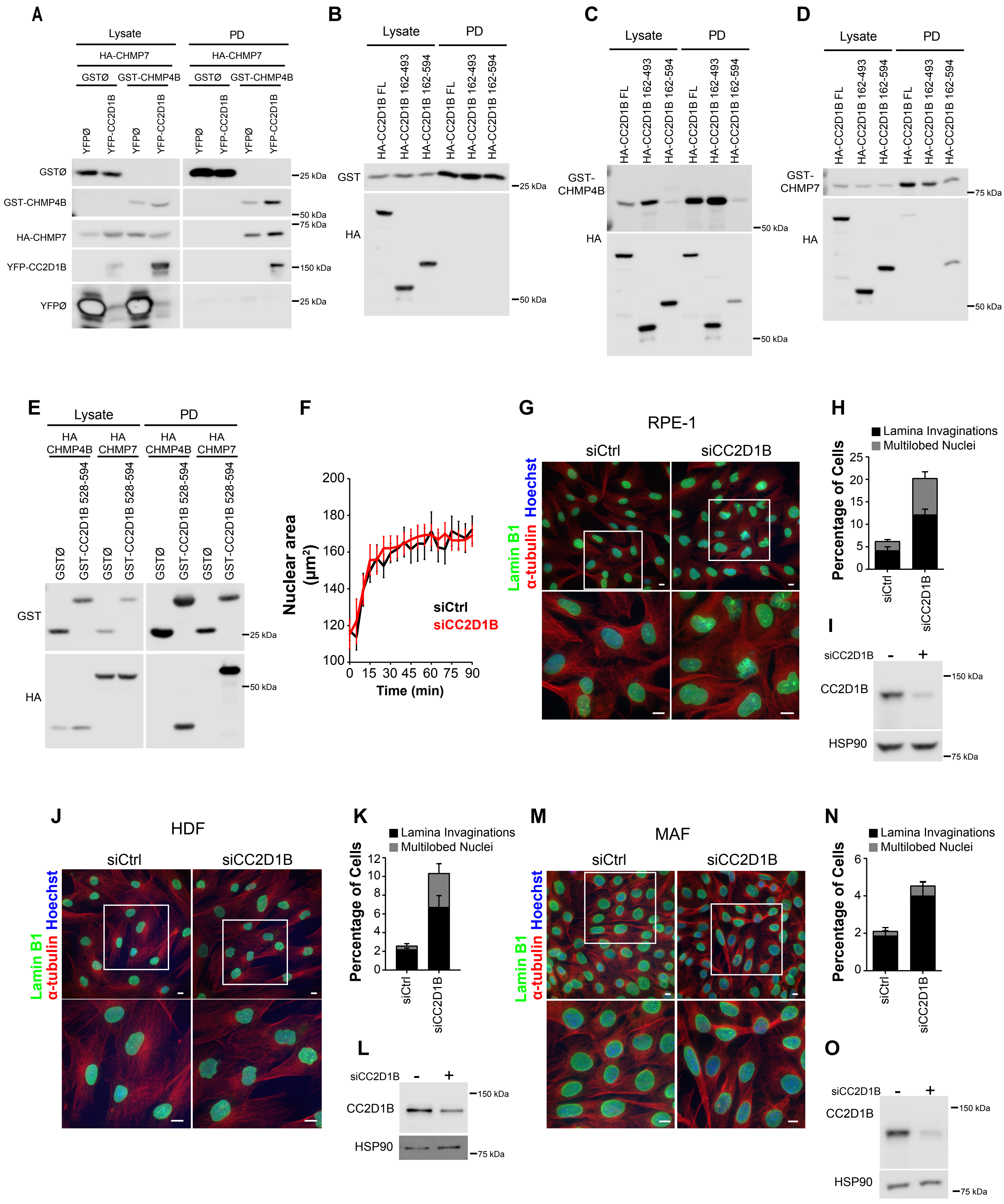
A
Lysate
PD
HA-CHMP7
GSTØ
GST-CHMP4B
YFPØ
YFP-CC2D1B
GSTØ
GST-CHMP4B
HA-CHMP7
YFP-CC2D1B
YFPØ
25 kDa
50 kDa
75 kDa
150 kDa
B
HA-CC2D1B FL
HA-CC2D1B 162-493
HA-CC2D1B 162-594
GST
HA
C
GST-CHMP4B
D
GST-CHMP7
E
HA CHMP4B
HA CHMP7
GST-CC2D1B 528-594
F
Nuclear area (µm²)
Time (min)
siCtrl
siCC2D1B
G
RPE-1
Lamin B1
α-tubulin
Hoechst
H
Lamina Invaginations
Multilobed Nuclei
Percentage of Cells
I
CC2D1B
HSP90
J
HDF
K
L
M
MAF
N
O

Figure S2

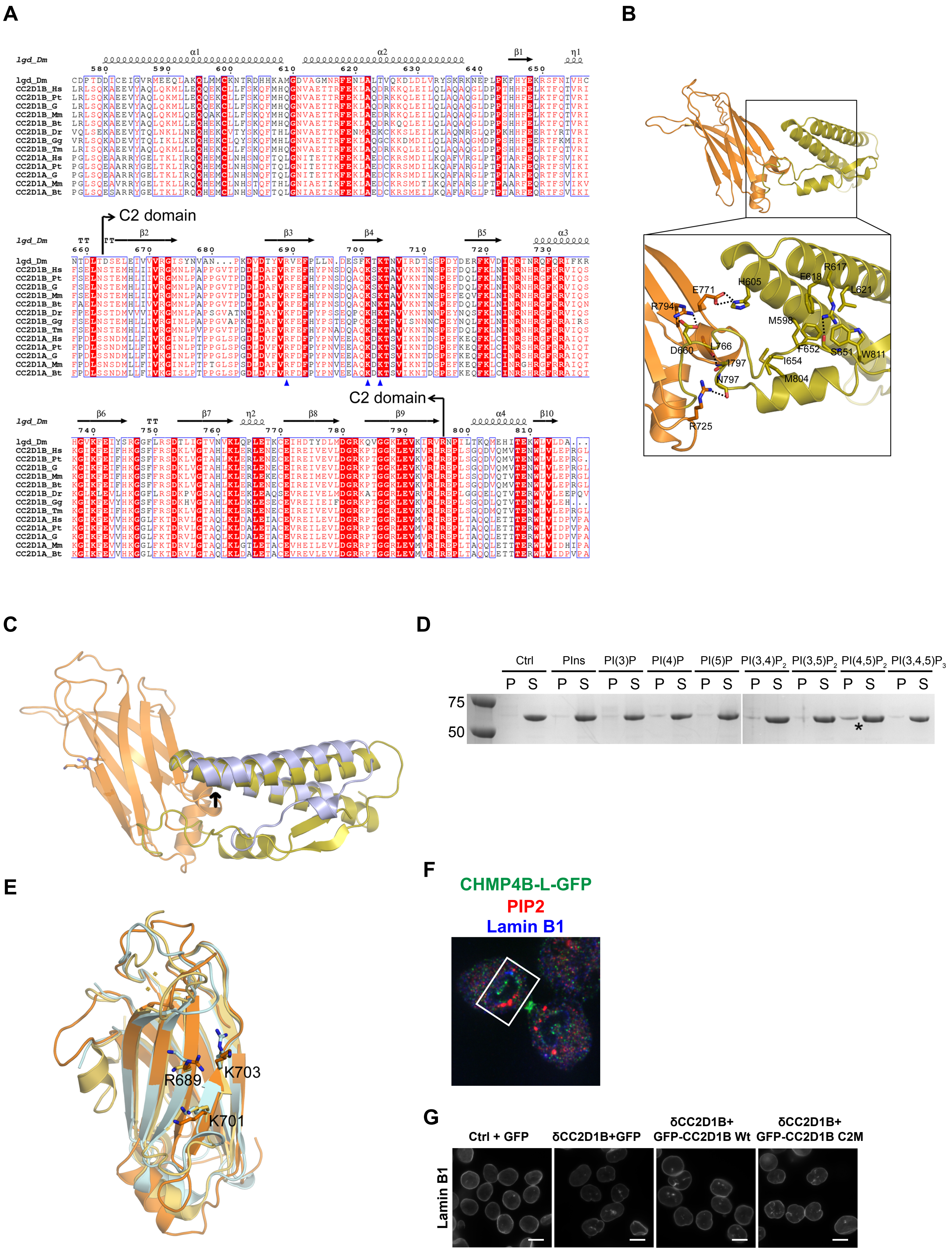
A
B
C
D
E
F
G
C2 domain
C2 domain
lgd_Dm
CC2D1B_Hs
CC2D1B_Pt
CC2D1B_G
CC2D1B_Mm
CC2D1B_Bt
CC2D1B_Dr
CC2D1B_Gg
CC2D1B_Tm
CC2D1A_Hs
CC2D1A_Pt
CC2D1A_G
CC2D1A_Mm
CC2D1A_Bt
E771
H605
R617
F618
L621
R794
M598
L766
D660
I797
F652
S651
W811
I654
N797
M804
R725
Ctrl
PIns
PI(3)P
PI(4)P
PI(5)P
PI(3,4)P2
PI(3,5)P2
PI(4,5)P2
PI(3,4,5)P3
P S
75
50
*
R689
K703
K701
CHMP4B-L-GFP
PIP2
Lamin B1
Ctrl + GFP
δCC2D1B+GFP
δCC2D1B+
GFP-CC2D1B Wt
δCC2D1B+
GFP-CC2D1B C2M
Lamin B1

Figure S3

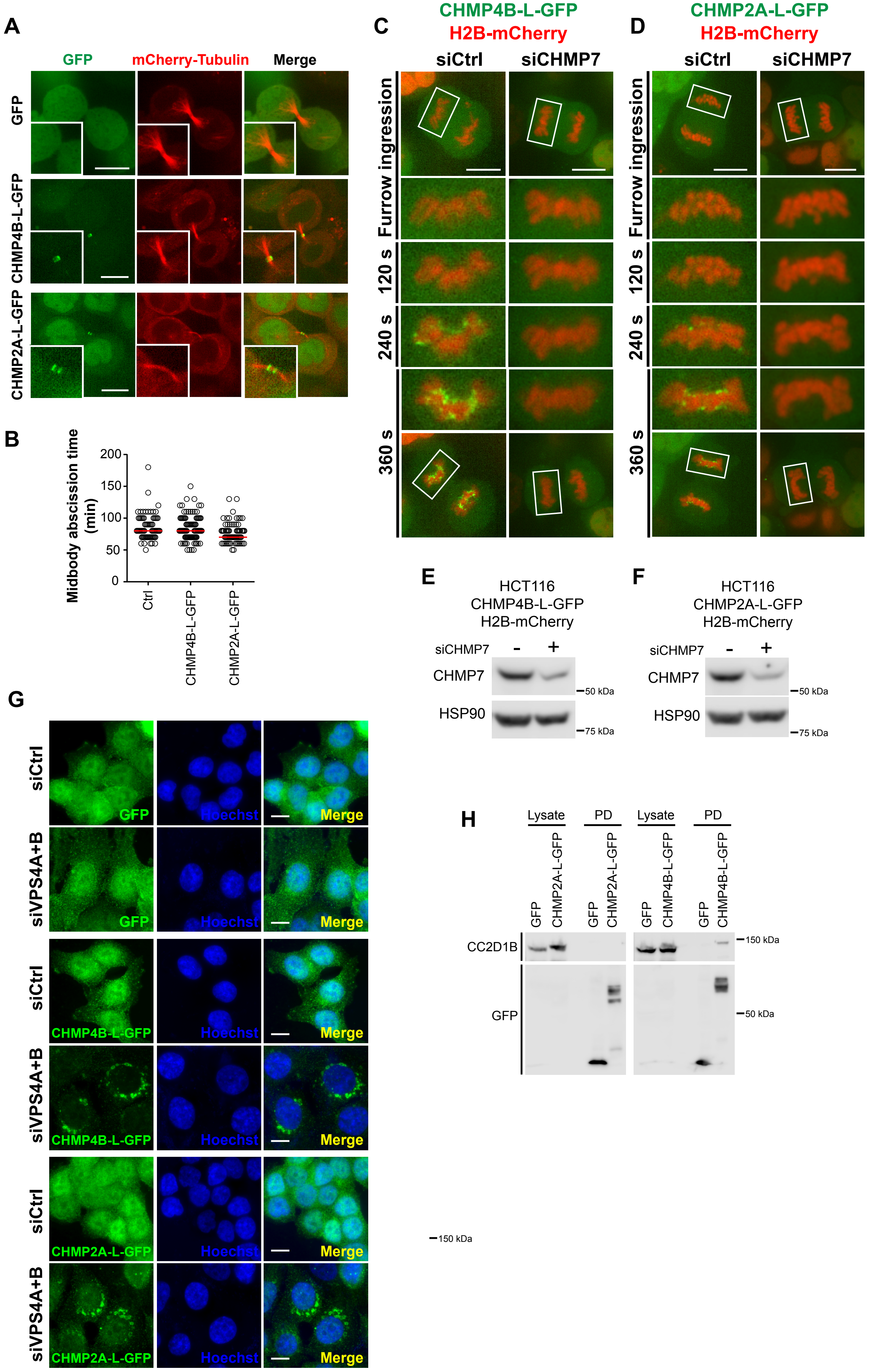
A
GFP
mCherry-Tubulin
Merge
GFP
CHMP4B-L-GFP
CHMP2A-L-GFP
B
Midbody abscission time (min)
200
150
100
50
0
Ctrl
CHMP4B-L-GFP
CHMP2A-L-GFP
C
CHMP4B-L-GFP
H2B-mCherry
siCtrl
siCHMP7
Furrow ingression
120 s
240 s
360 s
D
CHMP2A-L-GFP
H2B-mCherry
siCtrl
siCHMP7
Furrow ingression
120 s
240 s
360 s
E
HCT116
CHMP4B-L-GFP
H2B-mCherry
siCHMP7 - +
CHMP7
HSP90
50 kDa
75 kDa
F
HCT116
CHMP2A-L-GFP
H2B-mCherry
siCHMP7 - +
CHMP7
HSP90
50 kDa
75 kDa
G
siCtrl
siVPS4A+B
GFP
Hoechst
Merge
CHMP4B-L-GFP
CHMP2A-L-GFP
H
Lysate
PD
GFP
CHMP2A-L-GFP
CHMP4B-L-GFP
CC2D1B
GFP
150 kDa
50 kDa
150 kDa

Figure S4

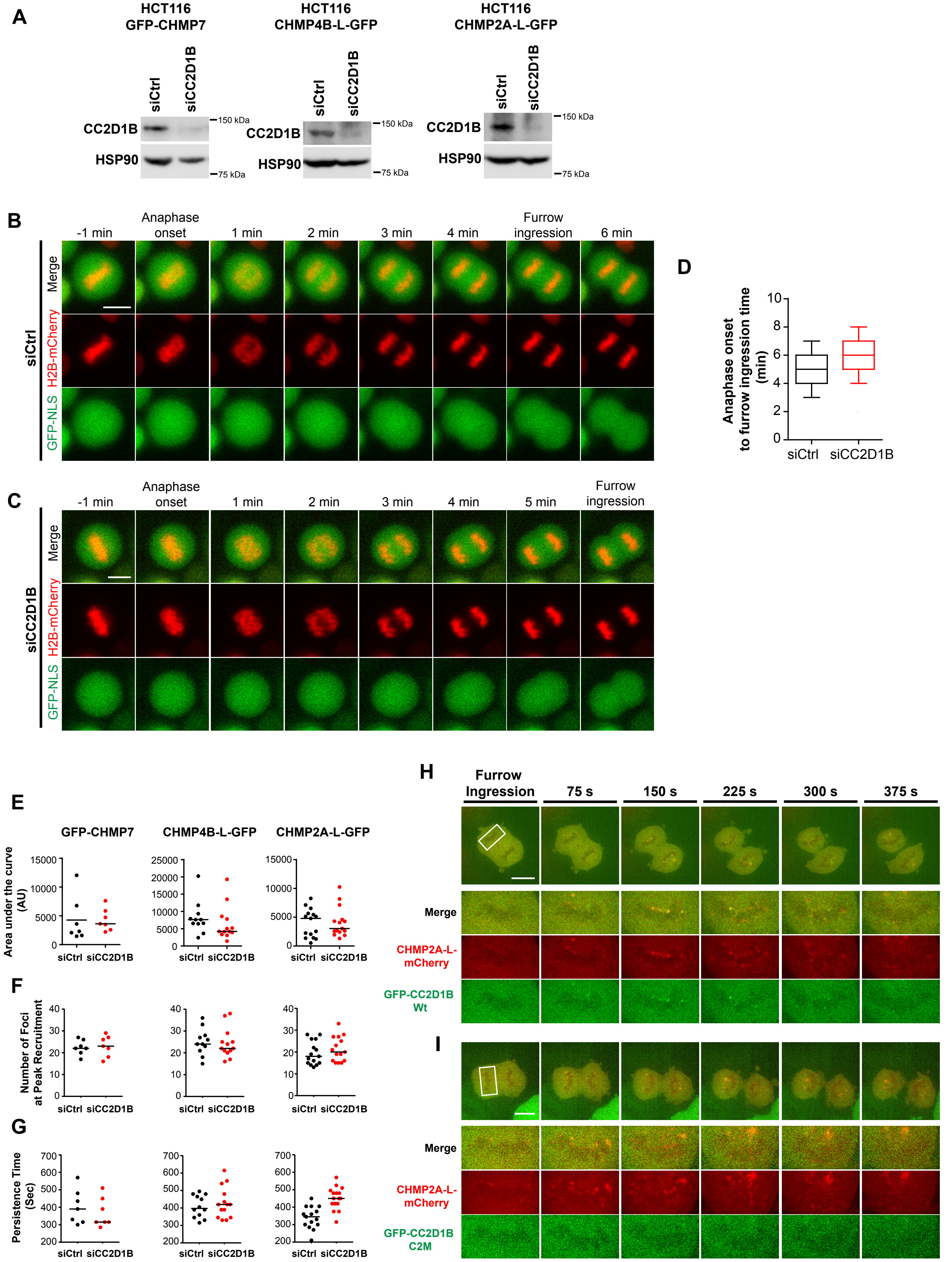

A
HCT116 GFP-CHMP7
HCT116 CHMP4B-L-GFP
HCT116 CHMP2A-L-GFP
siCtrl
siCC2D1B
CC2D1B
HSP90
150 kDa
75 kDa
B
-1 min
Anaphase onset
1 min
2 min
3 min
4 min
Furrow ingression
6 min
siCtrl
Merge
H2B-mCherry
GFP-NLS
C
5 min
siCC2D1B
D
Anaphase onset to furrow ingression time (min)
0
2
4
6
8
10
E
GFP-CHMP7
CHMP4B-L-GFP
CHMP2A-L-GFP
Area under the curve (AU)
F
Number of Foci at Peak Recruitment
G
Persistence Time (Sec)
H
Furrow Ingression
75 s
150 s
225 s
300 s
375 s
CHMP2A-L-mCherry
GFP-CC2D1B Wt
I
GFP-CC2D1B C2M

Figure S5

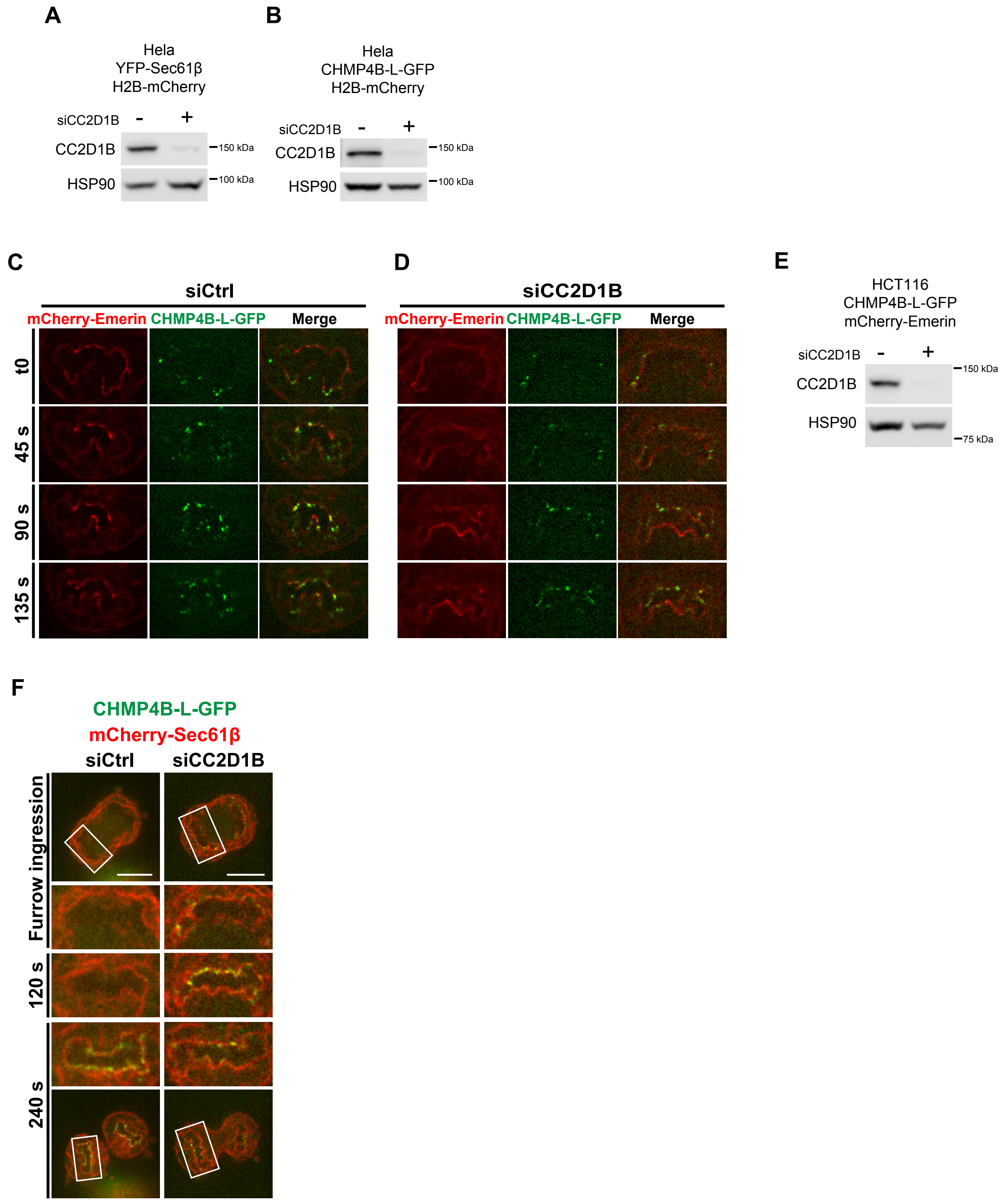

A
Hela
YFP-Sec61β
H2B-mCherry
siCC2D1B - +
CC2D1B
150 kDa
HSP90
100 kDa
B
Hela
CHMP4B-L-GFP
H2B-mCherry
siCC2D1B - +
CC2D1B
150 kDa
HSP90
100 kDa
C
siCtrl
mCherry-Emerin
CHMP4B-L-GFP
Merge
t0
45 s
90 s
135 s
D
siCC2D1B
mCherry-Emerin
CHMP4B-L-GFP
Merge
E
HCT116
CHMP4B-L-GFP
mCherry-Emerin
siCC2D1B - +
CC2D1B
150 kDa
HSP90
75 kDa
F
CHMP4B-L-GFP
mCherry-Sec61β
siCtrl
siCC2D1B
Furrow ingression
120 s
240 s

Figure S6

A

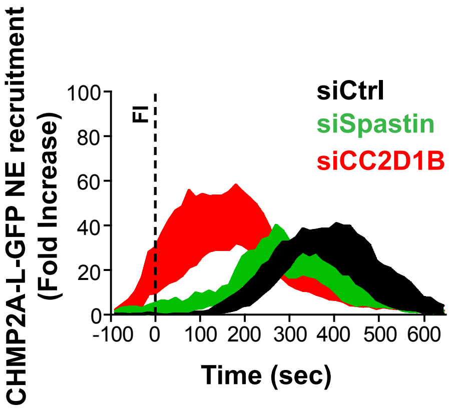
CHMP2A-L-GFP NE recruitment
(Fold Increase)
100
80
60
40
20
0
FI
-100 0 100 200 300 400 500 600
Time (sec)
siCtrl
siSpastin
siCC2D1B

B

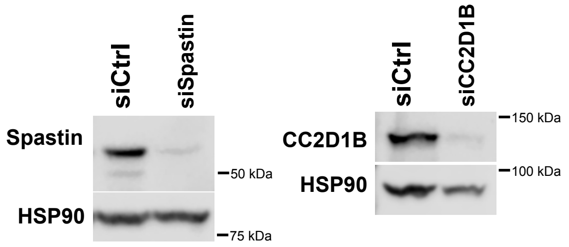
siCtrl
siSpastin
Spastin
HSP90
50 kDa
75 kDa
siCtrl
siCC2D1B
CC2D1B
HSP90
150 kDa
100 kDa

Figure S7

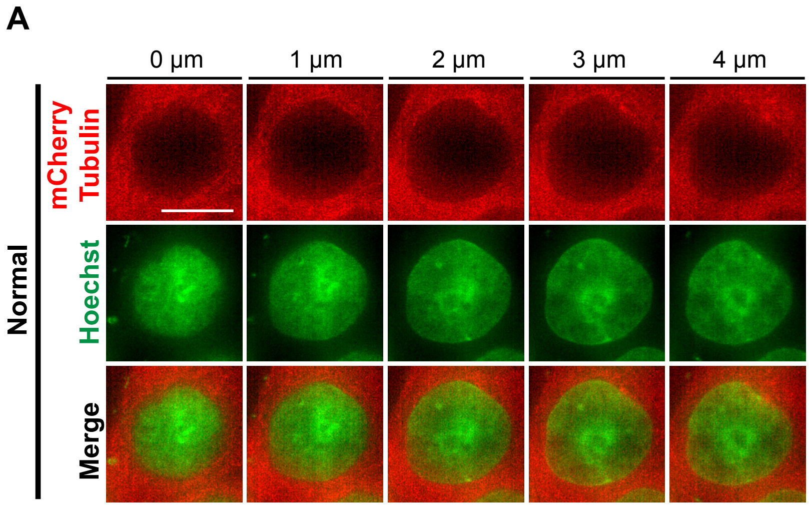

Table S1. Related to Figure 3. Data collection and refinement statistics

	LgD SAD	LgD native (PDB code)
**Data collection**		
Space group	P1 21 1	P1 21 1
Cell dimensions		
a, *b*, *c* (Å)	87.35, 53.59, 97.85	87.81, 54.22, 98.19
α, β, γ (°)	90, 99.49, 90	90, 99.49, 90
Wavelength (Å)	0.9795	0.9750
Resolution (Å)	96.5 2.9 (3.06-2.9)[a]	9-.55-2.46 (2.59-2.46)
R_{merge}	8.5 (34.5)	7.2 (36.3)
$I/\sigma(I)$	10.1 (3.8)	12.0 (3.6)
$CC_{1/2}$	99.3 (89.2)	99.5 (88.0)
Completeness (%)	99.4 (99.5)	99.4 (99.2)
Redundancy	3.9 (3.9)	3.5 (3.5)
**Refinement**		
Resolution (Å)		59.59-2.46 (2.53-2.46)
No. reflections		32921 (3242)
R_{work} / R_{free}		0.2067/0.2459 (0.2846/0.3533)
No. atoms		4158
Protein		3952
Water		164
Ligands		42
B factors		
Protein		47.96
Water		44.78
Ligands		77.20
R.m.s. deviations		
Bond lengths (Å)		0.010
Bond angles (°)		1.23

[a] Values in parentheses are for highest-resolution shell.

Supplemental information

Figure S1. Related to Figure 1.

(A) GST pulldown experiments of 293T cells transiently co-expressing GST-CHMP4B, HA-CHMP7 and YFP-CC2D1B.

(B) GST pulldown experiments of 293T cells transiently co-expressing GST together with HA-CC2D1B full length, HA-CC2D1B (residues 162-493) or HA-CC2D1B (residues 162-594).

(C) GST pulldown experiments of 293T cells transiently co-expressing GST-CHMP4B together with HA-CC2D1B full length, HA-CC2D1B (residues 162-493) or HA-CC2D1B (residues 162-594).

(D) GST pulldown experiments of 293T cells transiently co-expressing GST-CHMP7 together with HA-CC2D1B full length, HA-CC2D1B (residues 162-493) or HA-CC2D1B (residues 162-594).

(E) GST pulldown experiments of 293T cells transiently co-expressing GST-CC2D1B (residues 528-594) together with HA-CHMP7 or HA-CHMP4B.

(F) Representation the nuclear expansion rate in HCT116 cells stably co-expressing GFP-NLS and H2B-mCherry. Mean±SEM; siControl n=15; siCC2D1B n=21, p=0.5941. Significance compared with the control was calculated at 90 minutes using a two-tailed unpaired t-test.

(G-I) Analysis of nuclear morphology in siRNA transfected RPE-1 cells. (G) Representative images corresponding to the quantifications shown in H. Scale bars 10µm. (H) Percentage of cells showing an aberrant nuclear morphology. Mean±SEM; siControl n=1192; siCC2D1B n=924, p=0.0057. Significance of NE invaginations compared to the control was calculated using a two-tailed unpaired t-test. (I) Representative WB corresponding to cells shown in G.

(J-L) Analysis of nuclear morphology in siRNA transfected HDF cells. (J) Representative images corresponding to the quantifications shown in K. Scale bars 10µm. (K) Percentage of cells showing an aberrant nuclear morphology. Mean±SEM; siControl n=731; siCC2D1B n=736, p=0.0216. Significance of NE

invaginations compared to the control was calculated using a two-tailed unpaired t-test. (L) Representative WB corresponding to cells shown in J.

(M-O) Analysis of nuclear morphology in siRNA transfected MEF cells. (M) Representative images corresponding to the quantifications shown in N. Scale bars 10µm. (N) Percentage of cells showing an aberrant nuclear morphology. Mean±SEM; siControl n=1589; siCC2D1B n=1523, p=0.0387. Significance of NE invaginations compared to the control was calculated using a two-tailed unpaired t-test. (O) Representative WB corresponding to cells shown in M.

Figure S2. Related to Figure 3.

(A) Sequence alignment of the C-terminal domains of the Lgd/CC2D1 family of proteins. Secondary structure elements are shown based on the Lgd (residues 575-816) crystal structure. The sequence corresponding to the C2 domain is indicated as well as the conserved residues that may coordinate PIns(4,5)P_2 (blue triangles). The following sequences are shown: accession code AAF53069 lgd drosophila melanogaster (Dm) isoform A; XP_005270647 Homo sapiens (Hs) 1B isoform X1; JAA36276 CC2D1B Pan troglodytes (Pt); XP_004025849 gorilla gorilla (G) 1B; XP_006503215 mus musculus (Mm) 1B isoform x1; XP_002686449 Bos taurus (Bt) 1B ; XP_005171255 Danio rerio (Dr) 1B X1 ; XP_001235393 Gallus gallus (Gg) 1B ; XP_004371788 CC2D1B Trichechus manatus latirostris (Tm); XP_005260029 Homo sapiens (Hs) 1A isoform X1; XP_524131 Pan troglodytes (Pt) 1A; XP_004060195 gorilla gorilla (G) 1A; XP_006530873 mus musculus (Mm)1A isoform X1; XP_005208662 Bos taurus (Bt) 1A isoform x1.

(B) Close up of the interactions stabilizing the helical domain and its orientation towards the C2 domain; the helical hairpin interacts via a hydrophobic core and hydrogen bonds with the coil region connecting to the C2 domain and its orientation is stabilized by salt bridges and hydrogen bonds producing a rigid structure. Polar interactions are indicated by dashed lines.

(C) Cα superposition of the Lgd helical hairpin and the DM14-3 domain (pdb code 5vo5) shows high structural similarity of the C-terminal helical domain

(yellow) and the DM14-3 domain (light blue). Shrub/CHMP4 binds perpendicular to the tip of the helical hairpin of DM14-3 (indicated by the arrow).

(D) Analysis of Lgd (residues 358-816) binding to phosphatidylinositol by pull-down; Ctrl, control beads; P, pellet; S, supernatant. A significant pull down is observed with PIns(4,5)P_2 (*).

(E) Ribbon diagram of the superpositioning of the Cα atoms of Lgd (orange) and the C2 domains from protein kinase C (yellow, pdb entry 3GPE) and synaptotagmin C2A (polycyan, pdb entry 1RSY); the residues of the basic cluster implicated in PIns(4,5)P_2 binding are indicated and correspond to CC2D1B residues R721, K734, K736.

(F) HCT116 dividing cell stably expressing CHMP4B-L-GFP and stained with anti-GFP, -PIP2 and Lamin B1 antibodies. The nuclear inset is shown in Figure 3G.

(G) Representative images of the HCT116 cells used for quantification shown in Fig 3I. Scale bars 10µm.

Figure S3. Related to Figure 4.

Functional characterization of HCT116 cells stably expressing CHMP4B-L-GFP or CHMP2A-L-GFP.

(A) Representative images of dividing living cells stably co-expressing CHMP4B-L-GFP or CHMP2A-L-GFP along with mCherry-Tubulin. Scale bars 10µm.

(B) Midbody abscission time in cells stably co-expressing CHMP4B-L-GFP or CHMP2A-L-GFP along with mCherry-Tubulin. Bars indicate Median. Control n=74; CHMP4B-L-GFP n=97, p=0.5748; CHMP2A-L-GFP n=75, p=0.0015. Significance compared to the control was calculated using a two-tailed unpaired t-test.

(C-F) Time-lapse analysis of HCT116 cells stably co-expressing CHMP4B-L-GFP (C) or CHMP2A-L-GFP (D) along with H2B-mCherry. Cells were transfected with control or CHMP7 siRNAs. T0 was set at the beginning of furrow ingression. (E-F) Representative WB corresponding to cells shown in C and D. Scale bars 10µm in C and D.

(G) Representative images of HCT116 cells stably expressing CHMP4B-L-GFP or CHMP2A-L-GFP transfected with control or VPS4A and VPS4B siRNAs. Cells were stained with an anti-GFP antibody. Scale bars 10µm.

(H) Representative WB corresponding to a GFP immunoprecipitation experiment of HCT116 cells stably expressing CHMP4B-L-GFP or CHMP2A-L-GFP.

See also Movies 6 and 7.

Figure S4. Related to Figure 4.

(A) Representative WB corresponding to cells shown in Fig 4 A-C.

(B-D) Anaphase onset to furrow ingression time quantification of HCT116 cells stably co-expressing GFP-NLS with H2B-mCherry. Representative time lapse images corresponding to cells transfected with control (B) or CC2D1B (C) siRNAs. (D) Anaphase onset to furrow ingression time quantification. Bar indicates Median. siCtrl n = 348; siCC2D1B n = 362, $p<0.0001$. Significance compared to the control was calculated using a two-tailed unpaired t-test. Scale bars 10µm in B and C.

(E) Area under the curves corresponding to the total amount of GFP-CHMP7, CHMP4B-L-GFP or CHMP2A-L-GFP fluorescence recruited to the reforming NE. Cells were transfected with control or CC2D1B siRNA. Bar indicates Median. GFP-CHMP7 $p=0.9836$; CHMP4B-L-GFP $p=0.3858$; CHMP2A-L-GFP $p=0.8959$. Significance compared to the control was calculated using a two-tailed unpaired t-test.

(F) Number of GFP-CHMP7, CHMP4B-L-GFP or CHMP2A-L-GFP foci on the NE at peak recruitment. Cells were transfected with control or CC2D1B siRNA. Bar indicates Median. GFP-CHMP7 $p=0.8006$; CHMP4B-L-GFP $p=0.8180$; CHMP2A-L-GFP $p=0.3793$. Significance compared to the control was calculated using a two-tailed unpaired t-test.

(G) Persistence time of GFP-CHMP7, CHMP4B-L-GFP or CHMP2A-L-GFP fluorescence on the NE. Cells were transfected with control or CC2D1B siRNA.

Bar indicates Median. GFP-CHMP7 p=0.4986; CHMP4B-L-GFP p=0.5039; CHMP2A-L-GFP p=<0.0001. Significance compared to the control was calculated using a two-tailed unpaired t-test.

(H-I) Representative time lapse analysis corresponding to HCT116$^{\delta CC2D1B}$ cells co-expressing CHMP2A-L-mCherry and GFP-CC2D1B Wt (H) or C2M (I). Scale bars 10µm.

See also Movie 11 and 12.

Figure S5. Related to Figure 5.

(A-B) Representative WB corresponding to cells shown in Figures 5A and 5B.

(C-E) Super resolution time-lapse analysis of HCT116 cells stably co-expressing CHMP4B-L-GFP and mCherry-Emerin corresponding to the images shown in Figure 5D. Cells were transfected with control (C) or CC2D1B (D) siRNAs. (E) Representative WB corresponding to cells shown in Figure 5D and Figure S5C-S5D.

(F) Time-lapse analysis of HCT116 cells stably co-expressing CHMP4B-L-GFP and mCherry-Sec61β. Cells were transfected with control or CC2D1B siRNAs. Scale bars 10µm.

See also Movies 13.

Figure S6. Related to Figure 6.

(A) Quantification of CHMP2A-L-GFP recruitment to the reforming NE in HCT116 cells stably co-expressing CHMP2A-L-GFP with mCherry-Tubulin and transfected with control, Spastin or CC2D1B siRNAs. T0 was set at the beginning of furrow ingression (FI).

(B) Representative WB corresponding to cells shown in Figure 6E**.**

Figure S7. Related to Figure 7.

(A) Z-stack series corresponding to a HCT116 cell stably co-expressing mCherry-Tubulin with CHMP2A-L-GFP, transfected with control siRNA and incubated with Hoechst 33258 30’ before imaging to stain DNA. The signal corresponding to Hoechst 33258 is shown in green. The signal corresponding to CHMP2A-L-GFP is not shown. This cell was selected as an example of a nucleus with normal rounded morphology. Scale bar 10µm.

See also Movie 16.

Table S1. Related to Figure 3.

Data collection and refinement statistics of Lgd (residues 575-816) solved structure.
