# Supplementary material for: CC2D1B Coordinates ESCRT-III Activity during the Mitotic Reformation of the Nuclear Envelope
Source: Dev Cell. 2018 Dec 3;47(5):547–563.e6. doi: 10.1016/j.devcel.2018.11.012 (PMC6286407; doi:10.1016/j.devcel.2018.11.012)

# Data S1

## Fig 1B Left

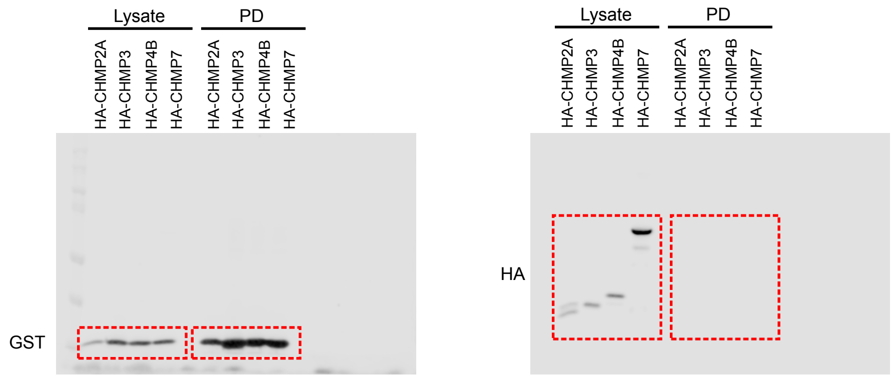


## Fig 1B Right

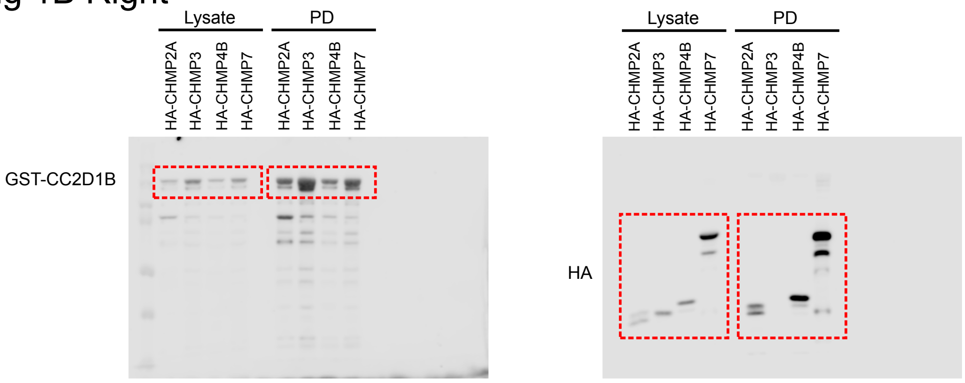


## Fig 1C

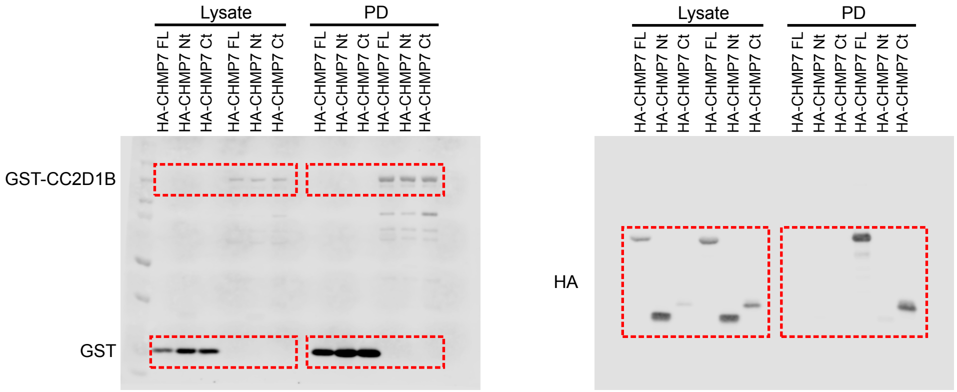


## Fig 1E Left

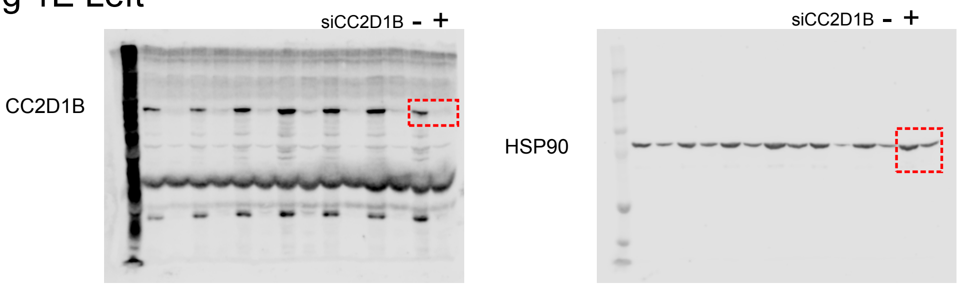


## Fig 1E Right

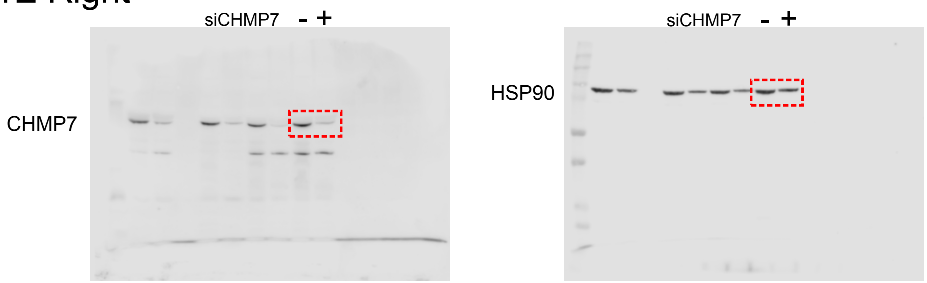

# Figure S1I

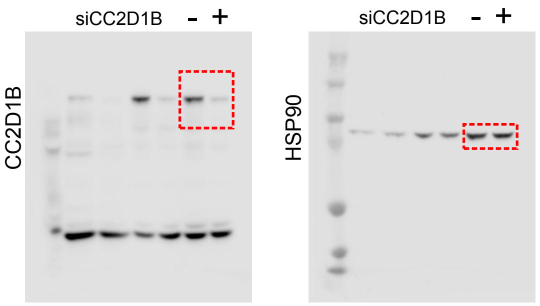


# Figure S1L

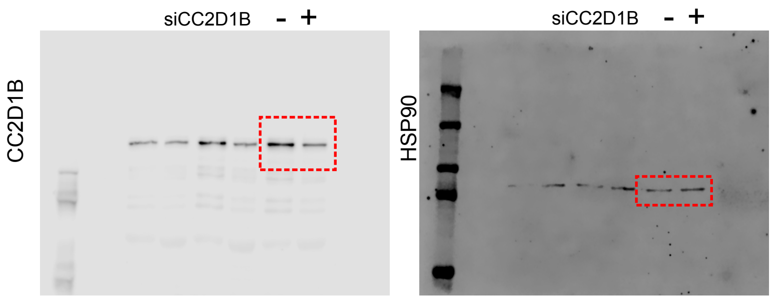


# Figure S1O

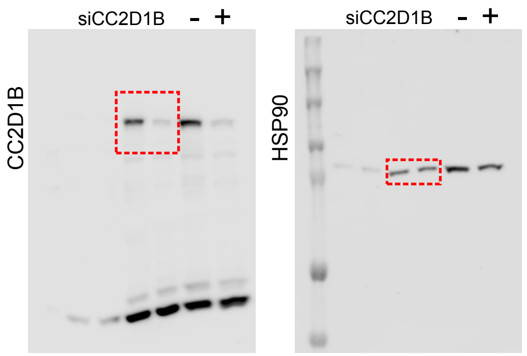

## Fig 2A

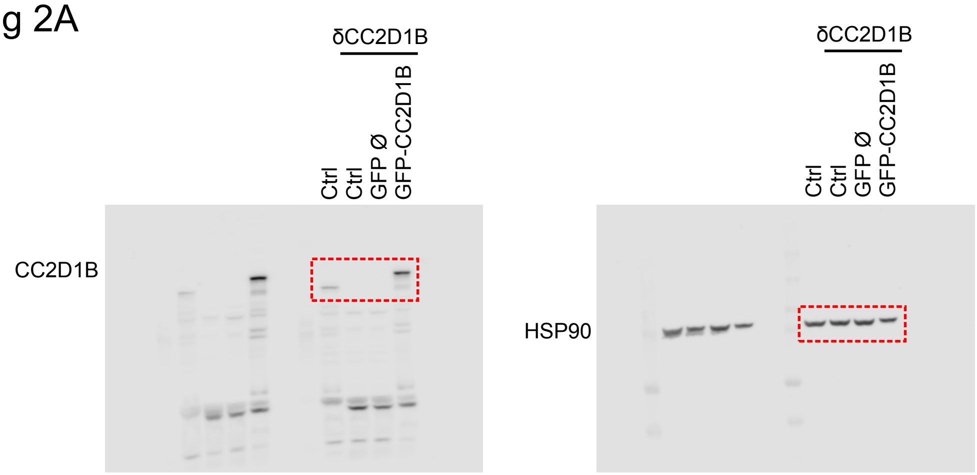


## Fig 2F

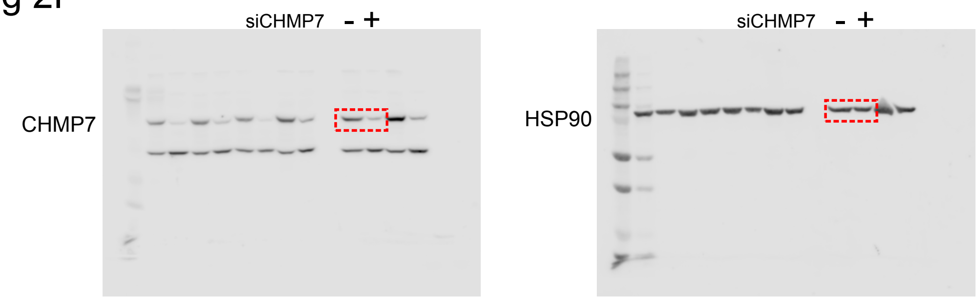


## Fig 3E

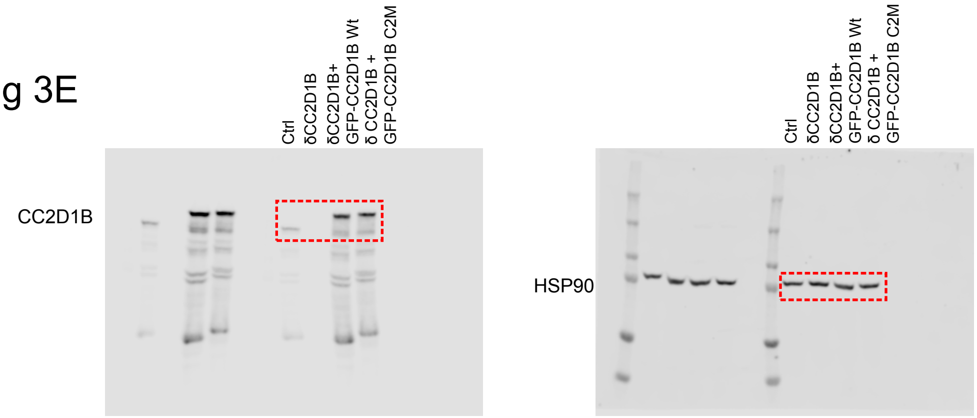

Fig 6C Top

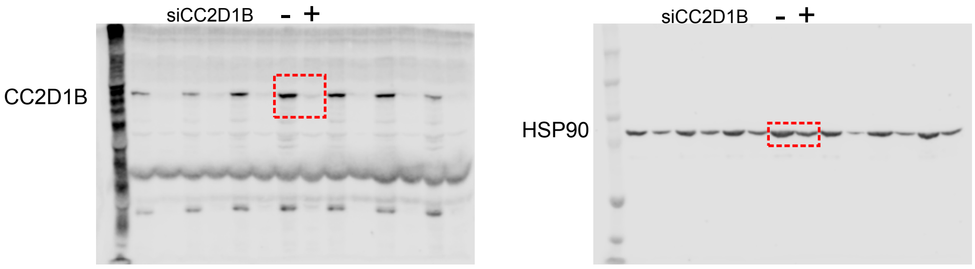


Fig 6C Bottom

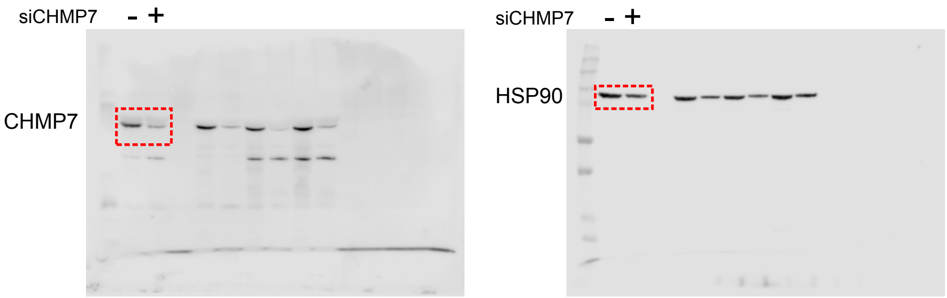

# Figure S1A

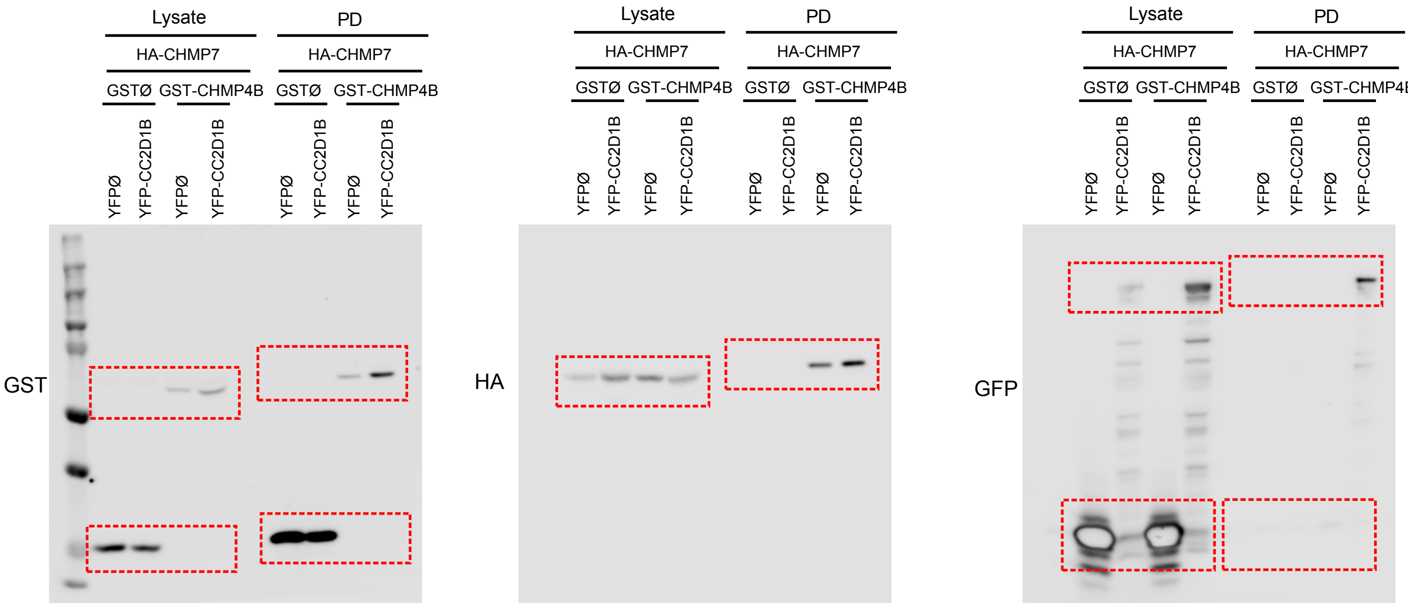


# Figure S1B

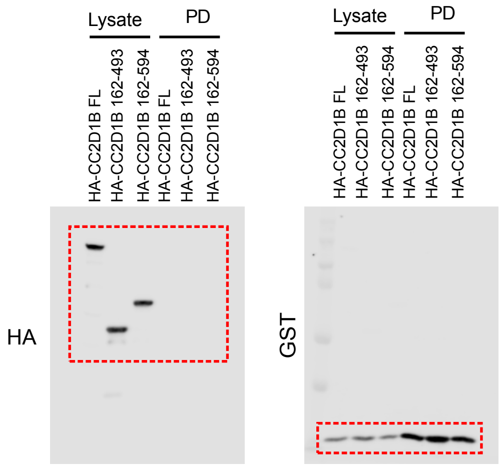


# Figure S1C

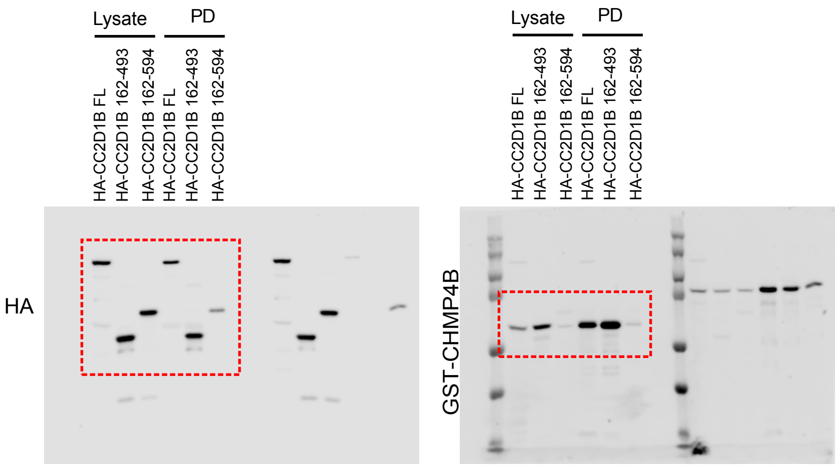


# Figure S1E

# Figure S1D

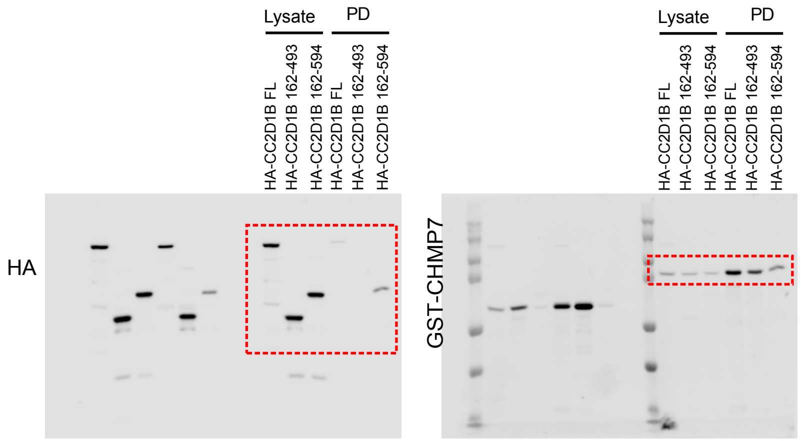


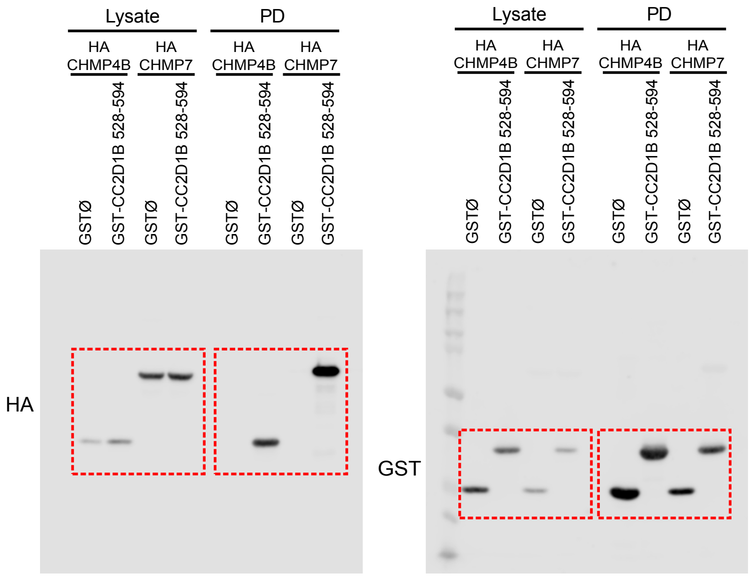

# Figure S1I

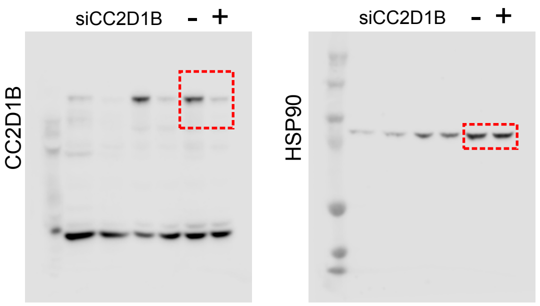


# Figure S1L

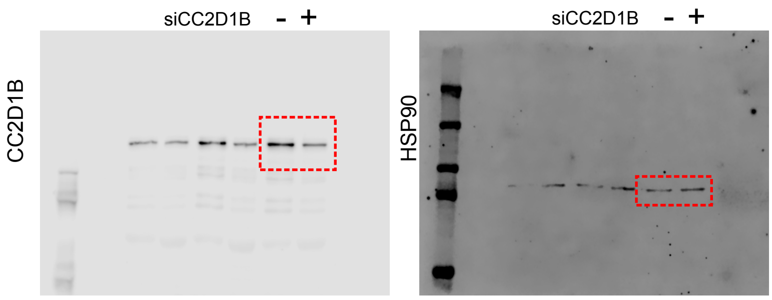


# Figure S1O

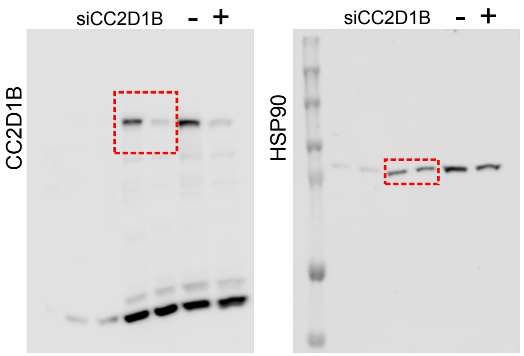

# Figure S2D

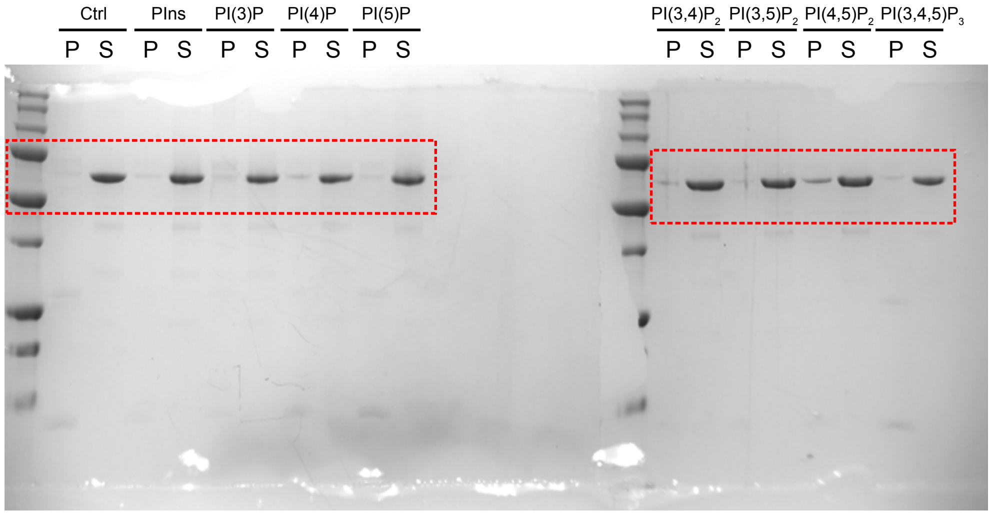
Ctrl
PIns
PI(3)P
PI(4)P
PI(5)P
PI(3,4)P2
PI(3,5)P2
PI(4,5)P2
PI(3,4,5)P3
P S P S P S P S P S
P S P S P S P S

# Figure S3E

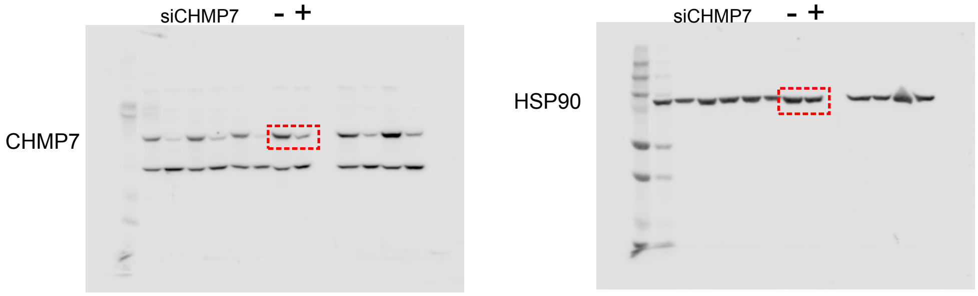


# Figure S3F

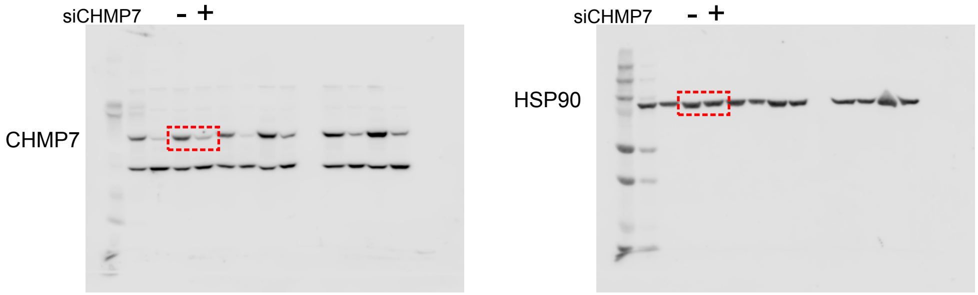


# Figure S3H

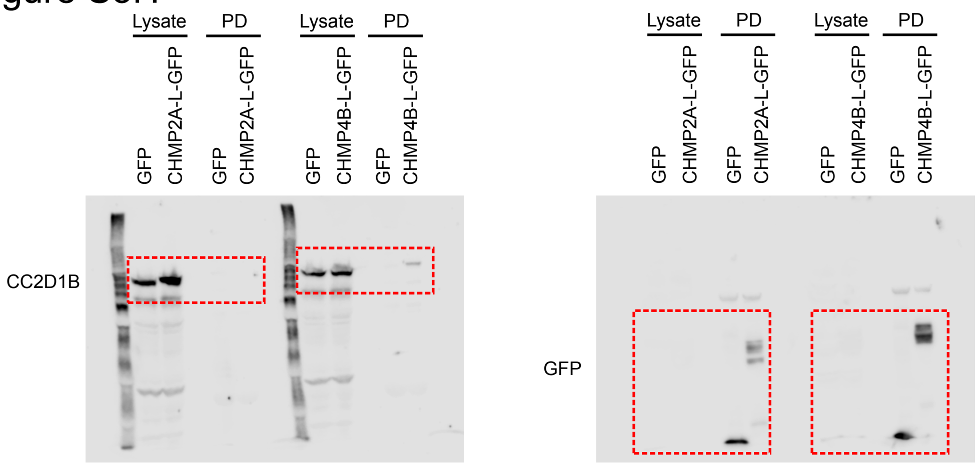

# Figure S4A Left

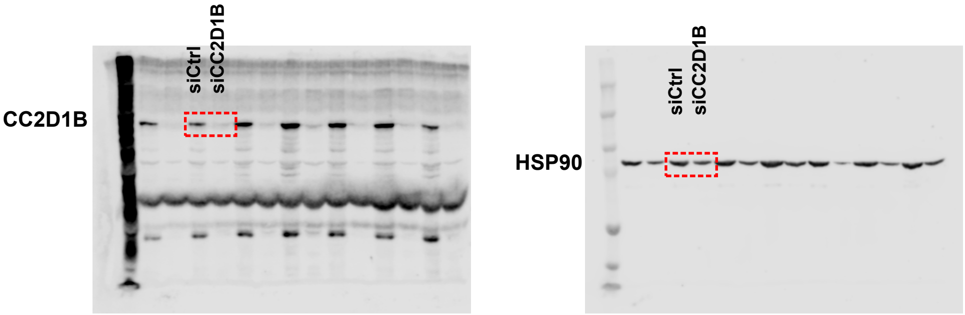


# Figure S4A Middle

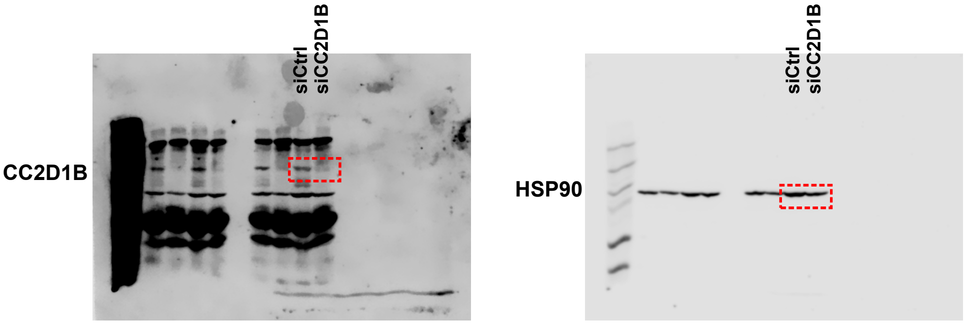


# Figure S4A Right

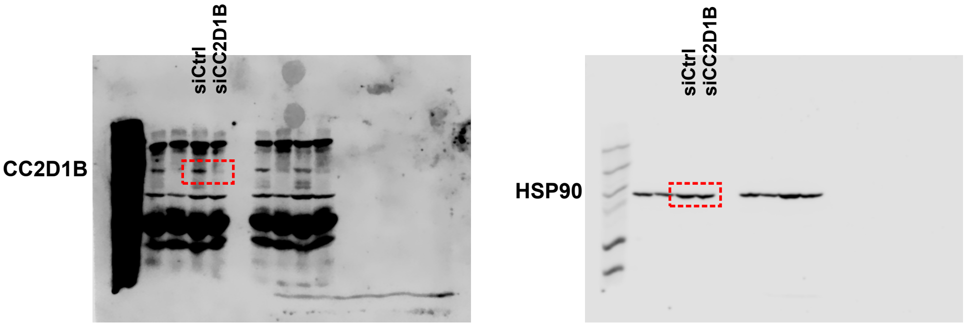

## Fig S5A

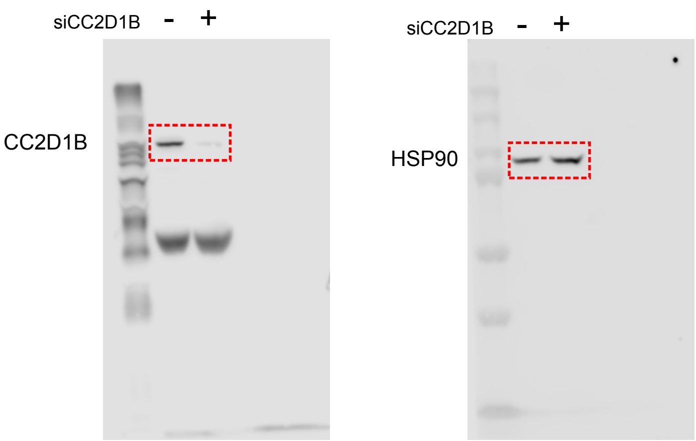


## Fig S5B

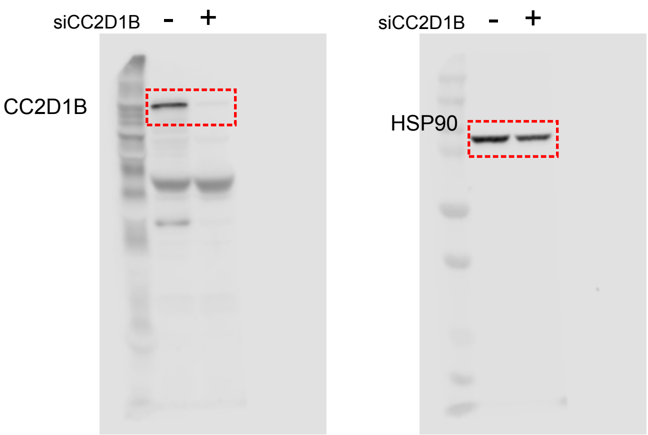


## Figure S5E

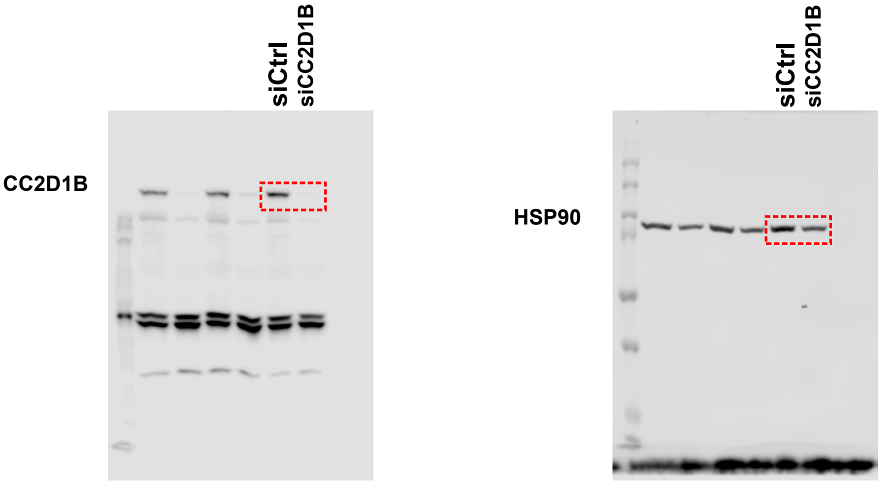

## Figure S6B Left

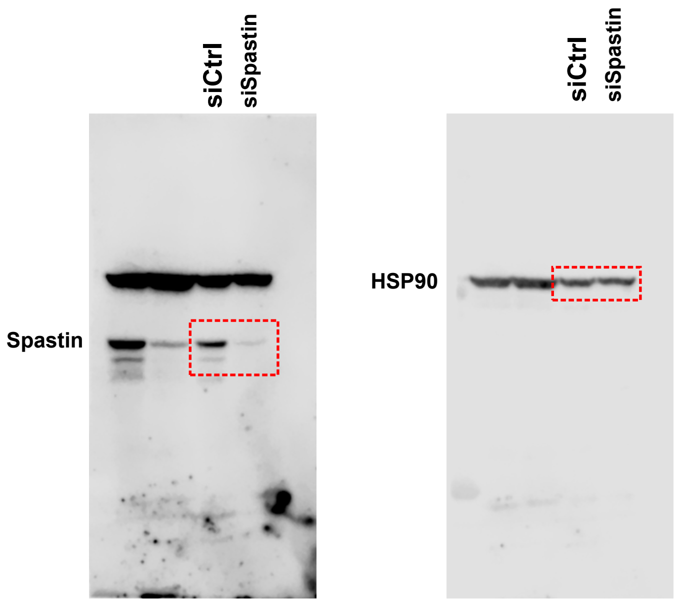


## Figure S6B Right

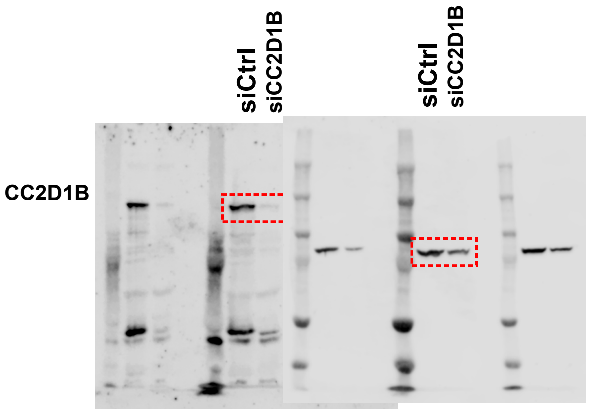

Supplement: Data S1. Unprocessed Images of Immunoblots and Coomasie Blue Staining, Related to the Immunoblotting Section of the Method Details — Unprocessed images of immunoblots and Coomasie Blue staining shown in Fig 1B Left, Fig 1B Right, Fig 1C, Fig 1E Left, Fig 1E Right, Fig 2A, Fig 2F, Fig 3E, Fig 6C Top, Fig 6C Bottom, Fig S1A, Fig S1B, Fig S1C, Fig S1D, Fig S1E, Fig S1I, Fig S1L, Fig S1O, Fig S2D, Fig S3E, Fig S3F, Fig S3H, Fig S4A Left, Fig S4A Middle, Fig S4A Right, Fig S5A, Fig S5B Left, Fig S5B Right, Fig S5E, Fig S6B Left and Fig S6B Right. Red rectangles indicate cropped images shown in figures. [file mmc2.pdf]
