# Supplementary material for: CC2D1B Coordinates ESCRT-III Activity during the Mitotic Reformation of the Nuclear Envelope
Source: Dev Cell. 2018 Dec 3;47(5):547–563.e6. doi: 10.1016/j.devcel.2018.11.012 (PMC6286407; doi:10.1016/j.devcel.2018.11.012)
Supplement: Document S2. Article plus Supplemental Information [file mmc19.pdf]

Article

Developmental Cell

CC2D1B Coordinates ESCRT-III Activity during the Mitotic Reformation of the Nuclear Envelope

Graphical Abstract

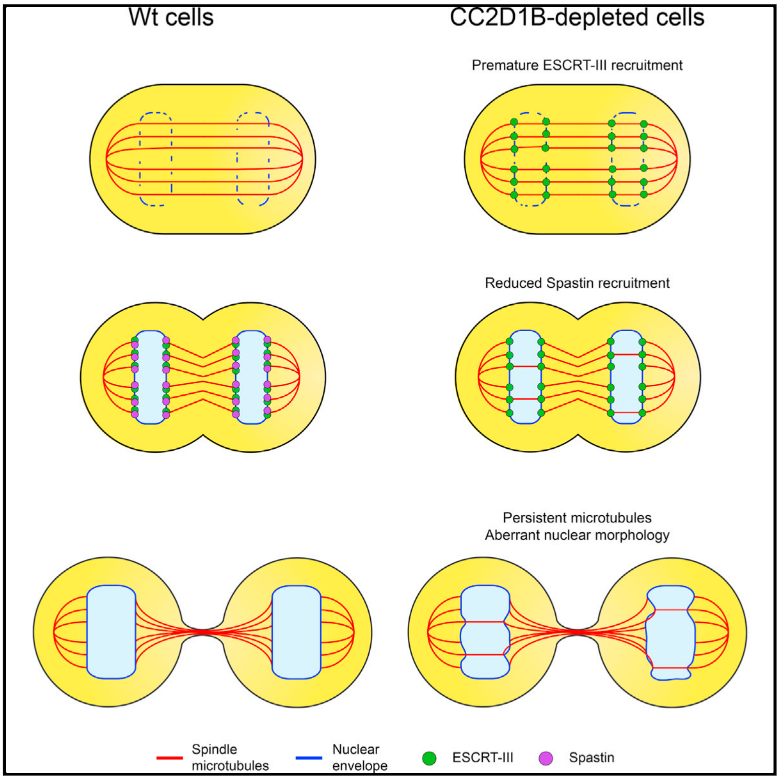

Authors

Leandro N. Ventimiglia,
Miguel Angel Cuesta-Geijo,
Nicolas Martinelli, ...,
Jeremy G. Carlton,
Winfried Weissenhorn,
Juan Martin-Serrano

Correspondence

juan.martin_serrano@kcl.ac.uk

In Brief

A faithful reformation of the nuclear envelope after mitosis is critical to ensure the proper compartmentalization of the genetic material. Ventimiglia et al. identified CC2D1B as a key regulator of this process to ensure properly timed ESCRT-III machinery recruitment and licensing of ESCRT-III filaments for nuclear envelope resealing.

Highlights

- CC2D1B localizes at the intersection between NE and microtubules at the time of nuclear resealing
- CC2D1B inhibits the polymerization of ESCRT-III *in vitro*
- CC2D1B licenses ESCRT-III filaments once ER is fully deposited on chromatin disks
- Reduced severing of spindle microtubules during NE resealing in CC2D1B-depleted cells

Ventimiglia et al., 2018, Developmental Cell *47*, 547–563
December 3, 2018 © 2018 The Author(s). Published by Elsevier Inc.
https://doi.org/10.1016/j.devcel.2018.11.012

CellPress

Developmental Cell
Article

CC2D1B Coordinates ESCRT-III Activity during the Mitotic Reformation of the Nuclear Envelope

Leandro N. Ventimiglia,[1] Miguel Angel Cuesta-Geijo,[1] Nicolas Martinelli,[2] Anna Caballe,[1,4] Pauline Macheboeuf,[2] Nolwenn Miguet,[2] Ian M. Parnham,[1] Yolanda Olmos,[3] Jeremy G. Carlton,[3] Winfried Weissenhorn,[2] and Juan Martin-Serrano[1,5,*]
[1]Department of Infectious Diseases, King's College London, Faculty of Life Sciences & Medicine, London SE1 9RT, UK
[2]CNRS, University Grenoble Alpes, CEA, Institut de Biologie Structurale (IBS), 38000 Grenoble, France
[3]Division of Cancer Studies, King's College London, London SE1 1UL, UK
[4]Present address: Sir William Dunn School of Pathology, University of Oxford, Oxford OX1 3RE, UK
[5]Lead Contact
*Correspondence: juan.martin_serrano@kcl.ac.uk
https://doi.org/10.1016/j.devcel.2018.11.012

SUMMARY

The coordinated reformation of the nuclear envelope (NE) after mitosis re-establishes the structural integrity and the functionality of the nuclear compartment. The endosomal sorting complex required for transport (ESCRT) machinery, a membrane remodeling pathway that is highly conserved in eukaryotes, has been recently involved in NE resealing by mediating the annular fusion of the nuclear membrane (NM). We show here that CC2D1B, a regulator of ESCRT polymerization, is required to re-establish the nuclear compartmentalization by coordinating endoplasmic reticulum (ER) membrane deposition around chromatin disks with ESCRT-III recruitment to the reforming NE. Accordingly, CC2D1B determines the spatiotemporal distribution of the CHMP7-ESCRT-III axis during NE reformation. Crucially, in CC2D1B-depleted cells, ESCRT activity is uncoupled from Spastin-mediated severing of spindle microtubules, resulting in persisting microtubules that compromise nuclear morphology. Therefore, we reveal CC2D1B as an essential regulatory factor that licenses the formation of ESCRT-III polymers to ensure the orderly reformation of the NE.

INTRODUCTION

Metazoan cells disassemble their nuclear envelope (NE) during prophase to undergo an open mitosis (Champion et al., 2017). The reassembly of the NE at anaphase is a highly regulated process that begins when endoplasmic reticulum (ER) membranes are delivered to the rims of naked chromatin disks (Anderson and Hetzer, 2007; Lu et al., 2011). The final steps of NE reformation comprise the severing of the spindle microtubules that persist in the newly formed nuclear compartment and the sealing of the resulting gaps in the nuclear membrane (NM) mediated by the endosomal sorting complex required for transport (ESCRT) machinery (Olmos and Carlton, 2016; Scourfield and Martin-Serrano, 2017; Vietri et al., 2016). The ESCRT-associated protein CHMP7 works as a site-specific adapter that interacts with ER membranes to reach the fenestrations left by the expanding NM around the spindle microtubules (Gu et al., 2017; Olmos et al., 2016; Vietri et al., 2015). The C-terminal region of CHMP7 is thought to trigger the polymerization of the ESCRT-III components CHMP4B and CHMP2A to seal the NE. These growing ESCRT-III filaments then recruit the AAA ATPase Spastin, a microtubule severing enzyme, thus ensuring tight coordination between spindle microtubule removal and membrane remodeling. Lastly, a second ESCRT-associated AAA ATPase, VPS4, mediates the disassembly of ESCRT-III filaments and promotes the fusion of the NM, resulting in the final sealing of the NE and the re-establishment of nuclear integrity and functionality (Olmos et al., 2015, 2016; Vietri et al., 2015). A wider role for ESCRT proteins in the maintenance of nuclear integrity has been recently established, as this pathway is required to repair ruptures in the NE arising from cell migration through narrow spaces (Denais et al., 2016; Raab et al., 2016).

Despite advances in our understanding of the main factors involved in the mitotic reformation of the NE, the mechanisms determining its spatiotemporal coordination remain elusive (Schellhaus et al., 2016; Webster and Lusk, 2016). In this work, we have explored the function of coiled-coil- and C2 domain-containing protein B (CC2D1B), a poorly characterized member of the Lgd/CC2D1 family of proteins that also includes the closely related human CC2D1A and its *D. melanogaster* ortholog Lgd. These proteins are characterized by the presence of four tandem DM14 domains that interact with the CHMP4 family of ESCRT-III proteins (Martinelli et al., 2012; Troost et al., 2012; Usami et al., 2012) and one C-terminal C2 domain that mediates their binding to membrane lipids (Drusenheimer et al., 2015; Gallagher and Knoblich, 2006). In *D. melanogaster*, Lgd regulates Notch signaling through the modulation of intracellular trafficking (Childress et al., 2006; Gallagher and Knoblich, 2006; Jaekel and Klein, 2006). Furthermore, the loss of Lgd function drives the over-proliferation of the imaginal discs, a phenotype that can be rescued by the expression of human CC2D1A or CC2D1B,

This is an open access article under the CC BY license (http://creativecommons.org/licenses/by/4.0/).

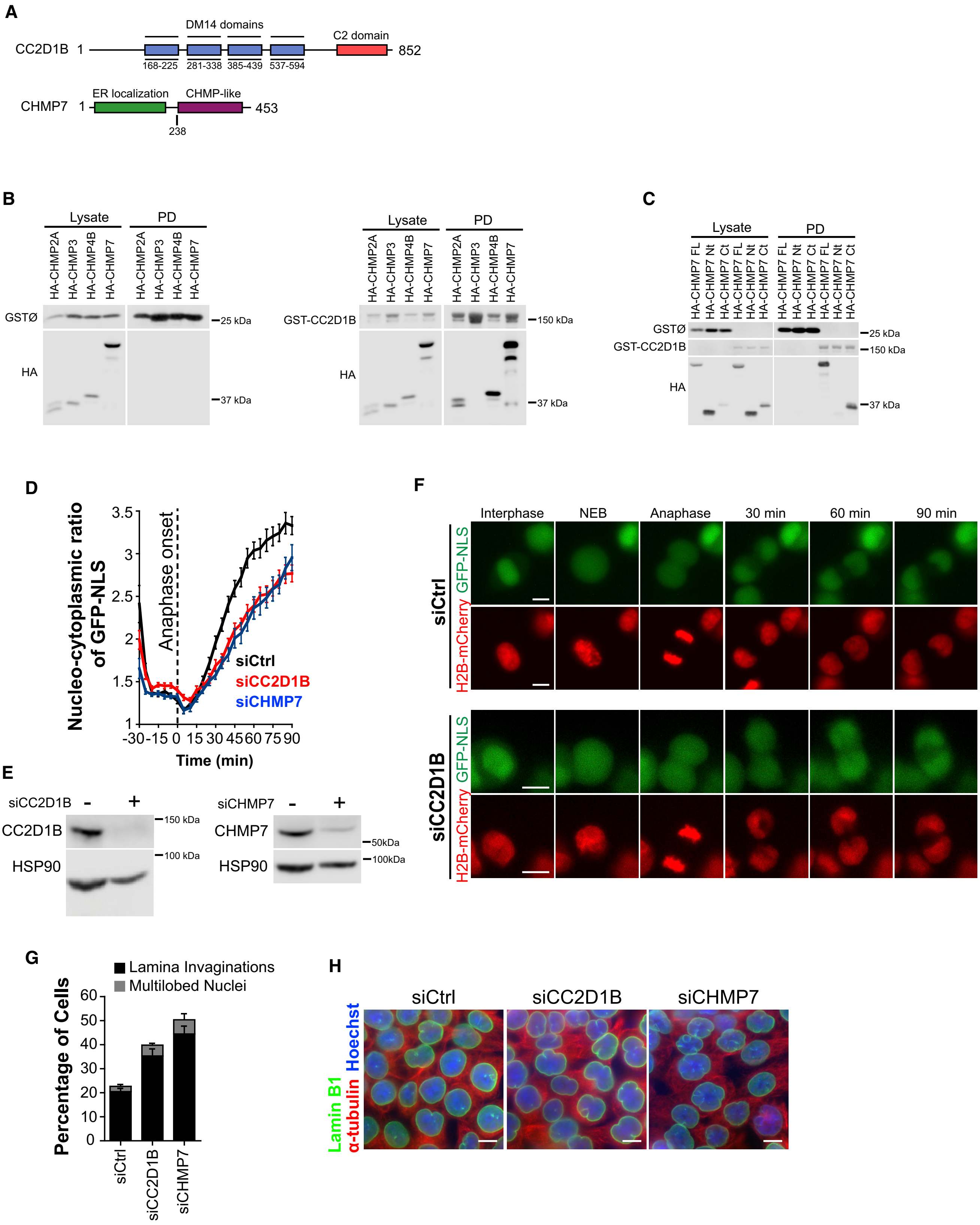

(legend on next page)

indicating a functional overlap between these proteins (Drusenheimer et al., 2015). This redundancy was also observed in mammal models, as double CC2D1A/CC2D1B knockout mice died early during embryonic development (Zamarbide et al., 2018), but the deletion of CC2D1A or CC2D1B genes alone does not induce developmental defects (Drusenheimer et al., 2015; Zamarbide et al., 2018). However, non-redundant roles of these proteins are indicated by the perinatal death of CC2D1A-deficient mice (Al-Tawashi et al., 2012; Drusenheimer et al., 2015; Zamarbide et al., 2018).

It has been described that CC2D1A and Lgd inhibit the *in vitro* polymerization of ESCRT-III by binding to the multimerization domain of CHMP4B (Martinelli et al., 2012; McMillan et al., 2017), thus suggesting a key role for the Lgd/CC2D1 family of proteins in the regulation of ESCRT activities. Here, we show that CC2D1B interacts with CHMP7 and, in dividing cells, is recruited to the reforming NE in a CHMP7-dependent manner. Furthermore, CC2D1B depletion results in the loss of coordination between ER deposition at chromatin disks and ESCRT-III recruitment to the sites of NE sealing. Critically, the premature recruitment of ESCRT-III to the reforming NE is paralleled with the aberrant recruitment of Spastin, which results in persisting spindle microtubules that compromise the morphology of the NE. Therefore, we reveal CC2D1B as a key regulatory factor that coordinates ESCRT-III recruitment and microtubule severing with ER deposition during the mitotic reformation of the NE. This work exposes the unexpected critical role of ESCRT-III deregulation in cellular processes involving membrane remodeling.

RESULTS

CC2D1B Binds CHMP7 and Is Required for NE Resealing

The closely related CC2D1A/B proteins contain a predicted membrane-binding C2 domain and four DM14 domains that autonomously bind the core region of the CHMP4 family of ESCRT-III proteins (Martinelli et al., 2012; Usami et al., 2012) (Figure 1A). We hypothesized that CC2D1B may also interact with the C-terminal domain of CHMP7 that shows a significant similarity with CHMP4B (Horii et al., 2006). As shown in Figure 1B, GST-CC2D1B binds to HA-CHMP7, HA-CHMP4B, and HA-CHMP2A, whereas no association with HA-CHMP3 was detected. Importantly, the interaction with CC2D1B was retained by the C-terminal region of CHMP7 corresponding to the ESCRT-III-like domain but not by the CHMP7 N-terminal ER-binding domain (Figures 1A and 1C). Furthermore, we were able to detect a triple interaction between CC2D1B, CHMP4B, and CHMP7 in pull-down experiments using cells co-expressing GST-CHMP4B, HA-CHMP7, and YFP-CC2D1B (Figure S1A). Further mapping experiments showed that an HA-fused CC2D1B fragment containing only the DM14 domains (HA-CC2D1B residues 162-594) interacts with GST-CHMP4B and GST-CHMP7 (Figures S1B–S1D), whereas the deletion of the fourth DM14 domain (HA-CC2D1B residues 162–493) specifically abrogates the interaction with GST-CHMP7 (Figure S1D) without affecting the binding to GST-CHMP4B (Figure S1C). Accordingly, HA-CHMP7 was able to bind a fragment of CC2D1B containing only the fourth DM14 domain (GST-CC2D1B residues 528–594; Figure S1E). Altogether, these results indicate that CC2D1B binds to CHMP7 specifically through its DM14 4 domain, whereas the binding to CHMP4B maps to a more extended region that also includes DM14 1–3, thus providing a likely mechanism for the simultaneous binding of CC2D1B to CHMP4B and CHMP7.

The CC2D1B/CHMP7 interaction prompted us to analyze the potential function of CC2D1B in the mitotic sealing of the NE, observing that the silencing of CC2D1B delayed the recovery of nuclear GFP-NLS after cell division without altering the rate of nuclear expansion (Figures 1D–1F and S1F; Video S1). Furthermore, an increase in the proportion of cells with aberrant nuclear morphologies was also observed (Figures 1G and 1H). It has been described that these two phenotypes are associated with defects in NE reformation (Asencio et al., 2012; Olmos et al., 2015, 2016). Importantly, the effect of CC2D1B silencing on nuclear morphology was also observed in the immortalized human retina pigmented epithelial cell line RPE-1 (Figures S1G–S1I), as well as in primary human dermal fibroblast (HDF; Figures S1J–S1L) and primary mouse adult fibroblast (MAF; Figures S1M–S1O). Altogether, these results suggest that CC2D1B is required for the integrity and functionality of the nuclear compartment.

The CRISPR/Cas9 system was subsequently used to delete the CC2D1B locus in HCT116 cells. The resulting cells (HCT116$^{\delta CC2D1B}$) showed an increased proportion of nuclei with aberrant morphology compared to the parental cells (Figures 2A–2C). Importantly, this phenotype was rescued upon expression of a GFP-CC2D1B fusion (HCT116$^{\delta CC2D1B}$/GFP-CC2D1B cells), allowing us to discard off-target effects in HCT116$^{\delta CC2D1B}$ cells. The full rescue activity of GFP-CC2D1B

Figure 1. CC2D1B Is Required to Maintain NE Integrity and Functionality

(A) Schematic representation of CC2D1B and CHMP7.

(B) GST pull-down experiments of 293T cells transiently co-expressing GST-CC2D1B together with HA-tagged CHMP2A, CHMP3, CHMP4B, or CHMP7.

(C) GST pull-down experiments of 293T cells transiently co-expressing GST-CC2D1B together with HA-tagged CHMP7 full-length, CHMP7 Nt (residues 1–238), or CHMP7 Ct (residues 238–453).

(D–F) Time-lapse analysis of HCT116 cells stably co-expressing GFP-NLS and H2B-mCherry and transfected with control, CHMP7 or CC2D1B siRNAs. As the recovery of nuclear integrity after cell division was previously described to be dependent on CHMP7 expression (Olmos et al., 2016), CHMP7 silencing was used to validate our experiments. (D) Representation of the nucleo-cytoplasmic GFP-NLS ratio over time. Mean ± SEM; siControl n = 29; siCC2D1B n = 35, p = 0.0003; siCHMP7 n = 22, p = 0.0402. Significance compared with the control was calculated at 90 min using a two-tailed unpaired t test. (E) Representative WB of total cell lysates corresponding to siCC2D1B or siCHMP7 treated cells. (F) Representative frames corresponding to time-lapse images of siControl (top panels) or siCC2D1B (bottom panels) treated cells. Scale bars, 10 μm.

(G and H) Analysis of nuclear morphology in siRNA transfected HCT116 cells. (G) Percentage of cells showing an aberrant nuclear morphology. Mean ± SEM; siControl n = 2371; siCC2D1B n = 2040, p = 0.0102; siCHMP7 n = 1887, p = 0.0024. Significance of NE invaginations compared to the control was calculated using a two-tailed unpaired t test. (H) Representative images corresponding to the quantifications shown in (G). Scale bars, 10 μm.

See also Figure S1 and Video S1.

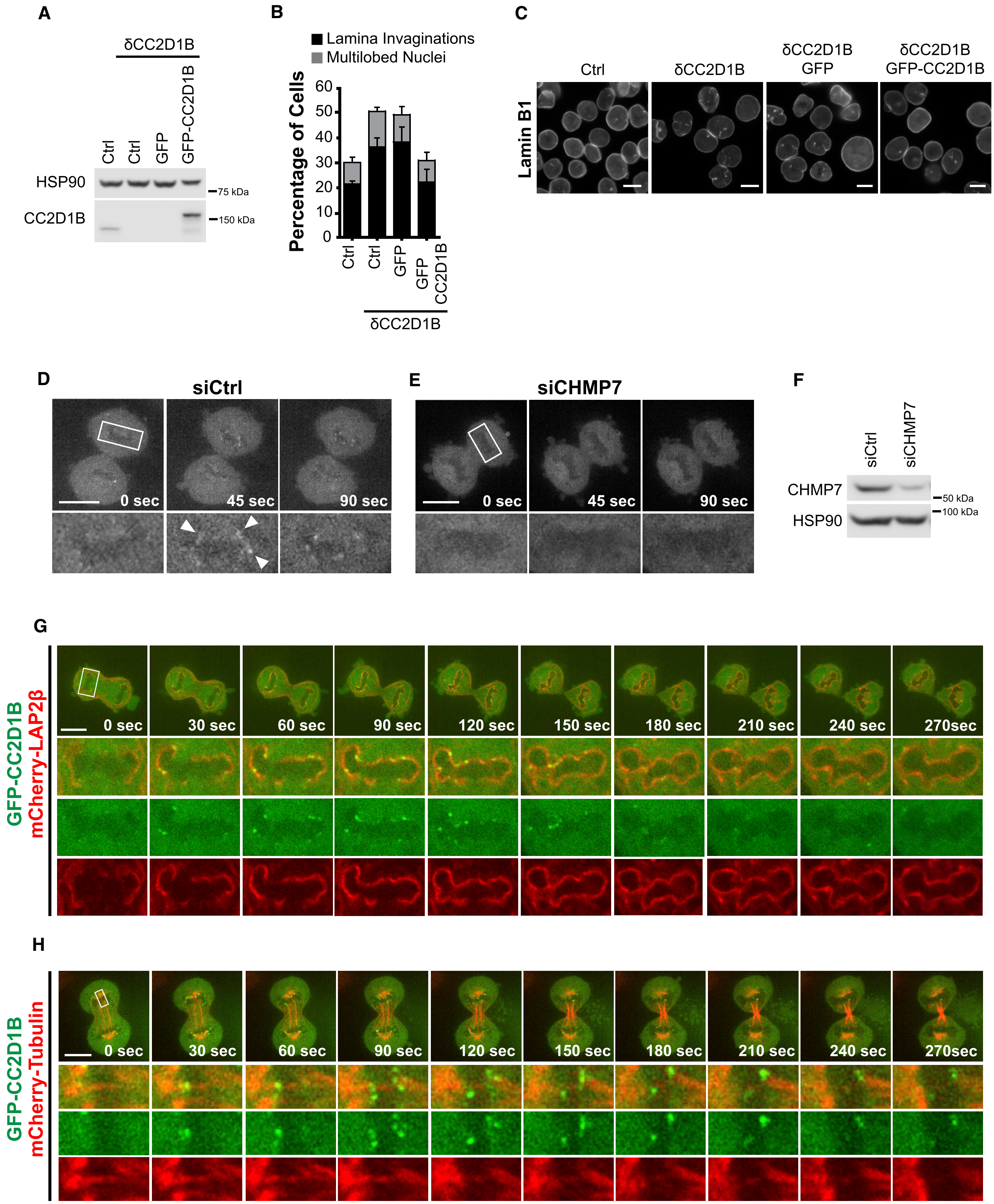

Figure 2. CC2D1B Is Recruited to the Reforming NE in a CHMP7-Dependent Way

(A–C) Characterization of HCT116$^{\delta CC2D1B}$ cells. (A) Total cell lysates corresponding to HCT116 Control, HCT116$^{\delta CC2D1B}$, HCT116$^{\delta CC2D1B}$/GFP and HCT116$^{\delta CC2D1B}$/GFP-CC2D1B were analyzed by WB using an anti-CC2D1B antibody. (B) Analysis of nuclear morphology in HCT116$^{\delta CC2D1B}$ cells. Mean ± SEM;

(legend continued on next page)

also highlighted that expression of this fusion in HCT116$^{\delta CC2D1B}$ cells provides a relevant system to determine the localization of CC2D1B in the context of mitotic NE reformation. Then, we analyzed HCT116$^{\delta CC2D1B}$/GFP-CC2D1B cells by time-lapse spinning disk confocal microscopy, observing that GFP-CC2D1B transiently accumulated in the perinuclear region during telophase (Figure 2D; Video S2). Remarkably, this enrichment was abrogated upon depletion of CHMP7 (Figures 2E and 2F; Video S2) in agreement with the CHMP7/CC2D1B interaction described above. The localization of GFP-CC2D1B on the reforming NE was further confirmed in cells co-expressing the NE marker lamina-associated polypeptide 2β fused to mCherry (mCherry-LAP2β) (Figure 2G; Video S3). The co-localization of GFP-CC2D1B with mCherry-tubulin was then explored, observing that CC2D1B decorates spindle microtubules, frequently marking the site of subsequent microtubule severing (Figure 2H; Video S4). As the intersection between the NE and spindle microtubules defines the membrane gaps sealed by ESCRT-III (Vietri et al., 2015), our results suggest that CC2D1B may be part of the ESCRT-associated machinery recruited by CHMP7 to seal the NE during mitosis.

The Role of CC2D1B on NE Reformation Depends on Its Ability to Bind Membrane Lipids

We then explored functional determinants shared by the Lgd/CC2D1 family, thereby performing structural studies of the highly conserved C-terminal region of the *Drosophila* ortholog Lgd (Figure S2A). The structure revealed an α-helical N-terminal domain that contains a ~50 Å long helical hairpin, followed by a long coil structure arranged anti-parallel to the hairpin which connects to the C2 domain at amino acid 664 (Figures 3B and 3C). The C2 fold ends at amino acid 796 and the remaining C-terminal region (residues 797–815) intertwines with the N-terminal helical domain, thereby stabilizing it (Figures 3B and S2B). The C2 domain of Lgd forms a classical β-sandwich structure with two four-stranded β sheets that classifies as a class II C2 domain with N and C termini oriented at the bottom of the sandwich and opposite to the top loops (Figure 3C), similar to the topology of the phospholipase C-δ1 C2 domain (Essen et al., 1996). The Lgd C2 domain is atypical as two short α helices are inserted into the loops connecting β strands 5 with 6 (α3) and β- 7 with 8 (η2). These α helices are conserved in human CC2D1A/B orthologs (Figure S2A), suggesting that these regions are functionally important. The convex part of the C2 domain is rigidly connected to the N-terminal α-helical domain via multiple interactions (Figure S2B). In contrast, the opposite concave part is solvent exposed. Intriguingly, the Lgd helical hairpin is structurally similar to the Lgd DM14 3 domain with an root-mean-square deviation (RMSD) of 2.6 Å (Figure S2C), although its orientation toward the C2 domain would sterically prevent CHMP4B/shrub binding (McMillan et al., 2017). The hairpin shows further significant structural similarity with ESCRT-III CHMP3, CHMP4, and Ist1 (Bajorek et al., 2009; Martinelli et al., 2012; Muzioł et al., 2006; Xiao et al., 2009) with RMSD's of 3.0–3.1 Å on ~70 equivalent Cα atoms, perhaps suggesting further associations with the ESCRT machinery.

The C2 fold described above suggested a role of the Lgd C terminus in lipid binding, a notion consistent with the previously described interaction of Lgd with phosphoinositides (Gallagher and Knoblich, 2006). Therefore, we performed a stringent pull-down assay to determine the binding of Lgd (residues 358–816) to a phosphoinositide panel, observing a weak but significant interaction with phosphatidylinositol 4,5-biphosphate (PIns(4,5)P_2) (Figure S2D). This result is supported by our structural data as the C2 domain of Lgd shows strong similarities with the C2 domains of protein kinase Cα (PKCα) and synaptotagmin (Figure S2E) that bind to PIns(4,5)P_2 through a basic cluster that makes direct contacts with the phosphates of the inositide ring (Guerrero-Valero et al., 2009; Guillén et al., 2013). Interestingly, this cluster is conserved in Lgd (residues R689, K701, K703) as well as in eukaryotic orthologs including human CC2D1B (Figures S2A and S2E), which is consistent with the ability of CC2D1A and CC2D1B to associate with membrane pellets in cell fractioning experiments (Drusenheimer et al., 2015).

These structural and biochemical studies provided the basis to assess the functional contribution of the C2 domain of CC2D1B to the mitotic sealing of the NE. Therefore, a triple mutation (R721A, K734A, K736A) disrupting the putative phosphoinositide binding site in CC2D1B (Figure 3D) was engineered, and the resulting mutant (CC2D1B-C2M) was fused to GFP and stably expressed in HCT116$^{\delta CC2D1B}$ cells. Importantly, GFP-CC2D1B Wt and GFP-CC2D1B-C2M were expressed at comparable levels in a cellular background that does not express endogenous CC2D1B (Figure 3E; lanes 3 and 4). The time-lapse analysis of these cells indicated that GFP-CC2D1B-C2M recruitment to the reforming NE was abrogated (Figure 3F, bottom panels, and Video S5). Furthermore, we detected PIns(4,5)P_2 foci colocalizing with CHMP4B-L-GFP on the NE of dividing cells, supporting a lipid-mediated recruitment of CC2D1B to the resealing NE (Figures 3G and S2F). Remarkably, the mislocalization of GFP-CC2D1B-C2M correlated with its inability to rescue the rate of mCherry-NLS nuclear recovery after mitosis (Figure 3H), whereas GFP-CC2D1B Wt showed full rescue activity in this assay. Accordingly, GFP-CC2D1B-C2M failed to lower the high proportion of HCT116$^{\delta CC2D1B}$ cells with aberrant nuclear morphology (Figures 3I and S2G). These results indicate that the recruitment of CC2D1B to the reforming NE depends on its ability to bind membrane lipids and suggest that this recruitment is essential to re-establish the nuclear compartmentalization after cell division.

HCT116 Control n = 628; HCT116$^{\delta CC2D1B}$ n = 608, p = 0.0162; HCT116$^{\delta CC2D1B}$/GFP n = 593, p = 0.0553; HCT116$^{\delta CC2D1B}$/GFP-CC2D1B n = 603, p = 0.9058. Significance of NE invaginations compared to the control was calculated using a two-tailed unpaired t test. (C) Representative images corresponding to the quantifications shown in (B). Scale bars, 10 μm.

(D–F) Time-lapse images of HCT116$^{\delta CC2D1B}$/GFP-CC2D1B cells transfected with control (D) or CHMP7 siRNAs (E). Arrowheads indicate examples of GFP-CC2D1B perinuclear accumulation. Scale bars, 10 μm. (F) Representative WB corresponding to the cells shown in (D) and (E).

(G) Time-lapse images of HCT116$^{\delta CC2D1B}$/GFP-CC2D1B cells stably co-expressing a mCherry-Lap2B fusion. Scale bar, 10 μm.

(H) Time-lapse images of HCT116$^{\delta CC2D1B}$/GFP-CC2D1B cells stably co-expressing a mCherry-tubulin fusion. Scale bar, 10 μm.

See also Videos S2, S3, and S4.

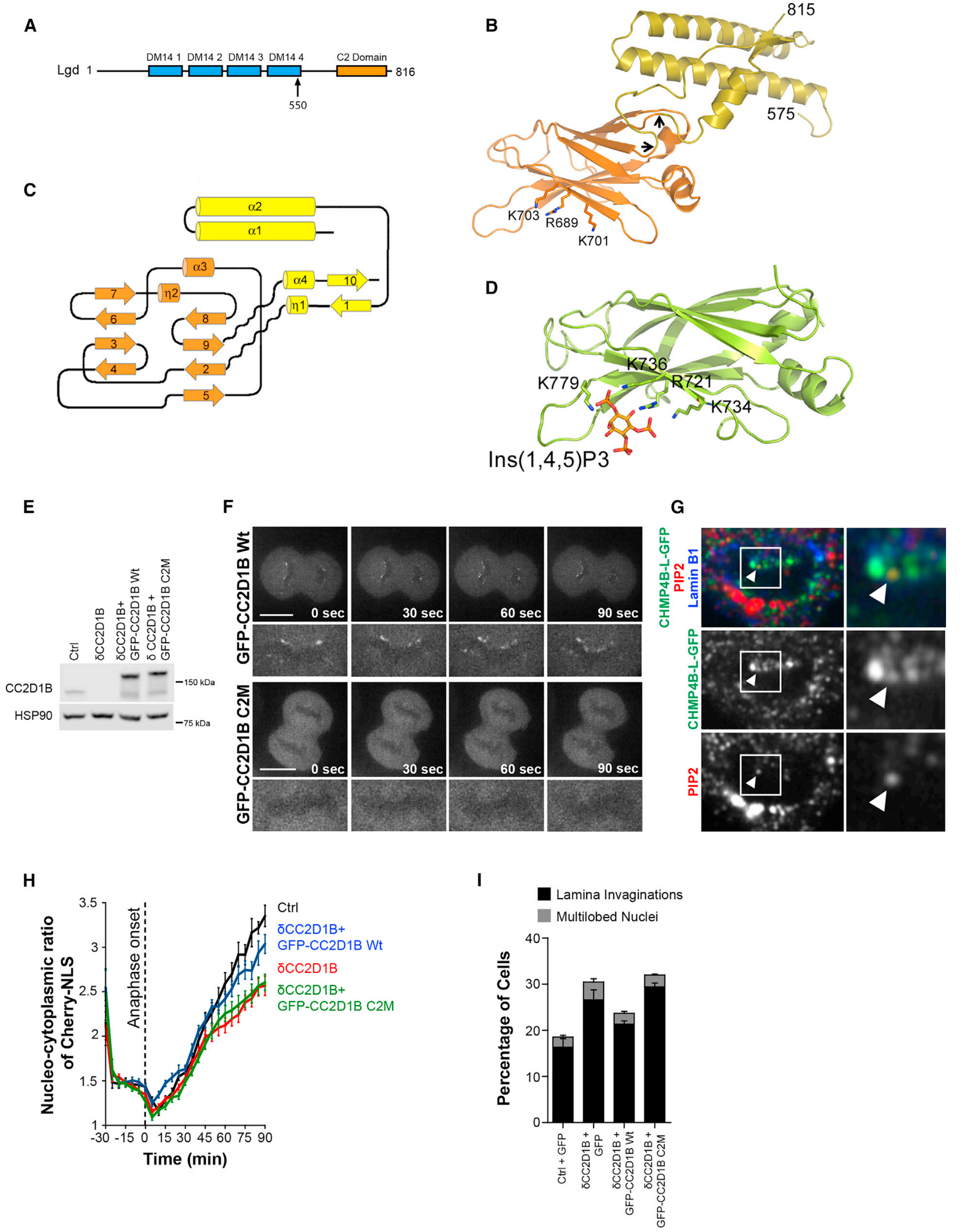

(legend on next page)

CC2D1B Determines the Timely Recruitment of ESCRT Proteins to the Resealing NE

The localization of CC2D1B on the reforming NE prompted us to investigate whether this protein may be involved in the coordination of ESCRT activity during nuclear reformation. To analyze the dynamics of ESCRT-III subunits during this process, we fused CHMP4B or CHMP2A to a 25-nm flexible linker (Kim et al., 2016) followed by GFP. The resulting CHMP4B-L-GFP and CHMP2A-L-GFP constructs were expressed at sub-physiological levels in HCT116 cells, and the initial characterization indicated that these fusions showed the expected localization and activity as they localized at the midbody (Figure S3A); their expression did not delay midbody abscission time (Figure S3B); they were recruited to the reforming NE in a CHMP7-dependent manner (Figures S3C–S3F; Videos S6 and S7), and they relocalized from a diffuse cytoplasmic pattern to the surface of aberrant endosomes upon depletion of VPS4A/B (Figure S3G). Moreover, pull-down analysis showed the specific association of endogenous CC2D1B with CHMP4B-L-GFP although not with CHMP2A-L-GFP (Figure S3H). Thus, cells stably expressing CHMP4B-L-GFP or CHMP2A-L-GFP are suitable models to analyze the role of CC2D1B in the dynamic localization of ESCRT-III during NE reformation.

We next developed an imaging based semi-quantitative approach to determine the dynamics of ESCRT recruitment to the reforming NE (Figures 4A–4D; Videos S8, S9, and S10). Notably, the recruitment of ESCRT-III subunits was tightly coordinated in control cells, as GFP-CHMP7 and CHMP4B-L-GFP signals peaked at 370 s after cleavage furrow ingression, while the CHMP2A-L-GFP signal peaked closely at 290 s (Figure 4D, top panel). Strikingly, CC2D1B depletion resulted in the premature and asynchronous recruitment of ESCRTs (Figure 4D, bottom panel, and S4A), as GFP-CHMP7 recruitment occurred more than 4 min earlier in CC2D1B-silenced cells, and the accumulation of GFP-CHMP7, CHMP4B-L-GFP, and CHMP2A-L-GFP at the NE overlapped with the beginning of cleavage furrow ingression. Furthermore, the coordination between ESCRT proteins was lost in CC2D1B-depleted cells, as evidenced by the 2-min difference between the peak recruitment of GFP-CHMP7 (100 s) and CHMP4B-L-GFP (220 s). To validate the use of the furrow ingression as a suitable time marker for the quantification of ESCRT-III recruitment, we analyzed the effect of CC2D1B depletion on the time gap between anaphase onset and the beginning of furrow ingression, observing that the silencing of CC2D1B increases this gap by 1 min when compared to control cells (Figures S4B–S4D; Video S11). However, although this result suggests a modest effect of CC2D1B deletion on mitosis timing, this difference is not sufficient to completely explain the early recruitment of ESCRT-III in CC2D1B-silenced cells. The premature recruitment of ESCRT subunits to the NE was also confirmed in cells stained for endogenous CHMP2A (Figure 4E). As the distance between both chromosome sets increases during anaphase, the midzone length was used as a surrogate marker for the timing of CHMP2A recruitment. We observed that cells with CHMP2A at chromatin disks showed a shorter midzone length upon depletion of CC2D1B (Figure 4F), thus supporting the notion that CHMP2A recruitment to the reforming NE occurs earlier in these cells.

We next quantified the area under the fluorescence recruitment curves of GFP-CHMP7, CHMP4B-L-GFP, and CHMP2A-L-GFP as an indication of the total amount of ESCRT proteins recruited to the resealing NE. We observed that the silencing of CC2D1B had no effect on the total amount of GFP-CHMP7, CHMP4B-L-GFP, or CHMP2A-L-GFP fluorescence recruited (Figure S4E). As quantification of total fluorescence recruitment does not allow the discrimination between parameters as the number of ESCRT foci recruited and their persistence, we analyzed these parameters individually. We observed that the silencing of CC2D1B does not affect the number of GFP-CHMP7, CHMP4B-L-GFP, and CHMP2A-L-GFP foci recruited to the resealing NE (Figure S4F). Finally, we observed that, although the depletion of CC2D1B does not significantly alter the persistence time of GFP-CHMP7 and CHMP4B-L-GFP fluorescence on the NE, it increases the persistence time of CHMP2A-L-GFP (Figure S4G), perhaps suggesting the abortive formation of CHMP2A-containing polymers. Altogether, these

Figure 3. A Functional C2 Domain Is Required for Proper Localization and Functionality of CC2D1B

(A) Schematic representation of Lgd. The arrow marks the first residue of the solved structure.

(B) Ribbon representation of Lgd C-terminal residues 575–816. The crystal structure was solved from a selenomethionine substituted crystal using the single wavelength anomalous dispersion method and the model refined to a resolution of 2.4 Å (Table S1) included the predicted C2 domain and the preceding region that lacks obvious sequence homology with known functional domains (residues 550–816). The helical domain composed of the N- and C-terminal region is shown in yellow and the C2 domain in orange. Conserved basic residues implicated in PIns(4,5)P2 interaction are shown as sticks. The N-terminal 24 residues connecting to the DM14 4 domain and present in the crystallized construct are flexible and disordered in the structure. Arrows indicate the site of insertion of the helical domain into the C2 domain.

(C) Topology diagram of Lgd (residues 575–816) highlighting the insertion of the C2 domain into the helical domain.

(D) Homology model of CC2D1B based on Lgd structure and docking of PIns(1,4,5)P_3. The basic residues involved in PIns binding are shown as sticks.

(E) Total cell lysates corresponding to HCT116 Control, HCT116$^{\delta CC2D1B}$, HCT116$^{\delta CC2D1B}$/GFP-CC2D1B Wt, and HCT116$^{\delta CC2D1B}$/GFP-CC2D1B C2M were analyzed by WB using an anti-CC2D1B antibody.

(F) Time-lapse analysis of HCT116$^{\delta CC2D1B}$/GFP-CC2D1B Wt (top panels) or C2M (bottom panels) cells. Scale bars, 10 μm.

(G) Nuclear inset of an HCT116 cell stably expressing CHMP4B-L-GFP and stained with anti-GFP, -PIP2 and -Lamin B1 antibodies.

(H and I) Functional analysis of HCT116$^{\delta CC2D1B}$/GFP-CC2D1B C2M cells. (H) Representation of the nucleo-cytoplasmic ratio of mCherry-NLS over time in HCT116$^{\delta CC2D1B}$ cells stably co-expressing GFP-CC2D1B Wt or C2M. Cells were incubated with Hoechst 33258 30 min before imaging to stain DNA. Mean ± SEM; HCT116 Control n = 14; HCT116$^{\delta CC2D1B}$ n = 25, $p < 0.0001$; HCT116$^{\delta CC2D1B}$/GFP-CC2D1B Wt n = 18, p = 0.0597; HCT116$^{\delta CC2D1B}$/GFP-CC2D1B C2M n = 20, $p < 0.0001$. Significance compared to the control was calculated at 90 min using a two-tailed unpaired t test. (I) Analysis of nuclear morphology of HCT116$^{\delta CC2D1B}$ cells stably expressing GFP-CC2D1B Wt or C2M. Mean ± SEM; HCT116 Control n = 1,055; HCT116$^{\delta CC2D1B}$ n = 911, p = 0.0025; HCT116$^{\delta CC2D1B}$/GFP-CC2D1B Wt n = 896, p = 0.0651; HCT116$^{\delta CC2D1B}$/GFP-CC2D1B C2M n = 976, p = 0.0030. Significance of NE invaginations compared to the control was calculated using a two-tailed unpaired t test.

See also Figure S2, Video S5, and Table S1.

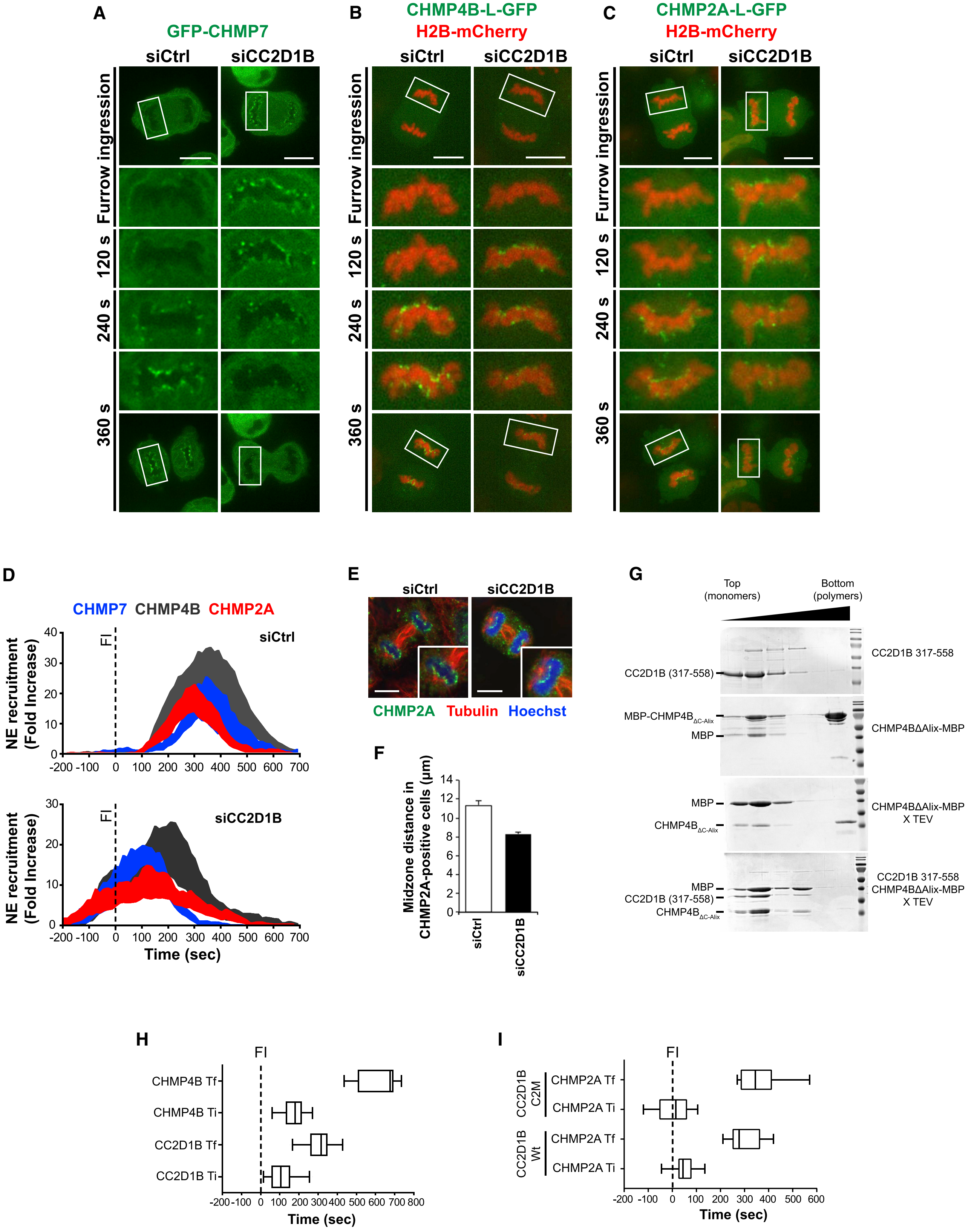

(legend on next page)

results indicate that the silencing of CC2D1B not only affects the timing of ESCRT-III recruitment to the resealing NE but could also have consequences on the quality of ESCRT-III polymers, suggesting that this machinery could be unable to fulfill its function in CC2D1B-depleted cells.

We then investigated whether CC2D1B regulates ESCRT-III polymerization using an *in vitro* assay previously described (Martinelli et al., 2012). Briefly, a C-terminal truncation of CHMP4B fused to MBP (MBP-CHMP4B$_{\Delta C\text{-}Alix}$) produces monomers (top fractions) and CHMP4B filamentous polymers (bottom fraction) (Pires et al., 2009) when analyzed by sucrose gradient density centrifugation. Upon cleavage of monomeric MBP-CHMP4B$_{\Delta C\text{-}Alix}$ by tobacco etch virus (TEV) protease, CHMP4B becomes predominantly polymeric, whereas MBP stays in the upper monomeric fractions. Importantly, cleavage of monomeric MBP-CHMP4B$_{\Delta C\text{-}Alix}$ in the presence of a fragment of CC2D1B (residues 317–558) containing the third DM14 domain prevented the accumulation of CHMP4B$_{\Delta C\text{-}Alix}$ in the polymeric form (Figure 4G). These data suggest that CC2D1B inhibits CHMP4B polymerization *in vitro*, supporting a model whereby ESCRT-III polymerization is directly regulated by CC2D1B during the resealing of the NE.

To gain more mechanistic insights into the role of CC2D1B on the recruitment of ESCRT-III to the reforming NE, we compared the timing of GFP-CC2D1B and CHMP4B-L-GFP recruitment. We observed that, although both proteins are recruited roughly at the same time, CC2D1B disappeared from the reforming NE approximately 5 min before CHMP4B (Figure 4H), suggesting that CC2D1B could be specifically involved in the regulation of the early stages of ESCRT polymerization during NE reformation. We also asked whether the ability of CC2D1B to bind membrane phospholipids could play a role in the timing of ESCRT-III recruitment. Then, we analyzed the recruitment of CHMP2A-L-mCherry in HCT116$^{\delta CC2D1B}$ cells co-expressing GFP-CC2D1B Wt (Figure S4H; Video S12) or C2M (Figure S4I; Video S12), observing that the timing of CHMP2A-L-mCherry recruitment is not significantly different in these cells lines (Figure 4I). Thus, the timely recruitment of ESCRT-III to the NE is independent of the ability of CC2D1B to bind membrane phospholipids.

CC2D1B Coordinates NM Deposition with ESCRT-III Recruitment to the NE

To exclude general perturbations during early stages of NE reformation as consequence of CC2D1B silencing, we analyzed ER deposition around chromatin in HeLa cells co-expressing H2B-mCherry and the ER membrane-associated protein Sec61β fused to YFP (Figures 5A and S5A; Video S13). Although CC2D1B depletion resulted in the premature recruitment of CHMP4B-L-GFP (Figures 5B, 5C, bottom panel, and S5B; Video S13), in line with our results using HCT116 cells, no effect on the dynamics of NM deposition was observed (Figure 5A, 5C, top panel, and S5A). Notably, a tight coordination between NE reformation and ESCRT-III recruitment was evident in control cells, as CHMP4B-L-GFP peak recruitment (Figure 5C, bottom panel, black curve) occurred once ER deposition reached a plateau (Figure 5C, top panel, black line), which indicated that NE reformation is completed. In contrast, the peak recruitment of CHMP4B-L-GFP (Figure 5C, bottom panel, red curve) overlapped with the NE deposition phase (Figure 5C, top panel, red line) in CC2D1B-depleted cells, supporting the hypothesis of a premature recruitment of ESCRT-III in cells lacking CC2D1B expression.

To confirm these observations, we analyzed the dynamics of NE deposition and ESCRT-III recruitment by super resolution time-lapse microscopy using HCT116 cells stably co-expressing CHMP4B-L-GFP and the NE marker Emerin fused to mCherry (Figures 5D, 5E, and S5C–S5E; Video S13), observing that in control cells the recruitment of CHMP4B to the resealing NE began when mCherry-Emerin covered a high percentage of the nuclear perimeter (median value = 89.61%). In contrast, CC2D1B-depleted cells showed CHMP4B puncta decorating the NE in earlier stages of nuclear reformation, when the reforming NE covered a lower percentage of nuclear perimeter (median value = 72.6%). We observed a similar result in HCT116 cells

Figure 4. CC2D1B Organizes the Timely Recruitment of ESCRT-III Proteins to the Reforming NE

(A–C) Time-lapse analysis of HCT116 cells stably expressing GFP-CHMP7 (A), or stably co-expressing CHMP4B-L-GFP (B) or CHMP2A-L-GFP (C) along with H2B-mCherry. Cells were transfected with control or CC2D1B siRNAs. Scale bars, 10 μm in (A), (B), and (C).

(D) Quantification of GFP-CHMP7, CHMP4B-L-GFP, and CHMP2A-L-GFP recruitment to the reforming NE in HCT116 cells transfected with control (top panel) or CC2D1B (bottom panel) siRNAs. T0 was set at the beginning of furrow ingression (FI). Curves indicate mean ± SEM. GFP-CHMP7: siControl n = 7; siCC2D1B n = 7. CHMP4B-L-GFP: siControl n = 11; siCC2D1B n = 13. CHMP2A-L-GFP: siControl n = 15; siCC2D1B n = 15.

(E and F) Recruitment of endogenous CHMP2A to the reforming NE in HCT116 fixed cells. (E) Representative images of HCT116 cells transfected with control or CC2D1B siRNAs and stained with anti-CHMP2A and anti-tubulin antibodies. (F) Quantification of the midzone distance in CHMP2A-positive cells. Mean ± SEM; siControl n = 29; siCC2D1B n = 32, p = 0.0004. Scale bars, 10 μm in (E).

(G) Sucrose gradient analysis of MBP-CHMP4B$_{\Delta C\text{-}Alix}$ polymerization in the presence of CC2D1B (residues 317–558). Upper panel, CC2D1B (residues 317–558) floats in the top fractions of the gradient consistent with being monomeric; second panel, MBP-CHMP4B$_{\Delta C\text{-}Alix}$ is found in the top fractions (monomers) and in the bottom (polymers) fraction; third panel, TEV cleavage of monomeric MBP-CHMP4B$_{\Delta C\text{-}Alix}$ induces polymerization as indicated by the band in the bottom fraction; lower panel, TEV cleavage of monomeric MBP-CHMP4B$_{\Delta C\text{-}Alix}$ in the presence of CC2D1B (residues 317–558) retains CHMP4B$_{\Delta C\text{-}Alix}$ in the monomer fractions.

(H) Comparison of CC2D1B and CHMP4B recruitment times. HCT116 cells expressing GFP-CC2D1B or CHMP4B-L-GFP were analyzed by time-lapse microscopy and the first (Ti) and the last (Tf) frames showing GFP-CC2D1B or CHMP4B-L-GFP accumulation in the perinuclear area were scored. Whiskers mark 5–95 percentiles. The movies used to quantify CHMP4B-L-GFP Ti and Tf were the same used for the recruitment quantification shown in Figure 4D. T0 was set at the beginning of furrow ingression (FI). GFP-CC2D1B n = 29; Ti p = 0.0183; Tf p < 0.0001. Significance compared to the control was calculated using a two-tailed unpaired t test.

(I) Analysis of GFP-CC2D1B C2M expression on ESCRT-III recruitment time. HCT116 cells co-expressing CHMP2A-L-mCherry and GFP-CC2D1B Wt or C2M were analyzed by time-lapse microscopy and the first (Ti) and the last (Tf) frames showing CHMP2A-L-mCherry accumulation in the perinuclear area were scored. Whiskers mark 5–95 percentiles. T0 was set at the beginning of furrow ingression (FI). GFP-CC2D1B Wt n = 10; GFP-CC2D1B C2M n = 13; Ti p = 0.0803; Tf p = 0.0638. Significance compared to the control was calculated using a two-tailed unpaired t test.

See also Figure S4 and Videos S8–S12.

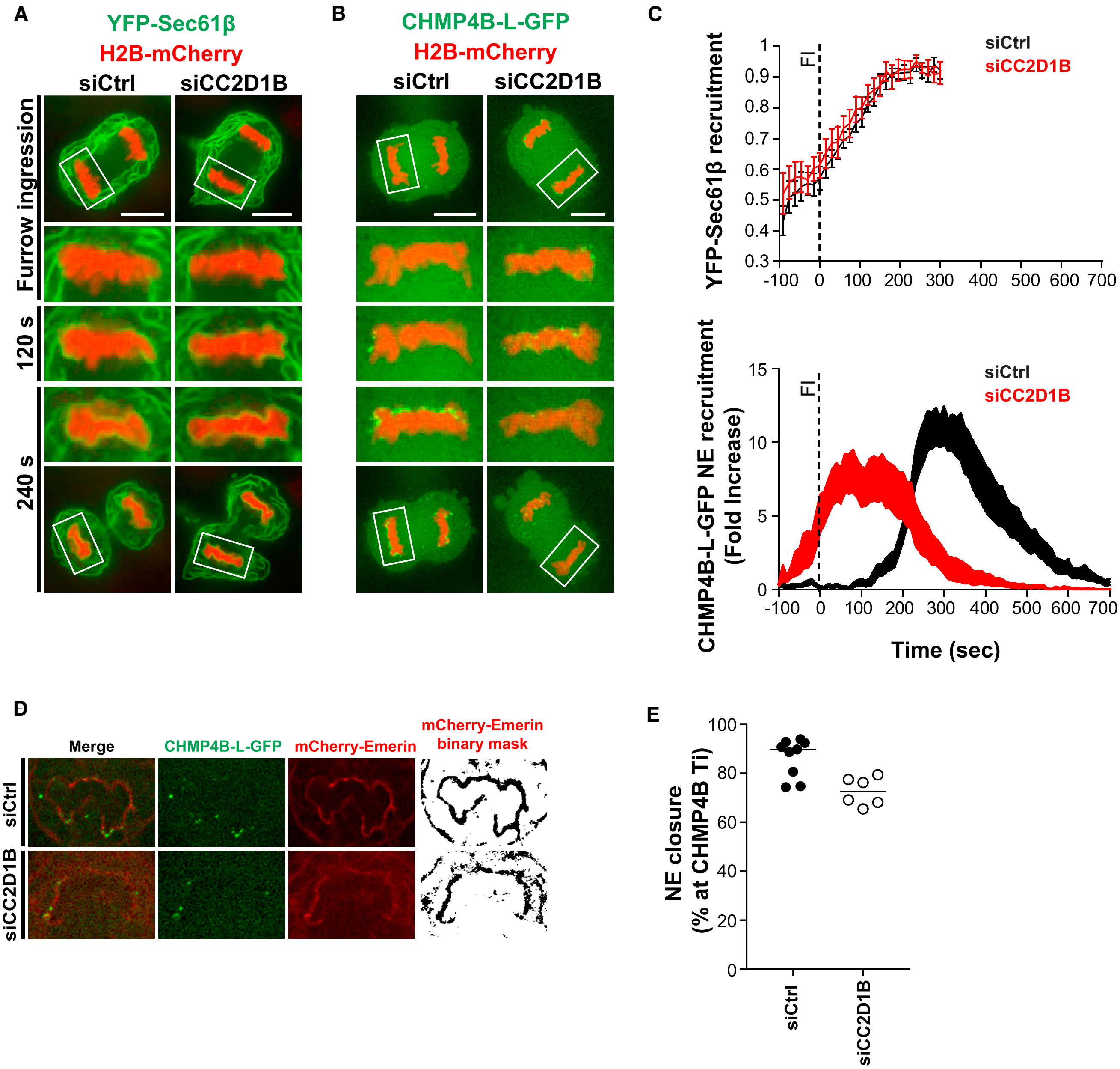

Figure 5. CC2D1B Coordinates NM Deposition with ESCRT-III Recruitment to the Resealing NE

(A) Time-lapse analysis of HeLa cells stably co-expressing YFP-Sec61β and H2B-mCherry. Cells were transfected with control or CC2D1B siRNAs. Scale bars, 10 μm.

(B) Time-lapse analysis of HeLa cells stably co-expressing CHMP4B-L-GFP and H2B-mCherry. Cells were transfected with control or CC2D1B siRNAs. Scale bars, 10 μm.

(C) Quantification of YFP-Sec61β (Top panel. Lines: mean ± SEM) or CHMP4B-L-GFP (Bottom panel. Curves: mean ± SEM) recruitment to the NE in HeLa cells transfected with control or CC2D1B siRNAs. T0 was set at the beginning of furrow ingression (FI). YFP-Sec61β recruitment: siControl n = 15; siCC2D1B n = 10. CHMP4B-L-GFP recruitment: siControl n = 11; siCC2D1B n = 14.

(D) Super resolution time-lapse analysis of HCT116 cells stably co-expressing CHMP4B-L-GFP and mCherry-Emerin and transfected with control or CC2D1B siRNAs. The frames corresponding to the beginning of CHMP4B recruitment (CHMP4B Ti) to the reforming NE are shown.

(E) Quantification of the percentage of NE reformation at the beginning of CHMP4B recruitment (CHMP4B Ti). Bars indicate median. siControl n = 9; siCC2D1B n = 6, p = 0.0027. Significance compared to the control was calculated using a two-tailed unpaired t test.

See also Figure S5 and Video S13.

stably co-expressing CHMP4B-L-GFP and mCherry-Sec61β (Figure S5F; Video S13). In control cells, CHMP4B was recruited to the resealing NE once this structure was completely formed around the nascent nucleus. On the other hand, in cells silenced for CC2D1B expression, CHMP4B was recruited in earlier stages of ER deposition when the reforming NM was not yet fully

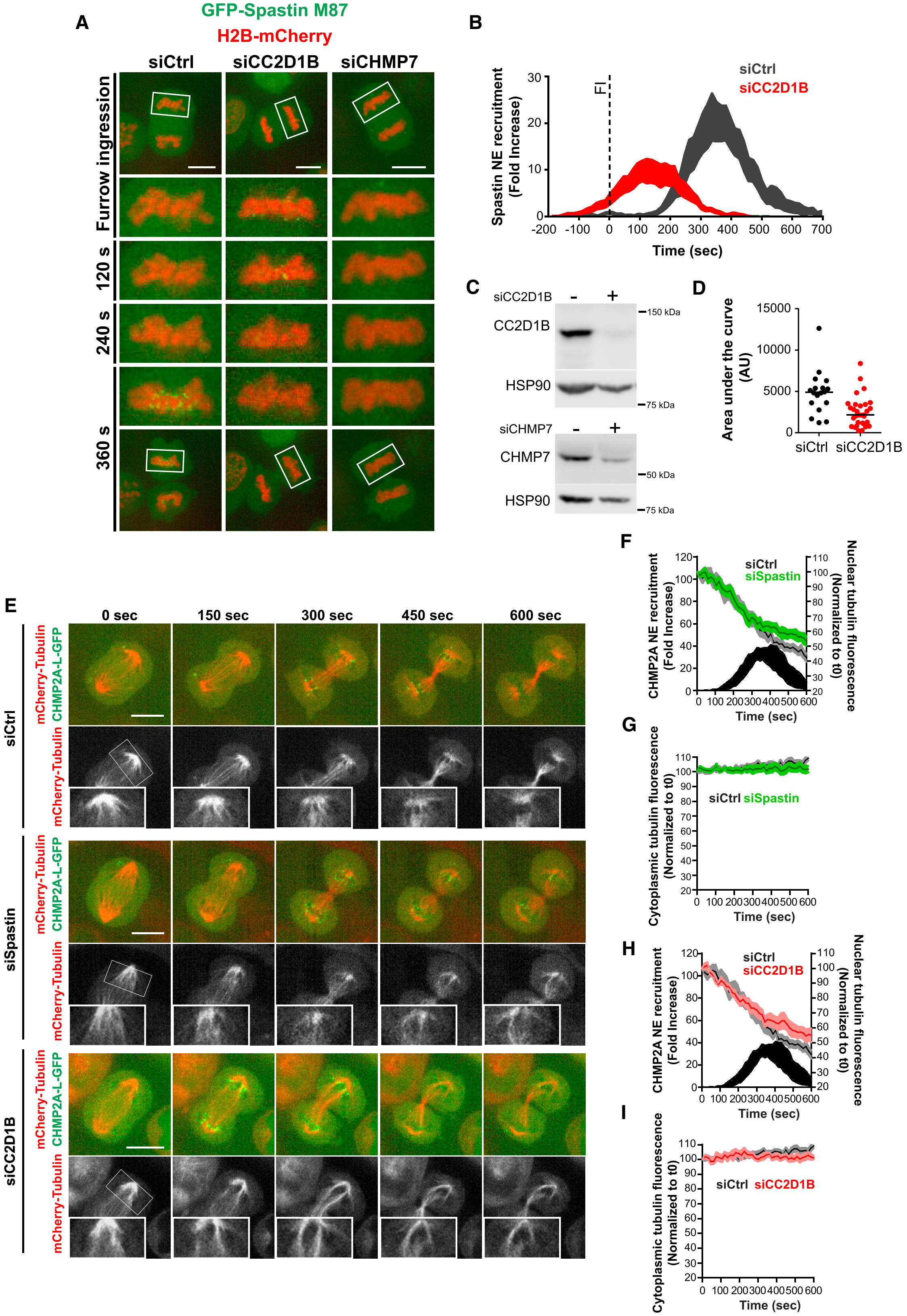

(legend on next page)

enclosing the nuclear compartment, thus supporting our quantitative data. Altogether, these results confirm that CC2D1B ensures that ESCRT-III is recruited to the reforming NE at the right time, once chromatin is fully enclosed by the NE.

CC2D1B Silencing Impairs the Severing of Spindle Microtubules

NE resealing requires the severing of microtubules at the intersection between the mitotic spindle and the NM by the ESCRT-recruited ATPase Spastin (Vietri et al., 2015). The role of CC2D1B on ESCRT-III recruitment to the reforming NE prompted us to investigate whether this protein could also be involved in Spastin recruitment. We first observed that CC2D1B silencing induced the premature recruitment of GFP-Spastin M87 to the NE (Figures 6A, middle panels, 6B and 6C, top panels; Video S14), and, as expected, this recruitment was ablated in CHMP7-depleted cells (Figure 6A, right panels, and 6C, bottom panels; Video S14). Furthermore, CC2D1B silencing induced a strong reduction in the total amount of GFP-Spastin fluorescence recruited to the NE (Figure 6D). These results suggest that the asynchronous recruitment of ESCRT-III subunits to the reforming NE in CC2D1B-depleted cells impairs the downstream recruitment of Spastin, and, consequently, the ability of these cells to sever the spindle microtubules may be compromised.

We next studied the kinetics of microtubule disassembly at the nascent nuclear compartment using HCT116 cells co-expressing mCherry-tubulin and CHMP2A-L-GFP. In agreement with previous reports (Vietri et al., 2015), CHMP2A accumulation preceded microtubule disassembly in control cells (Figure 6E, top panels; Video S15). Conversely, in Spastin-depleted cells, dense microtubule bundles connecting the midbody with the spindle pole persisted even at late stages of cell division, when CHMP2A-L-GFP has already disappeared from the perinuclear region (Figure 6E, middle panels, and S6B, left panels; Video S15). These observations were further supported by a semi-quantitative approach to estimate the disassembly rate of nuclear microtubules by quantifying mCherry-tubulin fluorescence in the nucleus over time. The silencing of Spastin delayed spindle microtubule severing (Figure 6F), although it had a negligible effect on the recruitment dynamics of CHMP2A-L-GFP (Figure S6A). Interestingly, the dynamics of microtubules disassembly in control and Spastin-silenced cells diverge at the time that coincides with the peak recruitment of CHMP2A-L-GFP (black curve in Figure 6F), supporting the notion that ESCRT-III recruitment triggers the severing of spindle microtubules by Spastin. Critically, the silencing of CC2D1B recapitulated the phenotypes observed in Spastin-depleted cells, as shown by the persistence of spindle microtubules at late stages of cell division (Figure 6E, bottom panels, and S6B, right panels; Video S15) and the delayed rate of spindle microtubule disassembly (Figure 6H). Importantly, these effects paralleled with the premature recruitment of CHMP2A-L-GFP to the reforming NE (Figure S6A). Of note, the above effects on microtubule disassembly were specific for the nuclear compartment as depletion of either Spastin or CC2D1B had no effect on the cytoplasmic signal of mCherry-tubulin (Figures 6G and 6I). Taken together, our results suggest that CC2D1B depletion mimics the effect of Spastin silencing on the severing of nuclear spindle microtubules, impairing the ability of the cells to accurately disassemble the mitotic spindle and proceed with the proper sealing of the NE.

Lastly, we reasoned that the persistence of spindle microtubules beyond cell division could give rise to cells bearing microtubules spanning the nuclear compartment during interphase, therefore providing a plausible model to explain the nuclear aberrations observed in CC2D1B-depleted cells. Supporting this notion, we detected transnuclear microtubules in HCT116 cells stably expressing mCherry-tubulin (Figure 7A; Video S16), observing that their frequency dramatically increased from 6% in the control cells to 17.39% in CC2D1B-depleted cells and 15.54% in Spastin-silenced cells (Figure 7B). 3D reconstruction of these cells revealed that tunnels containing microtubules pierce the nucleus, exerting a deforming force on its poles and inducing deep invaginations on its surface (Figures 7C and 7D; Video S16), therefore preventing the nucleus from reaching a typical round shape (Figure S7A). Taken together, our data suggest that the uncoordinated recruitment of ESCRT-III and Spastin to the reforming NE in CC2D1B-depleted cells has not only a local effect on the resealing of the NE but also more profound implications permanently modifying the morphology of the nucleus and therefore compromising its integrity and functionality.

DISCUSSION

We show here that CC2D1B organizes the timely recruitment of ESCRT-III and associated proteins to the sealing gaps of the

Figure 6. CC2D1B Silencing Impairs Spastin Activity

(A) Time-lapse analysis of HCT116 cells stably co-expressing GFP-Spastin M87 and H2B-mCherry. Cells were transfected with control, CC2D1B, or CHMP7 siRNAs. Scale bars, 10 μm.

(B) GFP-Spastin M87 fluorescence recruitment to the reforming NE in HCT116 cells transfected with control or CC2D1B siRNAs. T0 was set at the beginning of furrow ingression (FI). Curves indicate mean ± SEM. siControl n = 18; siCC2D1B n = 30.

(C) Representative WB corresponding to the cells shown in (A).

(D) Area under the curves corresponding to the recruitment of GFP-Spastin M87 fluorescence to the reforming NE shown in Figure 6B. Cells were transfected with control or CC2D1B siRNAs. Bar indicates median. p = 0.0008. Significance compared to the control was calculated using a two-tailed unpaired t test.

(E) Time-lapse analysis of HCT116 cells stably co-expressing mCherry-tubulin with CHMP2A-L-GFP and transfected with control, Spastin, or CC2D1B siRNAs. Scale bars, 10 μm.

(F–I) Quantification of nuclear or cytoplasmic tubulin fluorescence over time in HCT116 cells stably co-expressing mCherry-tubulin with CHMP2A-L-GFP and transfected with Spastin (F and G) or CC2D1B (H and I) siRNAs. T0 was set at the beginning of furrow ingression. siControl n = 10; siCC2D1B n = 8; siSpastin n = 9. (F and H) Nuclear tubulin fluorescence (lines indicate mean ± SEM) siSpastin p = 0.0158; siCC2D1B p = 0.0294. CHMP2A-L-GFP recruitment to the NE (curve indicates mean ± SEM) in control cells. (G and I) Cytoplasmic tubulin fluorescence (lines indicate mean ± SEM) siSpastin p = 0.0099; siCC2D1B p = 0.0007. Significance compared to the control was calculated at 600 s using a two-tailed unpaired t test.

See also Figure S6 and Video S15.

CellPress

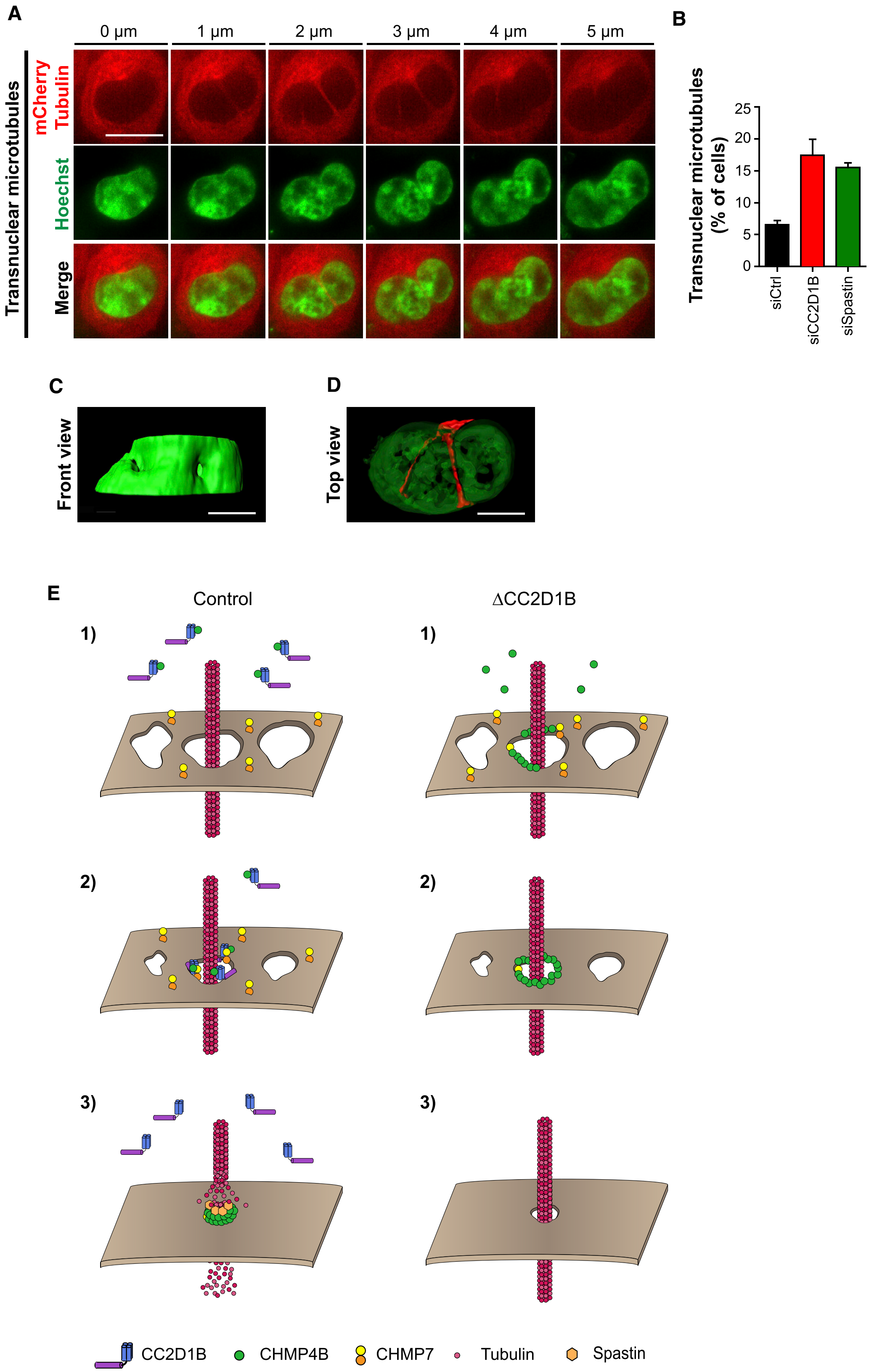

(legend on next page)

reforming NE. We demonstrate that CC2D1B silencing results in the premature and unregulated polymerization of ESCRT-III components at the NM during mitosis. As result of this dysregulation, the ability of Spastin to clear the nucleus from spindle microtubules is reduced, which provokes deleterious effects on nuclear morphology and integrity.

We propose a model (Figure 7E) whereby CC2D1B could play a double role in the regulation of CHMP4B activity. On one hand, the N-terminal DM14 domains of CC2D1B could mediate the binding and stabilization of the monomeric cytoplasmic form of CHMP4B, preventing then its premature or non-productive polymerization. On the other hand, the ability of CC2D1B to interact with membrane phospholipids through its C-terminal C2 domain could mediate the precise delivery of CHMP4B monomers to the gaps in the resealing NE, where they would be able to polymerize and form constriction competent ESCRT-III filaments. A role for CC2D1B as a scaffold protein able to position CHMP4B at the required location is supported by the solved structure of Lgd that shows a rigid connection between the C-terminal C2 domain and the helical domain that bridges to the N-terminal DM14 domains. Interestingly, an Lgd fragment consisting only of the third DM14 and the C2 domains is sufficient for Notch regulation (McMillan et al., 2017), corroborating that the C2 domain has an important role in CHMP4 positioning. Our model is also supported by previously published data indicating that members of the Lgd/CC2D1 family of proteins are able to interact with cell membranes *in vivo*, as they associate to the insoluble membrane fractions in cell fractioning experiments (Drusenheimer et al., 2015; Gallagher and Knoblich, 2006). Our PIP pull-down experiments suggest that Lgd/CC2D1 C2 domains specifically interact with PIns(4,5)P_2-enriched membranes, a result supported by the presence of PIns(4,5)P_2 foci on the reforming NE, and by the structural similarities with other C2 domains known to interact with PIns(4,5)P_2 (Guerrero-Valero et al., 2009; Guillén et al., 2013). The modeling of CC2D1B C2 domain binding to PIns(1,4,5)P_3 suggests a further level of regulation in the activity of Lgd/CC2D1 proteins via lipid modification (Dumas et al., 2010). It has been also described that Lgd binds preferentially to PIns(3)P, PIns(4)P and PIns(5)P in PIP strips experiments, although a weaker interaction with PIns(3,4)P_2, PIns(3,5)P_2, and PIns(4,5)P_2 was also detected (Gallagher and Knoblich, 2006). The apparent discrepancy with Gallagher et al. could be explained by the different methods used to analyze the weak interaction of Lgd with phospholipids.

Our data also suggest a regulatory interaction between CC2D1B and CHMP7. It has been shown that the third DM14 domain of CC2D1A and Lgd interacts preferentially with CHMP4B and shrub, respectively (Martinelli et al., 2012; McMillan et al., 2017), suggesting that other DM14 domains might be simultaneously available for CHMP7 interaction. Accordingly, our results indicate that the interaction of CC2D1B with CHMP4B and CHMP7 could be mediated by different DM14 domains. Thus, we hypothesize that the formation of a transient CHMP7-CC2D1B-CHMP4B complex at the gaps of the NE could be necessary for a fully productive polymerization of the ESCRT machinery. In this context, the cytoplasmic sequestration of CHMP4B by CC2D1B could keep CHMP7 away from the gaps in the NE until the membrane fenestrations reach a threshold size suitable for ESCRT polymerization. Conversely, in CC2D1B-depleted cells, CHMP7 and CHMP4B would be free to unrestrainedly interact with each other, triggering the premature and unregulated polymerization of ESCRT-III on the reforming NE. A role for CC2D1B in the regulation of the cytoplasmic activity of CHMP4B is supported by our data indicating that although the C2M mutant form of CC2D1B is not recruited to the resealing NE, its expression has no effect on the recruitment time of ESCRT-III.

The recruitment of Spastin to the growing ESCRT-III filaments depends on its ability to bind Ist1 and CHMP1B (Agromayor et al., 2009; Reid et al., 2005; Yang et al., 2008). Thus, we speculate that aberrant ESCRT-III polymers unable to efficiently bind Spastin may be formed at the NE in the absence of CC2D1B. Alternatively, the bundles of spindle microtubules may be immature for severing at the time Spastin is recruited in CC2D1B-depleted cells. Accordingly, it has been proposed that the spatial and temporal regulation of microtubule dynamics during nuclear reformation is key to ensure the normal functionality of the nucleus (Xue et al., 2013). We show here that as consequence of the defective severing of microtubules during the reassembly of the nuclear compartment, bundles of microtubules persist through interphase, forming deep nuclear invaginations that entirely transect the nucleus deforming its surface. As the

Figure 7. Persistent Transnuclear Microtubules Originate Deep Nuclear Invaginations in Interphase

(A) z stack series corresponding to an HCT116 cell stably co-expressing mCherry-tubulin with CHMP2A-L-GFP, transfected with CC2D1B siRNA, and incubated with Hoechst 33258 30 min before imaging to stain DNA. The signal corresponding to Hoechst 33258 is shown in green. The signal corresponding to CHMP2A-L-GFP is not shown. This cell was selected as an example of a nucleus showing transnuclear microtubule tunnels. Scale bar 10 μm.

(B) Frequency of transnuclear microtubule tunnels in HCT116 cells stably co-expressing mCherry-tubulin with CHMP2A-L-GFP and transfected with control, Spastin, or CC2D1B siRNAs. Mean ± SEM; siControl n = 1,083; siCC2D1B n = 850, p = 0.0145; siSpastin n = 1,075, p = 0.0007. Significance compared to the control was calculated using a two-tailed unpaired t test.

(C and D) 3D rendering of the cell shown in (A). (C) Front view corresponding to Hoechst 33258 channel. (D) Top view corresponding to Hoechst 33258 and mCherry-tubulin. 80% transparency was applied to Hoechst 33258 channel to facilitate the observation of transnuclear microtubule channels. Scale bars, 5 μm.

(E) Proposed model. Left panel: (1) CC2D1B binds to the monomeric cytoplasmic form of CHMP4B, hindering its association with CHMP7 and therefore preventing its premature recruitment to the NE. (2) By means of its ability to interact with CHMP7 and CHMP4B through its N-terminal DM14 domains and with membrane phospholipids through its C-terminal C2 domain, CC2D1B could function as a scaffold to position CHMP4B monomers close to the NM. Then, CC2D1B could mediate the organization of a transient CHMP7-CC2D1B-CHMP4B complex at the sealing gaps of the NE, facilitating the delivery of CHMP4B monomers to the growing ESCRT-III filament. (3) Spastin is recruited by the ESCRT-III to catalyze the severing of spindle microtubules, which facilitates the final sealing of the NE. Right panel: (1) in CC2D1B-silenced cells, CHMP4B monomers are free to associate to CHMP7 without restrictions, (2) triggering the premature polymerization of CHMP4B monomers on the NE. (3) These prematurely formed ESCRT filaments cannot be competent for Spastin recruitment and membrane constriction, which results in an impaired ability of the cells to sever spindle microtubules, leading to deleterious effects in nuclear integrity and functionality. See also Figure S7 and Video S16.

number of these transnuclear microtubule tunnels increases as consequence of the disruption of normal cell division, we speculate that the presence of these tunnels could have a deleterious effect on cell performance.

In summary, the evidence presented here reveals CC2D1B as a key player in the mitotic reformation of the NE, by licensing ESCRT-III polymerization after the ER-derived NM is fully deposited on chromatin disks. Our results also highlight the importance of ESCRT-associated proteins that negatively regulate ESCRT-III polymerization for the progress of cellular processes involving membrane remodeling and open the gate to the identification of similar mechanisms specifically implicated in other ESCRT-dependent processes besides the resealing of the NE.

STAR★METHODS

Detailed methods are provided in the online version of this paper and include the following:

- KEY RESOURCES TABLE
- CONTACT FOR REAGENT AND RESOURCE SHARING
- EXPERIMENTAL MODEL AND SUBJECT DETAILS
  - Cell Lines
- METHOD DETAILS
  - Retroviral Plasmids
  - Transfections
  - Generation of HCT116$^{\delta CC2D1B}$ Cell Line
  - GST Pulldowns
  - GFP Immunoprecipitations
  - Immunoblotting
  - Immunofluorescence
  - Spinning Disk Confocal Imaging
  - Super Resolution Microscopy Imaging
  - Wide Field Microscopy Imaging
  - Protein Expression and Purification
  - PIP Pull-Down Experiment
  - Sedimentation Experiments
  - Crystallization and Structure Solution of Lgd (550-816)
- QUANTIFICATION AND STATISTICAL ANALYSIS
- DATA AND SOFTWARE AVAILABILITY

SUPPLEMENTAL INFORMATION

Supplemental Information includes 7 figures, 1 table, 1 data file, and 16 videos and can be found with this article online at https://doi.org/10.1016/j.devcel.2018.11.012.

ACKNOWLEDGMENTS

J.M.-S. acknowledges funding for this project by Wellcome Trust (WT102871MA). We thank the UK NIHR Comprehensive BRC at KCL for an equipment grant and the Nikon Imaging Centre at KCL for technical support. W.W. acknowledges support by the Agence Nationale de la Recherche (ANR-08-BLAN-0271-01 and ANR-14-CE09-0003-01), the Institut Universitaire de France, a PhD fellowship from the Région Rhône-Alpes (Cluster 10 infectiologie) to N. Martinelli, the platforms of the Grenoble INSTRUCT-ERIC Center (ISBG; UMS 3518 CNRS-CEA-UJF-EMBL) supported by the French Infrastructure for Integrated Structural Biology Initiative FRISBI (ANR-10-INSB-05-02), and GRAL (ANR-10-LABX-49-01) within the Grenoble Partnership for Structural Biology (PSB) and the ESRF-EMBL Joint Structural Biology Group for access and support at the ESRF beam lines. We thank J. Marquez (EMBL) from the crystallization platform, Bettina Hartlieb for establishing the lgd expression clones, and Charles Sabin for help with data collection and analysis. J.G.C. is funded by a Wellcome Trust Research Career Development Fellowship (093603/Z/10/Z). We thank Dr. Tanya Shaw for the human dermal fibroblast, Dr. Axel Behrens for the mouse adult fibroblasts, Dr. Evan Reid for the pIRES Spastin M87 plasmid, and Dr. Monica Agromayor for critical reading of the manuscript.

AUTHOR CONTRIBUTIONS

L.N.V. and J.M.-S. conceived and designed the project. W.W. designed and supervised the structural work. L.N.V., M.A.C.-G., A.C., and I.M.P. generated tools and performed functional experiments. N. Martinelli, P.M., and N. Miguet performed structural and biochemical experiments. Y.O. and J.G.C. performed immunofluorescence experiments and helped with the experimental design. L.N.V., M.A.C.-G., N. Martinelli, A.C., P.M., Y.O., J.G.C., W.W., and J.M.-S. analyzed data. L.N.V., J.M.-S., and W.W. wrote the manuscript.

DECLARATION OF INTERESTS

The authors declare no competing interests.

Received: October 10, 2017
Revised: August 13, 2018
Accepted: November 2, 2018
Published: December 3, 2018

REFERENCES

Adams, P.D., Afonine, P.V., Bunkóczi, G., Chen, V.B., Davis, I.W., Echols, N., Headd, J.J., Hung, L.W., Kapral, G.J., Grosse-Kunstleve, R.W., et al. (2010). PHENIX: a comprehensive Python-based system for macromolecular structure solution. Acta Crystallogr. D Biol. Crystallogr. *66*, 213–221.

Agromayor, M., Carlton, J.G., Phelan, J.P., Matthews, D.R., Carlin, L.M., Ameer-Beg, S., Bowers, K., and Martin-Serrano, J. (2009). Essential role of hIST1 in cytokinesis. Mol. Biol. Cell *20*, 1374–1387.

Al-Tawashi, A., Jung, S.Y., Liu, D., Su, B., and Qin, J. (2012). Protein implicated in nonsyndromic mental retardation regulates protein kinase A (PKA) activity. J. Biol. Chem. *287*, 14644–14658.

Anderson, D.J., and Hetzer, M.W. (2007). Nuclear envelope formation by chromatin-mediated reorganization of the endoplasmic reticulum. Nat. Cell Biol. *9*, 1160–1166.

Asencio, C., Davidson, I.F., Santarella-Mellwig, R., Ly-Hartig, T.B.N., Mall, M., Wallenfang, M.R., Mattaj, I.W., and Gorjánácz, M. (2012). Coordination of kinase and phosphatase activities by Lem4 enables nuclear envelope reassembly during mitosis. Cell *150*, 122–135.

Bajorek, M., Schubert, H.L., McCullough, J., Langelier, C., Eckert, D.M., Stubblefield, W.M., Uter, N.T., Myszka, D.G., Hill, C.P., and Sundquist, W.I. (2009). Structural basis for ESCRT-III protein autoinhibition. Nat. Struct. Mol. Biol. *16*, 754–762.

Battye, T.G.G., Kontogiannis, L., Johnson, O., Powell, H.R., and Leslie, A.G.W. (2011). iMOSFLM: A new graphical interface for diffraction-image processing with MOSFLM. Acta Crystallogr. D Biol. Crystallogr. *67*, 271–281.

Biasini, M., Bienert, S., Waterhouse, A., Arnold, K., Studer, G., Schmidt, T., Kiefer, F., Cassarino, T.G., Bertoni, M., Bordoli, L., et al. (2014). SWISS-MODEL: modelling protein tertiary and quaternary structure using evolutionary information. Nucleic Acids Res. *42*, W252–W258.

Caballe, A., Wenzel, D.M., Agromayor, M., Alam, S.L., Skalicky, J.J., Kloc, M., Carlton, J.G., Labrador, L., Sundquist, W.I., and Martin-Serrano, J. (2015). ULK3 regulates cytokinetic abscission by phosphorylating ESCRT-III proteins. Elife *4*, e06547.

Carlton, J.G., and Martin-Serrano, J. (2007). Parallels between cytokinesis and retroviral budding: a role for the ESCRT machinery. Science *316*, 1908–1912.

CCP4. (1994). The CCP4 suite: programs for protein crystallography. Acta Crystallogr. D Biol. Crystallogr. *50*, 760–763.

Champion, L., Linder, M.I., and Kutay, U. (2017). Cellular reorganization during mitotic entry. Trends Cell Biol. *27*, 26–41.

Childress, J.L., Acar, M., Tao, C., and Halder, G. (2006). Lethal giant discs, a novel C2-domain protein, restricts Notch activation during endocytosis. Curr. Biol. *16*, 2228–2233.

Cowtan, K. (2006). The Buccaneer software for automated model building. 1. Tracing protein chains. Acta Crystallogr. D Biol. Crystallogr. *62*, 1002–1011.

Cowtan, K., Emsley, P., and Wilson, K.S. (2011). From crystal to structure with CCP4. Acta Crystallogr. D Biol. Crystallogr. *67*, 233–234.

Denais, C.M., Gilbert, R.M., Isermann, P., McGregor, A.L., Te Lindert, M., Weigelin, B., Davidson, P.M., Friedl, P., Wolf, K., and Lammerding, J. (2016). Nuclear envelope rupture and repair during cancer cell migration. Science *352*, 353–358.

Kim, D.I., Jensen, S.C., Noble, K.A., Kc, B., Roux, K.H., Motamedchaboki, K., and Roux, K.J. (2016). An improved smaller biotin ligase for BioID proximity labeling. Mol. Biol. Cell *27*, 1188–1196.

Drusenheimer, N., Migdal, B., Jäckel, S., Tveriakhina, L., Scheider, K., Schulz, K., Gröper, J., Köhrer, K., and Klein, T. (2015). The mammalian orthologs of Drosophila Lgd, CC2D1A and CC2D1B, function in the endocytic pathway, but their individual loss of function does not affect Notch signalling. PLoS Genet. *11*, e1005749.

Dumas, F., Byrne, R.D., Vincent, B., Hobday, T.M.C., Poccia, D.L., and Larijani, B. (2010). Spatial regulation of membrane fusion controlled by modification of phosphoinositides. PLoS One *5*, e12208.

Emsley, P., Lohkamp, B., Scott, W.G., and Cowtan, K. (2010). Features and development of coot. Acta Crystallogr. D Biol. Crystallogr. *66*, 486–501.

Essen, L.O., Perisic, O., Cheung, R., Katan, M., and Williams, R.L. (1996). Crystal structure of a mammalian phosphoinositide-specific phospholipase C delta. Nature *380*, 595–602.

Evans, P. (2006). Scaling and assessment of data quality. Acta Crystallogr. D Biol. Crystallogr. *62*, 72–82.

Gallagher, C.M., and Knoblich, J.A. (2006). The conserved C2 domain protein lethal (2) giant discs regulates protein trafficking in Drosophila. Dev. Cell *11*, 641–653.

Gouet, P., Courcelle, E., Stuart, D.I., and Métoz, F. (1999). ESPript: analysis of multiple sequence alignments in PostScript. Bioinformatics *15*, 305–308.

Grosdidier, A., Zoete, V., and Michielin, O. (2011). SwissDock, a protein-small molecule docking web service based on EADock DSS. Nucleic Acids Res. *39*, W270–W277.

Gu, M., LaJoie, D., Chen, O.S., von Appen, A., Ladinsky, M.S., Redd, M.J., Nikolova, L., Bjorkman, P.J., Sundquist, W.I., Ullman, K.S., et al. (2017). LEM2 recruits CHMP7 for ESCRT-mediated nuclear envelope closure in fission yeast and human cells. Proc. Natl. Acad. Sci. USA *114*, E2166–E2175.

Guerrero-Valero, M., Ferrer-Orta, C., Querol-Audí, J., Marin-Vicente, C., Fita, I., Gómez-Fernández, J.C., Verdaguer, N., and Corbalán-García, S. (2009). Structural and mechanistic insights into the association of PKCalpha-C2 domain to PtdIns(4,5)P2. Proc. Natl. Acad. Sci. USA *106*, 6603–6607.

Guillén, J., Ferrer-Orta, C., Buxaderas, M., Pérez-Sánchez, D., Guerrero-Valero, M., Luengo-Gil, G., Pous, J., Guerra, P., Gómez-Fernández, J.C., Verdaguer, N., et al. (2013). Structural insights into the Ca2+ and PI(4,5)P2 binding modes of the C2 domains of rabphilin 3A and synaptotagmin 1. Proc. Natl. Acad. Sci. USA *110*, 20503–20508.

Hao, Q. (2004). ABS: a program to determine absolute configuration and evaluate anomalous scatterer substructure. J. Appl. Crystallogr. *37*, 498–499.

Horii, M., Shibata, H., Kobayashi, R., Katoh, K., Yorikawa, C., Yasuda, J., and Maki, M. (2006). CHMP7, a novel ESCRT-III-related protein, associates with CHMP4b and functions in the endosomal sorting pathway. Biochem. J. *400*, 23–32.

Jaekel, R., and Klein, T. (2006). The Drosophila Notch inhibitor and tumor suppressor gene lethal (2) giant discs encodes a conserved regulator of endosomal trafficking. Dev. Cell *11*, 655–669.

Lu, L., Ladinsky, M.S., and Kirchhausen, T. (2011). Formation of the postmitotic nuclear envelope from extended ER cisternae precedes nuclear pore assembly. J. Cell Biol. *194*, 425–440.

Martinelli, N., Hartlieb, B., Usami, Y., Sabin, C., Dordor, A., Miguet, N., Avilov, S.V., Ribeiro, E.A., Göttlinger, H., and Weissenhorn, W. (2012). CC2D1A is a regulator of ESCRT-III CHMP4B. J. Mol. Biol. *419*, 75–88.

Martin-Serrano, J., Yarovoy, A., Perez-, D., Bieniasz, P.D., Severt, L., and Delorenzo, R.J. (2003). Divergent retroviral late-budding domains recruit vacuolar protein sorting factors by using alternative adaptor proteins. PNAS *100*, 10141–10143.

McCoy, A.J., Grosse-Kunstleve, R.W., Adams, P.D., Winn, M.D., Storoni, L.C., and Read, R.J. (2007). Phaser crystallographic software. J. Appl. Crystallogr. *40*, 658–674.

McMillan, B.J., Tibbe, C., Drabek, A.A., Seegar, T.C.M., Blacklow, S.C., and Klein, T. (2017). Structural basis for regulation of ESCRT-III complexes by Lgd. Cell Rep. *19*, 1750–1757.

Morris, R.J., Zwart, P.H., Cohen, S., Fernandez, F.J., Kakaris, M., Kirillova, O., Vonrhein, C., Perrakis, A., and Lamzin, V.S. (2004). Breaking good resolutions with ARP/wARP. J. Synchrotron Radiat. *11*, 56–59.

Murshudov, G.N., Vagin, A.A., and Dodson, E.J. (1997). Refinement of macromolecular structures by the maximum-likelihood method. Acta Crystallogr. D Biol. Crystallogr. *53*, 240–255.

Muzioł, T., Pineda-Molina, E., Ravelli, R.B., Zamborlini, A., Usami, Y., Göttlinger, H., and Weissenhorn, W. (2006). Structural basis for budding by the ESCRT-III factor CHMP3. Dev. Cell *10*, 821–830.

Olmos, Y., and Carlton, J.G. (2016). The ESCRT machinery: new roles at new holes. Curr. Opin. Cell Biol. *38*, 1–11.

Olmos, Y., Hodgson, L., Mantell, J., Verkade, P., and Carlton, J.G. (2015). ESCRT-III controls nuclear envelope reformation. Nature *522*, 236–239.

Olmos, Y., Perdrix-Rosell, A., and Carlton, J.G. (2016). Membrane binding by CHMP7 coordinates ESCRT-III-dependent nuclear envelope reformation. Curr. Biol. *26*, 2635–2641.

Panjikar, S., Parthasarathy, V., Lamzin, V.S., Weiss, M.S., and Tucker, P.A. (2005). Auto-Rickshaw: an automated crystal structure determination platform as an efficient tool for the validation of an x-ray diffraction experiment. Acta Crystallogr. D Biol. Crystallogr. *61*, 449–457.

Pires, R., Hartlieb, B., Signor, L., Schoehn, G., Lata, S., Roessle, M., Moriscot, C., Popov, S., Hinz, A., Jamin, M., et al. (2009). A crescent-shaped ALIX dimer targets ESCRT-III CHMP4 filaments. Structure *17*, 843–856.

Raab, M., Gentili, M., de Belly, H., Thiam, H.R., Vargas, P., Jimenez, A.J., Lautenschlaeger, F., Voituriez, R., Lennon-Duménil, A.M., Manel, N., et al. (2016). ESCRT III repairs nuclear envelope ruptures during cell migration to limit DNA damage and cell death. Science *352*, 359–362.

Reid, E., Connell, J., Edwards, T.L., Duley, S., Brown, S.E., and Sanderson, C.M. (2005). The hereditary spastic paraplegia protein spastin interacts with the ESCRT-III complex-associated endosomal protein CHMP1B. Hum. Mol. Genet. *14*, 19–38.

Schellhaus, A.K., De Magistris, P., and Antonin, W. (2016). Nuclear reformation at the end of mitosis. J. Mol. Biol. *428*, 1962–1985.

Schindelin, J., Arganda-Carreras, I., Frise, E., Kaynig, V., Longair, M., Pietzsch, T., Preibisch, S., Rueden, C., Saalfeld, S., Schmid, B., et al. (2012). Fiji: an open-source platform for biological-image analysis. Nat. Methods *9*, 676–682.

Schneider, T.R., and Sheldrick, G.M. (2002). Substructure solution with SHELXD. Acta Crystallogr. D Biol. Crystallogr. *58*, 1772–1779.

Scourfield, E.J., and Martin-Serrano, J. (2017). Growing functions of the ESCRT machinery in cell biology and viral replication. Biochem. Soc. Trans. *45*, 613–634.

Sheldrick, G.M. (2002). Macromolecular phasing with SHELXE. Z. Kristallogr. Cryst. Mater. *217*, 644–650.

Troost, T., Jaeckel, S., Ohlenhard, N., and Klein, T. (2012). The tumour suppressor lethal (2) giant discs is required for the function of the ESCRT-III component Shrub/CHMP4. J. Cell Sci. *125*, 763–776.

Usami, Y., Popov, S., Weiss, E.R., Vriesema-Magnuson, C., Calistri, A., and Göttlinger, H.G. (2012). Regulation of CHMP4/ESCRT-III function in human immunodeficiency virus type 1 budding by CC2D1A. J. Virol. *86*, 3746–3756.

Vantourout, P., Laing, A., Woodward, M.J., Zlatareva, I., Apolonia, L., and Jones, A.W. (2018). Heteromeric interactions regulate butyrophilin (BTN) and BTN-like molecules governing $\gamma\delta$ T cell biology. PNAS *115*, 1039–1044.

Vietri, M., Schink, K.O., Campsteijn, C., Wegner, C.S., Schultz, S.W., Christ, L., Thoresen, S.B., Brech, A., Raiborg, C., and Stenmark, H. (2015). Spastin and ESCRT-III coordinate mitotic spindle disassembly and nuclear envelope sealing. Nature *522*, 231–235.

Vietri, M., Stenmark, H., and Campsteijn, C. (2016). Closing a gap in the nuclear envelope. Curr. Opin. Cell Biol. *40*, 90–97.

Webster, B.M., and Lusk, C.P. (2016). Border safety: quality control at the nuclear envelope. Trends Cell Biol. *26*, 29–39.

Xiao, J., Chen, X.W., Davies, B.A., Saltiel, A.R., Katzmann, D.J., and Xu, Z. (2009). Structural basis of Ist1 function and Ist1-Did2 interaction in the multivesicular body pathway and cytokinesis. Mol. Biol. Cell *20*, 3514–3524.

Xue, J.Z., Woo, E.M., Postow, L., Chait, B.T., and Funabiki, H. (2013). Chromatin-bound Xenopus Dppa2 shapes the nucleus by locally inhibiting microtubule assembly. Dev. Cell *27*, 47–59.

Yang, D., Rismanchi, N., Renvoisé, B., Lippincott-Schwartz, J., Blackstone, C., and Hurley, J.H. (2008). Structural basis for midbody targeting of spastin by the ESCRT-III protein CHMP1B. Nat. Struct. Mol. Biol. *15*, 1278–1286.

Zamarbide, M., Oaks, A.W., Pond, H.L., Adelman, J.S., and Manzini, M.C. (2018). Loss of the intellectual disability and autism gene Cc2d1a and its homolog Cc2d1b differentially affect spatial memory, anxiety, and hyperactivity. Front. Genet. *9*, 65.

STAR★METHODS

KEY RESOURCES TABLE

REAGENT or RESOURCE	SOURCE	IDENTIFIER
Antibodies		
Rabbit polyclonal anti-CC2D1B (H-258)	Santa Cruz Biotechnology	Cat# sc-134634; RRID: AB_10611611
Rabbit polyclonal anti-CHMP7	Proteintech	Cat #16424-1-AP; RRID: AB_2079500
Mouse monoclonal anti-Spastin (Sp 3G11/1)	Santa Cruz Biotechnology	Cat# sc-53443; RRID: AB_785771
Mouse monoclonal anti-GFP (clones 7.1 and 13.1)	Roche	Cat# 11814460001; RRID: AB_390913
Rabbit polyclonal anti-HSP90α/β (H-114)	Santa Cruz Biotechnology	Cat# sc-7947; RRID: AB_2121235
Mouse monoclonal anti-HSP90α/β (F-8)	Santa Cruz Biotechnology	Cat# sc-13119; RRID: AB_675659
Mouse monoclonal anti-GST (B14)	Santa Cruz Biotechnology	Cat# sc-138; RRID: AB_627677
Rabbit polyclonal anti-HA	Antibodies Online	Cat# ABIN100176; RRID: AB_10779560
Rabbit polyclonal anti-Lamin B1	Abcam	Cat# ab16048; RRID: AB_443298)
Chicken polyclonal anti-GFP	Abcam	Cat# ab13970; RRID: AB_300798
Mouse monoclonal anti-alpha Tubulin	Abcam	Cat# ab7291; RRID: AB_2241126
Rabbit polyclonal anti-CHMP2A	Proteintech	Cat# 10477-1-AP; RRID: AB_2079470
Mouse monoclonal anti PtdIns(4,5)P_2	Echelon	Cat# Z-P045 RRID: AB_427225
Secondary donkey anti-rabbit Alexa Fluor 555	ThermoFisher Scientific	Cat# A31572; RRID: AB_162543
Secondary goat anti-chicken Alexa Fluor 488	ThermoFisher Scientific	Cat# A11039; RRID: AB_142924
Secondary goat anti-rabbit Alexa Fluor 488	ThermoFisher Scientific	Cat# A11008; RRID: AB_143165
Secondary donkey anti-mouse Alexa Fluor 594	ThermoFisher Scientific	Cat# A21203; AB_141633
Secondary goat anti-mouse (DyLight™ 680 Conjugate)	Cell Signaling Technology	Cat# 5470S; RRID: AB_10696895
Secondary goat anti-rabbit (DyLight™ 800 Conjugate)	Cell Signaling Technology	Cat# 5151; RRID: AB_10697505
Bacterial and Virus Strains		
BL21	New England Biolabs	C2530H
Chemicals, Peptides, and Recombinant Proteins		
cOmplete™ Protease Inhibitor Cocktail	Sigma-Aldrich	Cat#000000011697498001
Lipofectamine RNAiMax	Life Technologies	Cat#13778075
Polyethylenimine (PEI)	Polysciences	Cat# 23966-1
DharmaFECT-1	Dharmacon	Cat#T-2001
Glutathione Sepharose 4B beads	GE Healthcare Life Sciences	Cat# 17075601
GFP-Trap M	ChromoTek	Cat#GTM-20
Prolong Diamond Antifade Mountant	ThermoFisher Scientific	Cat#P36965
Hoechst 33258	Sigma-Aldrich	Cat#861405
PIP beads	Echelon	Cat#P-B00S
Ni-NTA Agarose	Qiagen	Cat#30210
PEG 3500 Mme	Sigma	Cat#701750
Deposited Data		
Lgd (residues 550-816) structure	This paper	PDB: 6EI6

(*Continued on next page*)

Continued

REAGENT or RESOURCE	SOURCE	IDENTIFIER
Experimental Models: Cell Lines		
HCT116	ATCC	Cat# CCL-247; RRID: CVCL_0291
Hela	ATCC	Cat# CRM-CCL-2, RRID: CVCL_0030
hTert-RPE-1	ATCC	Cat# CRL-4000; RRID: CVCL_4388
Human dermal fibroblasts (HDF)	Tanya Shaw laboratory (KCL)	N/A
Mouse adult fibroblasts (MAF)	Axel Behrens laboratory (Francis Crick Institute)	N/A
Oligonucleotides		
CC2D1B siRNA 5'-GAUCAACUCUUCAAAC UAAUU-3'	Dharmacon	N/A
CHMP7 siRNA 5'-GGGAGAAGAUUGUGAAGU UUUUU-3'	Dharmacon	N/A
VPS4A siRNA 5'- CCGAGAAGCUGAAGGAUUA-3'	Dharmacon	N/A
VPS4B siRNA 5'- CCAAAGAAGCACUGAAAGA-3'	Dharmacon	N/A
Spastin siRNA	Qiagen	Cat# SI02781219
Mouse CC2D1B siRNA	Dharmacon	Cat# L-056827-00-0005
Recombinant DNA		
pNG72 GFP-CC2D1B Wt	This paper	N/A
pNG72 GFP-CC2D1B C2M	This paper	N/A
pNG72 GFP-Spastin M87	This paper	N/A
pNG72 CHMP4B-Linker-GFP	This paper	N/A
pNG72 CHMP2A-Linker-GFP	This paper	N/A
pNG72 GFP-NLS	This paper	N/A
pCMS28 mCherry-NLS	This paper	N/A
pCMS28 YFP-Sec61β	This paper	N/A
pCMS28 mCherry-Sec61β	This paper	N/A
pCMS28 mCherry-Lap2β (residues 244-454)	This paper	N/A
pCMS28 H2B-mCherry	This paper	N/A
pCMS28 GFP-CHMP7	Olmos et al., 2016	N/A
pCMS28 mCherry-Tubulin	Agromayor et al., 2009	N/A
pCMS28 mCherry-Emerin	This paper	N/A
pCMS28 CHMP2A-Linker-mCherry	This paper	N/A
pCAG GST-CC2D1B	This paper	N/A
pCAG GST-CC2D1B (residues 528-594)	This paper	N/A
pCAG GST-CHMP4B	This paper	N/A
pCAG GST-CHMP7	This paper	N/A
pCR3.1 HA-CHMP7	This paper	N/A
pCR3.1 HA-CHMP4B	Martin-Serrano et al., 2003	N/A
pCR3.1 HA-CHMP2A	Martin-Serrano et al., 2003	N/A
pCR3.1 HA-CHMP7 Nt	This paper	N/A
pCR3.1 HA-CHMP7 Ct	This paper	N/A
pCR3.1 YFP-CC2D1B	This paper	N/A
pCR3.1 HA-CC2D1B (residues 162-493)	This paper	N/A
pCR3.1 HA-CC2D1B (residues 162-594)	This paper	N/A
pLGC2	Vantourout et al., 2018	N/A
pProEx-Htb Lgd (residues 550-816)	This paper	N/A
pProEx-Htb Lgd (residues358-816)	This paper	N/A
pProEx-Htb CC2D1B (residues317-558)	This paper	N/A

(*Continued on next page*)

Continued

REAGENT or RESOURCE	SOURCE	IDENTIFIER
Software and Algorithms		
Image J	https://fiji.sc/	N/A
Prism	GrahpPad Software	N/A
Imaris	Bitplane	N/A
AutoQuant X3	Media Cybernetics	N/A
PyMOL	www.pymol.org	N/A

CONTACT FOR REAGENT AND RESOURCE SHARING

Further information and requests for resources and reagents should be directed to and will be fulfilled by the Lead Contact, Juan Martin-Serrano (juan.martin_serrano@kcl.ac.uk).

EXPERIMENTAL MODEL AND SUBJECT DETAILS

Cell Lines

Male HCT116 cells, female Hela cells, 293T cells of indeterminate sex and MAF cells derived from female mice were cultured in Dulbecco's Modified Eagle Medium (DMEM) containing 10% fetal calf serum (FCS) and gentamycin 20 μg/ml. hTert-RPE-1 cells were cultured in Dulbecco's Modified Eagle Medium (DMEM) containing 10% fetal calf serum (FCS), NEAA, hygromycin B 10 μg/ml and gentamycin 20 μg/ml. HDF were cultured in Dulbecco's Modified Eagle Medium (DMEM) containing 10% fetal calf serum (FCS) and penicillin-streptomycin (100 U/ml). Stable cells lines were generated using MLV-based retroviruses as described previously (Carlton and Martin-Serrano, 2007) and selected using puromycin (120 ng/ml for HCT116 or 200 ng/ml for Hela) or G418 (250 μg/ml for HCT116 or 500 μg/ml for Hela).

METHOD DETAILS

Retroviral Plasmids

Modified versions of pCMS28 (Carlton and Martin-Serrano, 2007) or pNG72 (Caballe et al., 2015) plasmids were used as retroviral packaging vectors. pCMS28 mCherry-Tubulin and pCMS28 GFP-CHMP7 constructs were previously described (Agromayor et al., 2009; Olmos et al., 2016). CC2D1B Wt, CC2D1B-C2M and Spastin M87 were cloned as N-terminal GFP fusions into pNG72-GFP. CHMP2A-Linker and CHMP4B-Linker were generated by gene synthesis (GeneArt, ThermoFisher) and cloned as C-terminal GFP fusions into pNG72-GFP. The SV40 Large T antigen nuclear localization signal was cloned as N-terminal GFP fusions into pNG72-GFP or as N-terminal mCherry fusion into pCMS28. Sec61β was cloned as N-terminal YFP or mCherry fusions into pCMS28. Lap2β residues 244-454 was cloned as a N-terminal mCherry fusion in pCMS28. Emerin was cloned as a N-terminal mCherry fusion in pCMS28. H2B-mCherry was cloned as a C-terminal mCherry fusion in pCMS28.

Transfections

For retroviral production or GST pulldowns, 293T cells were transfected using Polyethylenimine (PEI; Polysciences, Germany).

For siRNA assays, HCT116, HDF and MAF cells were transfected using Lipofectamine RNAiMax (Invitrogen, Life Technologies) and Hela cells were transfected using Dharmafect-1 (Dharmacon RNA Technologies) according to manufacturer's instructions. Custom siRNA against CC2D1B (5'-GAUCAACUCUUCAAACUAAUU-3'), siCHMP7 (5'-GGGAGAAGAUUGUGAAGUUUUUU-3'), siVPS4A (5'- CCGAGAAGCUGAAGGAUUA-3') and siVPS4B (5'- CCAAAGAAGCACUGAAAGA-3') were purchased from Dharmacon. siRNA against Spastin (Hs_SPAST_1, catalog no. SI02781219) was purchased from Qiagen. siRNA against mouse CC2D1B (ON-TARGETplus Mouse CC2D1B siRNA – SMARTpool, catalog no. L-056827-00-0005) was purchased from Dharmacon.

Generation of HCT116$^{\delta CC2D1B}$ Cell Line

A pair of guide RNA targeting the adjacent regions to the CC2D1B locus were designed using the Zhang Lab website (http://crispr.mit.edu) and cloned into a pLG2C plasmid expressing GFP and Cas9 (kindly gifted by Dr. Pierre Vantourout, KCL). The sequences for the guide oligos were 5′- CAA CAG TGC TAC CCG TGG TA GTT TT-3′ and 5′-AAG ACC TAC CAT GCT GGG TA GTT TT-3′. HCT116 cells were co-transfected with both guides using Lipofectamine 3000 (Invitrogen, Life Technologies). 48 hours later single cells expressing GFP were FACS sorted. HCT116 clones tested for CC2D1B expression by western blot.

GST Pulldowns

293T cells from 6-well plates were co-transfected with the corresponding pCAG-GST- and pCR3.1 HA-tagged constructs. 48 hours later, the cells were lysed at 4°C in 1 ml of lysis buffer containing 50 mM Tris, pH 7.4, 150 mM NaCl, 5 mM EDTA, 5% glycerol,

1% Triton X-100 and a protease inhibitor cocktail (complete mini-EDTA free, Sigma). The lysates were sonicated and centrifugated at 15.000 rpm for 10 minutes and the supernatant was incubated with glutathione–sepharose beads (Amersham Biosciences) for 3 hours at 4°C and washed three times with wash buffer containing 50 mM Tris, pH 7.4, 150 mM NaCl, 5 mM EDTA, 5% glycerol, and 0.1% Triton X-100. Bound proteins were eluted in 100 μl of Laemmli buffer, boiled and analyzed by western blot.

GFP Immunoprecipitations

HCT116 cell lines stably expressing CHMP4B-L-GFP or CHMP2A-L-GFP fusions were lysed at 4°C in 1 ml of lysis buffer as described above for GST pulldowns. Clarified lysates were incubated with anti-GFP coupled magnetic microparticles (GFP-Trap, ChromoTek) for 2 hours and the microparticles were washed four times with wash buffer. Bound proteins were eluted in 100 μl of Laemmli buffer, boiled and analyzed by western blot.

Immunoblotting

Samples were resolved by SDS-PAGE and proteins transferred to Nitrocellulose membranes. The corresponding primary and DyLight- or HRP-conjugated secondary antibodies were diluted in 1% milk. The membranes were scanned using a Li-Cor Odyssey Infrared scanner and software system (Li-Cor Biosciences). The primary antibodies used were: rabbit CC2D1B (H-258, Santa Cruz Biotechnology), rabbit CHMP7 (16424-1-AP, Proteintech), mouse Spastin (Sp 3G11/1, Santa Cruz Biotechnology), mouse GFP clones 7.1 and 13.1 (11814460001, Roche), rabbit Hsp90 (H-114, Santa Cruz Biotechnology), mouse Hsp90 (F-8, Santa Cruz Biotechnology), mouse GST (B-14, Santa Cruz Biotechnology), rabbit HA (ABIN100176, Antibodies Online). The secondary antibodies used were: goat anti-mouse DyLight 680-conjugated (Cell Signaling), goat anti-rabbit DyLight 800-conjugated (Cell Signaling), goat anti-mouse HRP-conjugated (Cell Signaling), goat anti-rabbit HRP-conjugated (Cell Signaling). The raw western blots corresponding to the insets shown in the main and supplemental figures are shown in Data S1.

Immunofluorescence

Cells were grown on coverslips and fixed for 10 minutes with 4% PFA at 4°C or for 20 minutes with methanol at -20°C. Cells were blocked with 1% FCS in PBS, stained with the indicated primary antibodies, washed and stained with the corresponding Alexa-conjugated secondary antibodies (ThermoFisher Scientific). DNA was stained with Hoechst 33258. Coverslips were mounted in Prolong Diamond Antifade Mountant (ThermoFisher Scientific). The primary antibodies used were: rabbit Lamin B1 (ab16048, Abcam), chicken GFP (ab13970, Abcam), mouse tubulin DM1A (ab7291, Abcam), rabbit CHMP2A (10477-1-AP Proteintech), mouse PtdIns(4,5)P_2 (Z-P045, Echelon). Fixed cells were imaged on a Nikon Ti-Eclipse wide-field inverted microscope equipped with a CoolSnap HQ2 CCD camera (Photometrics, Tucson, AZ).

Spinning Disk Confocal Imaging

ESCRT-Related Proteins Recruitment to the NE

Cells stably-expressing the corresponding GFP- or mCherry-tagged proteins were seeded on 24-wells glass bottom plates (Cellvis) and transfected with the corresponding siRNA. Cells were imaged 48 hours later using 100x oil-immersion objective equipped Nikon Eclipse Ti-E inverted CSU-X1 spinning disk confocal microscope with attached environmental chamber. Images were acquired every 15 seconds for HCT116 cells or every 10 seconds for Hela cells using an Andor Neo sCMOS camera using 2x2 binning. The quantification of the time-lapse images was performed using Fiji (Schindelin et al., 2012). Briefly, GFP fluorescence around chromatin was selected by manually adjusting the threshold of the green channel. Then a binary mask was created and the total intensity (mean intensity x mask area) of GFP-tagged proteins was quantified for every time point. The curve corresponding to the recruitment of GFP-tagged proteins was represented as mean ± SEM and the beginning of the furrow ingression was set as t0. The area under the curves was quantified using Prism (GraphPad Software).

To compare the timing of GFP-CC2D1B versus CHMP4B-L-GFP recruitment, the first (Ti) and the last (Tf) frames showing GFP accumulation in the perinuclear area were manually scored in every movie and the data were represented as 5-95 percentile boxes using Prism (GraphPad Software).

Nuclear Membrane Deposition

Hela cells stably co-expressing YFP-Sec61β and H2B-mCherry were seeded on 24-wells glass bottom plates (Cellvis), transfected with the corresponding siRNA and imaged in a Nikon Eclipse Ti-E inverted CSU-X1 spinning disk microscope using the same conditions as described above. The fluorescence from the reforming NE was measured with Fiji (Schindelin et al., 2012). A binary mask ($Mask^o$) from H2B-mCherry channel was automatically obtained, and this mask was dilated to include the NE ($Mask^d$). Both masks were exported to the YFP-Sec61β channel and the integrated density for YFP signal was measured over time. The integrated density of YFP in $Mask^o$ was sustracted from the integrated density in $Mask^d$ and the resulting value was plotted as a marker of NM deposition.

Spindle Microtubules Depolymerisation

HCT116 cells stably co-expressing mCherry-Tubulin and CHMP2A-L-GFP were seeded on 24-wells glass bottom plates (Cellvis), transfected with the corresponding siRNA and imaged in a Nikon Eclipse Ti-E inverted CSU-X1 spinning disk microscope using the same conditions as described above. mCherry-Tubulin fluorescence was quantified in Fiji (Schindelin et al., 2012) by drawing a line through the nuclear spindle microtubules or the cytoplasm in every time-lapse image. The t0 was set at the beginning of furrow ingression and fluorescence values were normalized to t0 and represented as mean ± SEM.

Analysis of Transnuclear Microtubules Tunnels

Cells stably co-expressing mCherry-Tubulin and CHMP2A-L-GFP were seeded on 24-wells glass bottom plates (Cellvis) and transfected with the corresponding siRNA. Cells were incubated with Hoechst 33258 to stain the DNA 30 minutes before imaging. Z-stacks of images separated by 100 nm were acquired using a 100x oil-immersion objective equipped Nikon Eclipse Ti-E inverted CSU-X1 spinning disk confocal microscope with attached environmental chamber and connected to Andor Neo sCMOS camera without binning. The Z-stacks were used to quantify the percentage of cells showing transnuclear microtubule tunnels.

For 3D reconstruction of nuclei, cells were treated and imaged as above, Z-stacks were deconvoluted using AutoQuant X3 Deconvolution Software (Media Cybernetics) and 3D renderizations were perfomed using Imaris software (Bitplane).

Super Resolution Microscopy Imaging

Cells stably co-expressing CHMP4B-linker-GFP and mCherry-Emerin were seeded on 24-wells glass bottom plates (Cellvis) and transfected with the corresponding siRNA. Cells were imaged 48 hours later using 100x oil-immersion objective equipped Nikon Eclipse Ti-2 Inverted spinning disk confocal microscope with a VT-iSIM scan head (VisiTech International) and an environmental chamber. Images were acquired every 15 seconds using a Hamamatsu Orca-Flash4.0 camera. The images were deconvolved using Nikon software.

To quantify the percentage of NE closure at CHMP4B Ti, we selected the first frame of the time-lapse acquisition showing CHMP4B-L-GFP recruitment to the resealing NE (CHMP4B Ti). Next, we used Nikon Software to calculate the nuclear perimeter (NP) by manually drawing a line around the nucleus. Then, we quantified the extent of NE reformation (NER) by manually drawing a line on the mCherry-Emerin signal. Finally, we calculated the ratio NER/NP for every image and refer to this ratio as percentage of NE closure at CHMP4B Ti.

Wide Field Microscopy Imaging

For the analysis of nuclear GFP- or mCherry-NLS fluorescence recovery, cells stably expressing GFP-NLS and H2B-mCherry were transfected twice with the corresponding siRNA and imaged 6 hours after the second transfection. Images were acquired every 5 minutes using a 40x oil-immersion objective equipped Nikon Ti-Eclipse wide-field inverted microscope with attached environmental chamber. Z-Stacks of 3 slices with a separation of 0.9 μm were acquired using an ORCA-Flash 4.0 camera (Hamamatsu). For the quantification of the nuclear to cytoplasmic ratio of GFP-NLS, a square ROI was manually drawn and nuclear and cytoplasmic GFP fluorescence, as well as background fluorescence, were quantified for every time point using Fiji (Schindelin et al., 2012). For nuclear expansion rate quantification, the area corresponding to the H2B-mCherry channel was scored in every frame using a thresholding method in Fiji. For the analysis of nuclear mCherry-NLS fluorescence recovery the same method was followed but cells stably expressing mCherry-NLS were incubated with Hoechst 33258 to stain the DNA 30 minutes before imaging.

For the quantification of anaphase onset and furrow ingression, cells stably expressing GFP-NLS and H2B-mCherry were transfected twice with the corresponding siRNA and imaged 6 hours after the second transfection. Images were acquired every 1 minute using a 40x oil-immersion objective equipped Nikon Ti-Eclipse wide-field inverted microscope with attached environmental chamber and connected to an ORCA-Flash 4.0 camera (Hamamatsu).

The quantification of midbodies abscission time in HCT116 cells stably co-expressing mCherry-Tubulin with CHMP2A-L-GFP or CHMP4B-L-GFP was performed as previously described (Caballe et al., 2015).

Protein Expression and Purification

cDNA encoding Lgd (residues 550-816), Lgd (residues 358-816) and CC2D1B (residues 317-558) were cloned into expression vector pProEx-Htb (Invitrogen) and protein expression was performed in BL21 *E. coli* cells. Cells were grown at 37°C to an OD of 0.6 and protein expression was induced with 1mM IPTG for 3 h.

Bacteria expressing Lgd (residues 550-816) were lysed by sonication in buffer A (50 mM Tris pH 7.5, 150 mM NaCl, 5 mM β-mercaptoethanol, 20 mM imidazole) including EDTA-free protease inhibitors (Sigma), pelleted by centrifugation and the soluble fraction was loaded onto a Ni-NTA (Qiagen) resin. The column was washed with buffer A supplemented with 1 M NaCl and subsequently with buffer B (50 mM Tris pH 7.4, 150 mM NaCl, 50 mM imidazole). The protein was eluted in buffer C (50 mM Tris pH 7.4, 150 mM NaCl, 250 mM imidazole). The His-tag was removed with TEV protease at a ratio of 2 μg for 1 mg of protein overnight at 4°C. The processed protein was dialyzed against buffer A, loaded on a Ni-NTA resin and the flow-through collected. Lgd (residues 550-816) was further purified on a Superdex 75 column (GE Healthcare) in buffer D (20 mM Tris pH 7.5, 150 mM NaCl, 5 mM β-mercaptoethanol). The same protocol was applied to purify CC2D1B (residues 317-558) and modified to purify Lgd (residues 358-816). After elution from the second Ni-NTA column, Lgd (residues 358-816) was dialyzed against buffer E (25 mM Na-citrate pH 6, 100 mM NaCl, 5 mM β-mercaptoethanol) and further purified on a Mono S column (GE Healthcare). The final purification step included gel filtration on a Superdex 200 column (GE Healthcare) in buffer E.

PIP Pull-Down Experiment

40-μl slurries of eight different phosphoinositide coated beads and control beads (Echelon. Inc) were washed once in 500 μl of binding buffer (10 mM HEPES pH 7.5, 300 mM NaCl, 1% (v/v) Nonidet P-40). After centrifugation, beads were incubated with 15 μg of purified Lgd (residues 358-816), rotating for 4 h at 4°C. Beads were pelleted by centrifugation and the supernatant was collected as the unbound fraction. The beads were washed five times in 500 μl of binding buffer and Lgd (residues 358-816) was eluted by

boiling the beads for 5 min in 20 μl of SDS–PAGE sample loading buffer. Fractions corresponding to unbound and bound proteins were analyzed by SDS-PAGE and bands were detected by Coomassie Blue Staining.

Sedimentation Experiments

The sucrose gradient centrifugation experiments were performed as described (Martinelli et al., 2012). Briefly the indicated proteins were separated on sucrose gradients in HBS buffer (0.01 M Hepes, pH 7.4, 0.15 M NaCl), by overlaying sucrose solutions of 60% (65 μl), 40%, 30%, 20% and 5% (85 μl each). Centrifugation was performed in a Beckman SW55 rotor at 40,000 rpm for 6h at 4°C. Fractions from the gradients were analysed on a 15% SDS-PAGE and bands were detected with Coomassie Blue staining.

Crystallization and Structure Solution of Lgd (550-816)

Lgd (residues 550-816) was concentrated to 5 mg/ml and crystals were obtained by the vapor diffusion method in hanging drops mixing equal volumes of complex and reservoir solution (0.1 M Bis-Tris pH 6.5, 15-25% PEG 3500, 200 mM ammonium sulfate). The crystal was cryo-cooled at 100 K in reservoir buffer containing 25% (v/v) glycerol. The selenomethionine substituted Lgd (residues 550-816) was crystallized under the same conditions. A complete native dataset and SAD data set were collected at the ESRF (Grenoble, France) beam line ID14-4. Data were processed and scaled with MOSFLM (Battye et al., 2011) and SCALA (Evans, 2006). The crystals belong to space group $P2_1$ with 2 molecules per asymmetric unit. Although the asymmetric unit contains two protomers, which dimerize via their helical N-terminal domains with a relatively large interface covering 964 $Å^2$, MALLS and analytical ultracentrifugation demonstrated a monomeric state of Lgd (550-816) in solution (data not shown). The unit cell dimensions are a=87.81 Å, b=54.22 Å, c=97.26 Å (β=98.19) for the native data set and a=87.08 Å, b=53.77 Å, c=97.26 Å (β=99.49) for the SAD data set. The structure was solved by the single anomalous dispersion method employing the data set collected at the peak wavelength (0.9795 Å) and the SAS protocol of Auto-Rickshaw (Panjikar et al., 2005) . Briefly, heavy atom sites were localized using the program SHELXD (Schneider and Sheldrick, 2002) and the correct hand for the substructure was determined using the programs ABS (Hao, 2004) and SHELXE (Sheldrick, 2002). Initial phases were calculated after density modification using the program SHELXE (Sheldrick, 2002) and they were improved by density modification and NCS averaging using the program DM (Cowtan et al., 2011). An initial partial model was built with ARP/wARP (Morris et al., 2004) and BUCCANEER (Cowtan, 2006) and refinement with REFMAC (Murshudov et al., 1997) using data to 2.9 Å resolution (R_{factor} of 0.29 and R_{free} of 0.34). The incomplete model was subsequently employed as search model for molecular replacement with Phaser (McCoy et al., 2007) and the 2.4 Å native data set. Model building was completed manually with Coot (Emsley et al., 2010) and the model was refined with REFMAC (Murshudov et al., 1997) and Phenix (Adams et al., 2010) to an R_{factor} of 0.2067 and R_{free} of 0.2459. The model contains chain A residues 575 to 815 and chain B residues 575 to 815. N-terminal residues 550 to 576 were disordered in both protomers and 89.79/7.92 % of the residues are within the most favored and allowed regions of a Ramachandran plot (CCP4, 1994). Molecular graphics figures were generated with PyMOL (W. Delano; http://www.pymol.org) and sequence alignments with the program ESPript (Gouet et al., 1999).

Modelling of the corresponding CC2D1B fragment was performed with the SWISS-MODEL protein structure homology-modelling server (Biasini et al., 2014). Docking of Ins(1,4,5)P3 to the CC2D1B structural model was performed with the SwissDock web server (Grosdidier et al., 2011).

QUANTIFICATION AND STATISTICAL ANALYSIS

All graphs and statistical test were performed using Prism (GraphPad Software). Statistical significance was tested using two-tailed unpaired t-test. All statistical information for quantitative datasets is shown in the corresponding figure legend.

DATA AND SOFTWARE AVAILABILITY

Co-ordinates and structure factures corresponding to Lgd (residues 550-816) have been deposited in the Protein Data Bank with accession ID 6EI6.

Developmental Cell, Volume *47*

Supplemental Information

CC2D1B Coordinates ESCRT-III Activity during the Mitotic Reformation of the Nuclear Envelope

Leandro N. Ventimiglia, Miguel Angel Cuesta-Geijo, Nicolas Martinelli, Anna Caballe, Pauline Macheboeuf, Nolwenn Miguet, Ian M. Parnham, Yolanda Olmos, Jeremy G. Carlton, Winfried Weissenhorn, and Juan Martin-Serrano

Figure S1

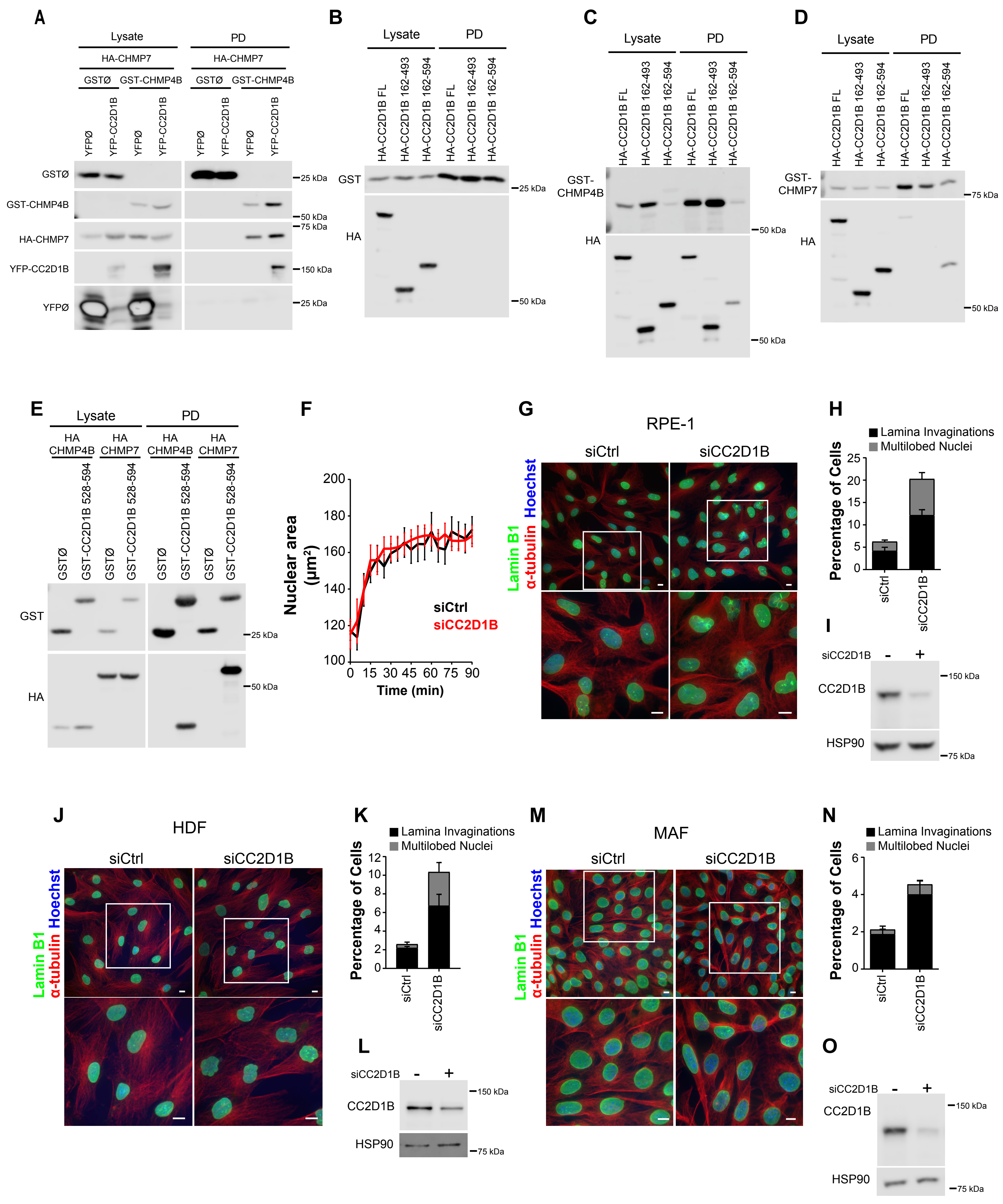

A
Lysate
PD
HA-CHMP7
GSTØ
GST-CHMP4B
YFPØ
YFP-CC2D1B
GSTØ
GST-CHMP4B
HA-CHMP7
YFP-CC2D1B
YFPØ
25 kDa
50 kDa
75 kDa
150 kDa
B
HA-CC2D1B FL
HA-CC2D1B 162-493
HA-CC2D1B 162-594
GST
HA
C
GST-CHMP4B
D
GST-CHMP7
E
HA CHMP4B
HA CHMP7
GST-CC2D1B 528-594
F
Nuclear area (µm²)
Time (min)
siCtrl
siCC2D1B
G
RPE-1
Lamin B1
α-tubulin
Hoechst
H
Lamina Invaginations
Multilobed Nuclei
Percentage of Cells
I
siCC2D1B
CC2D1B
HSP90
J
HDF
K
L
M
MAF
N
O

Figure S2

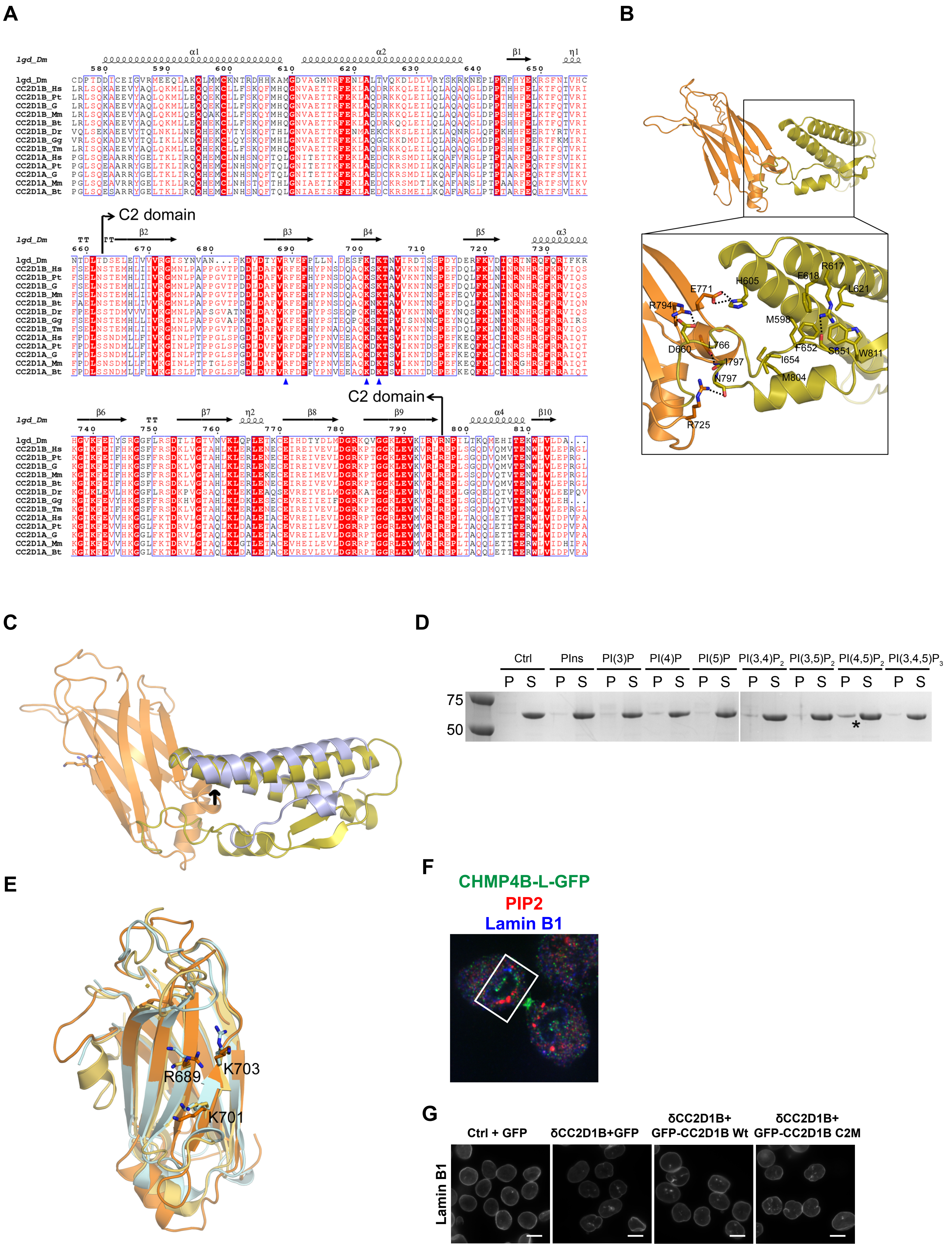
A
B
C
D
E
F
G
C2 domain
C2 domain
lgd_Dm
CC2D1B_Hs
CC2D1B_Pt
CC2D1B_G
CC2D1B_Mm
CC2D1B_Bt
CC2D1B_Dr
CC2D1B_Gg
CC2D1B_Tm
CC2D1A_Hs
CC2D1A_Pt
CC2D1A_G
CC2D1A_Mm
CC2D1A_Bt
E771
H605
R617
F618
L621
R794
M598
L766
D660
I797
F652
S651
W811
I654
N797
M804
R725
Ctrl
PIns
PI(3)P
PI(4)P
PI(5)P
PI(3,4)P2
PI(3,5)P2
PI(4,5)P2
PI(3,4,5)P3
P
S
75
50
*
R689
K703
K701
CHMP4B-L-GFP
PIP2
Lamin B1
Ctrl + GFP
δCC2D1B+GFP
δCC2D1B+ GFP-CC2D1B Wt
δCC2D1B+ GFP-CC2D1B C2M
Lamin B1

Figure S3

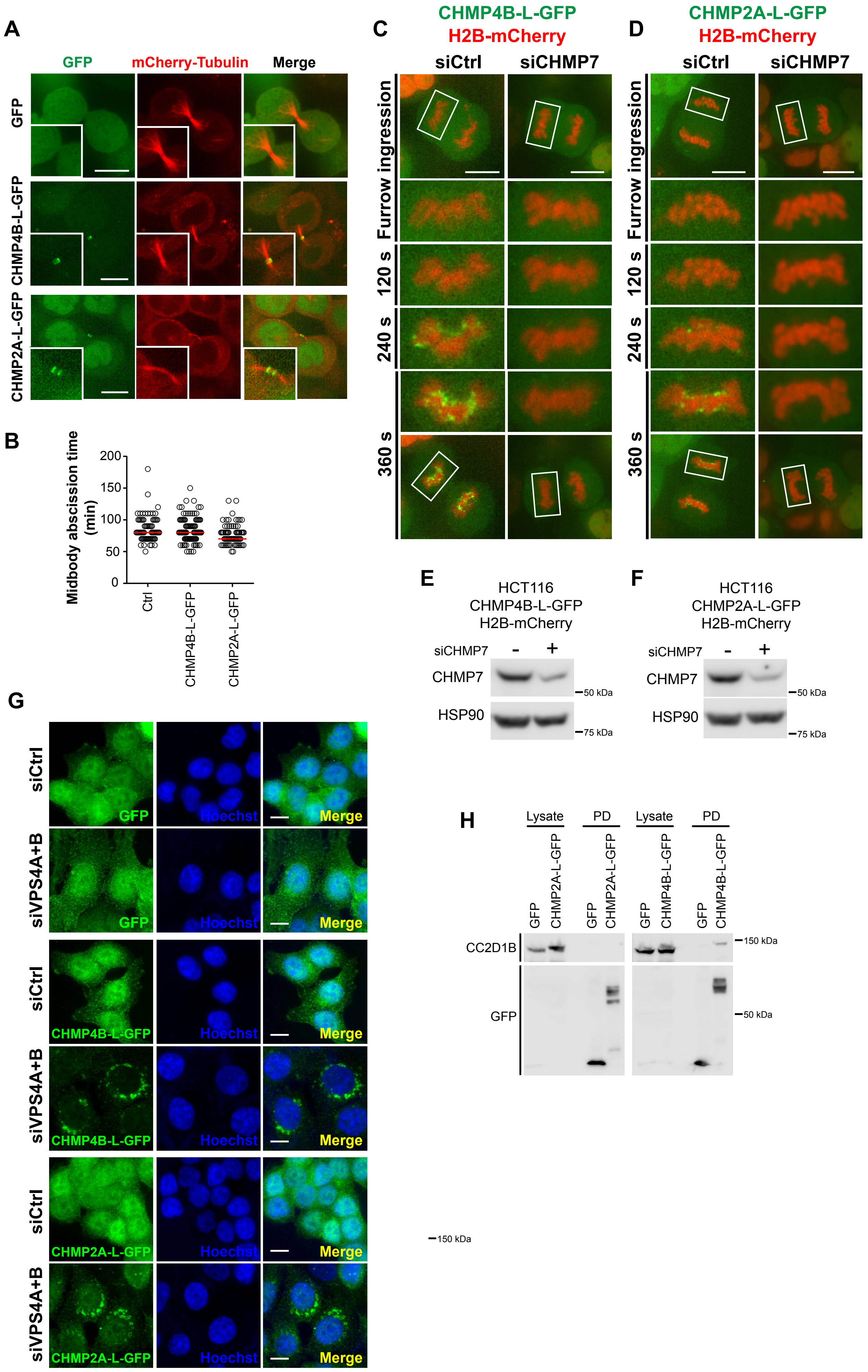
A
GFP
mCherry-Tubulin
Merge
GFP
CHMP4B-L-GFP
CHMP2A-L-GFP
B
Midbody abscission time (min)
200
150
100
50
0
Ctrl
CHMP4B-L-GFP
CHMP2A-L-GFP
C
CHMP4B-L-GFP
H2B-mCherry
siCtrl
siCHMP7
Furrow ingression
120 s
240 s
360 s
D
CHMP2A-L-GFP
H2B-mCherry
siCtrl
siCHMP7
Furrow ingression
120 s
240 s
360 s
E
HCT116
CHMP4B-L-GFP
H2B-mCherry
siCHMP7 - +
CHMP7
50 kDa
HSP90
75 kDa
F
HCT116
CHMP2A-L-GFP
H2B-mCherry
siCHMP7 - +
CHMP7
50 kDa
HSP90
75 kDa
G
siCtrl
siVPS4A+B
GFP
Hoechst
Merge
CHMP4B-L-GFP
CHMP2A-L-GFP
H
Lysate
PD
Lysate
PD
GFP
CHMP2A-L-GFP
GFP
CHMP2A-L-GFP
GFP
CHMP4B-L-GFP
GFP
CHMP4B-L-GFP
CC2D1B
GFP
150 kDa
50 kDa
150 kDa

Figure S4

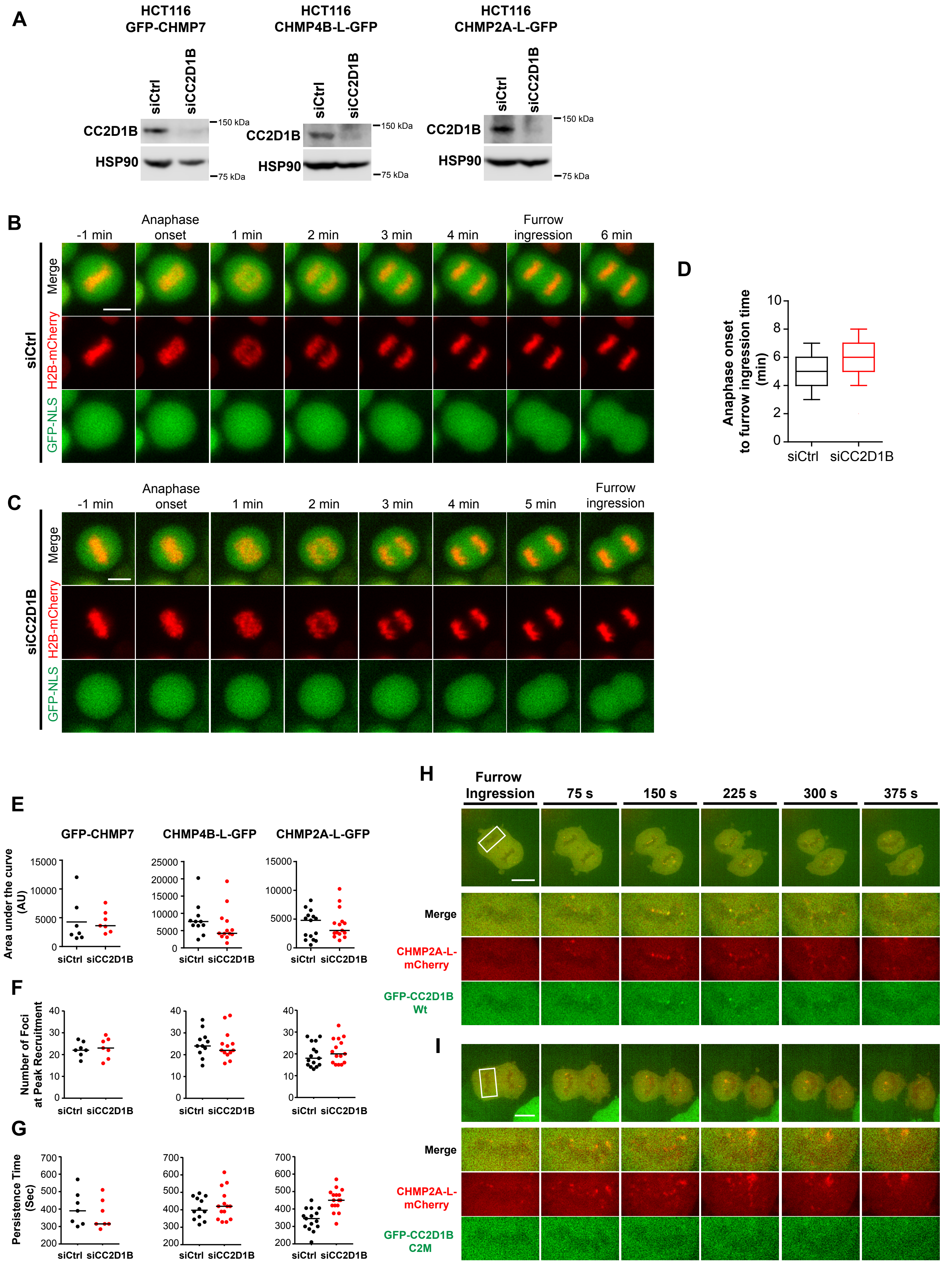
A
HCT116 GFP-CHMP7
HCT116 CHMP4B-L-GFP
HCT116 CHMP2A-L-GFP
siCtrl
siCC2D1B
CC2D1B
HSP90
150 kDa
75 kDa
B
-1 min
Anaphase onset
1 min
2 min
3 min
4 min
Furrow ingression
6 min
siCtrl
Merge
H2B-mCherry
GFP-NLS
C
5 min
siCC2D1B
D
Anaphase onset to furrow ingression time (min)
E
GFP-CHMP7
CHMP4B-L-GFP
CHMP2A-L-GFP
Area under the curve (AU)
F
Number of Foci at Peak Recruitment
G
Persistence Time (Sec)
H
Furrow Ingression
75 s
150 s
225 s
300 s
375 s
CHMP2A-L-mCherry
GFP-CC2D1B Wt
I
GFP-CC2D1B C2M

Figure S5

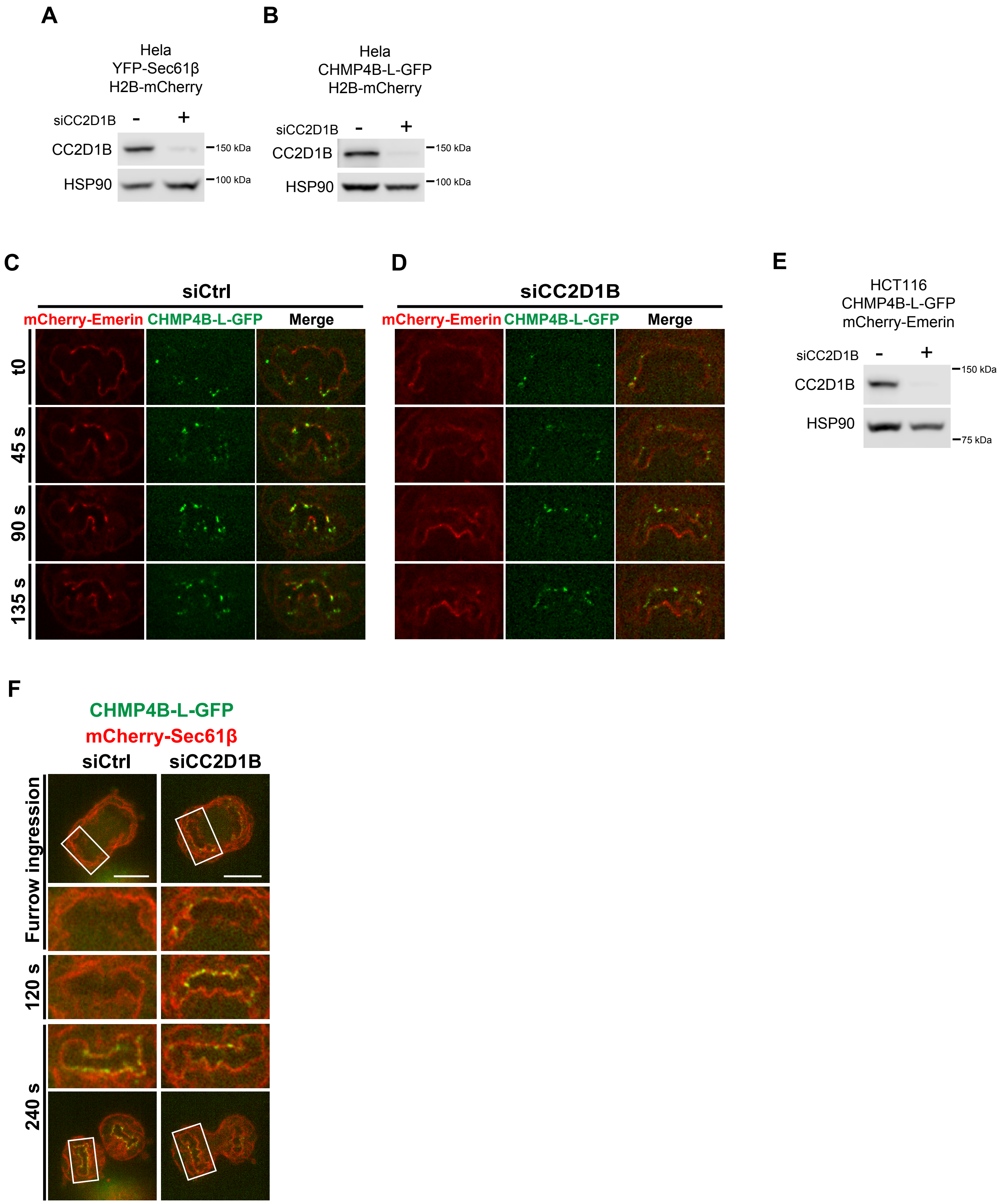
A
Hela
YFP-Sec61β
H2B-mCherry
siCC2D1B - +
CC2D1B
HSP90
150 kDa
100 kDa
B
Hela
CHMP4B-L-GFP
H2B-mCherry
siCC2D1B - +
CC2D1B
HSP90
150 kDa
100 kDa
C
siCtrl
mCherry-Emerin
CHMP4B-L-GFP
Merge
t0
45 s
90 s
135 s
D
siCC2D1B
mCherry-Emerin
CHMP4B-L-GFP
Merge
E
HCT116
CHMP4B-L-GFP
mCherry-Emerin
siCC2D1B - +
CC2D1B
HSP90
150 kDa
75 kDa
F
CHMP4B-L-GFP
mCherry-Sec61β
siCtrl
siCC2D1B
Furrow ingression
120 s
240 s

Figure S6

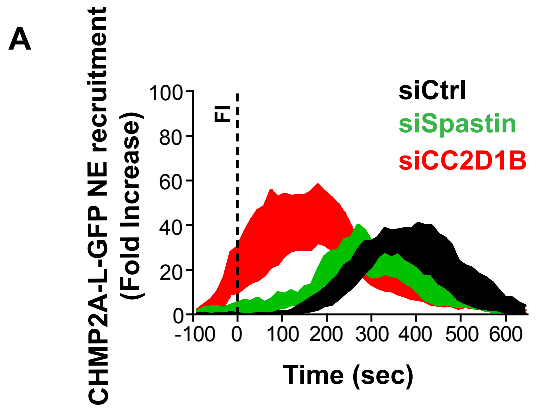
A
CHMP2A-L-GFP NE recruitment
(Fold Increase)
100
80
60
40
20
0
-100
0
100
200
300
400
500
600
Time (sec)
FI
siCtrl
siSpastin
siCC2D1B

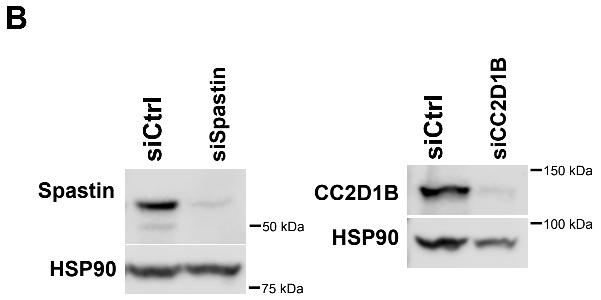
B
siCtrl
siSpastin
Spastin
HSP90
50 kDa
75 kDa
siCtrl
siCC2D1B
CC2D1B
HSP90
150 kDa
100 kDa

Figure S7

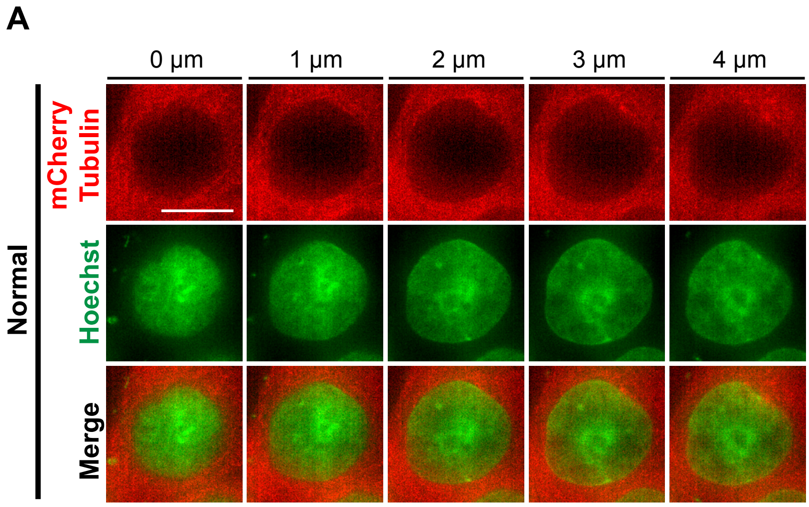

Table S1. Related to Figure 3. Data collection and refinement statistics

	LgD SAD	LgD native (PDB code)
**Data collection**		
Space group	P1 21 1	P1 21 1
Cell dimensions		
a, *b*, *c* (Å)	87.35, 53.59, 97.85	87.81, 54.22, 98.19
α, β, γ (°)	90, 99.49, 90	90, 99.49, 90
Wavelength (Å)	0.9795	0.9750
Resolution (Å)	96.5 2.9 (3.06-2.9)[a]	9-.55-2.46 (2.59-2.46)
R_{merge}	8.5 (34.5)	7.2 (36.3)
$I/\sigma(I)$	10.1 (3.8)	12.0 (3.6)
$CC_{1/2}$	99.3 (89.2)	99.5 (88.0)
Completeness (%)	99.4 (99.5)	99.4 (99.2)
Redundancy	3.9 (3.9)	3.5 (3.5)
**Refinement**		
Resolution (Å)		59.59-2.46 (2.53-2.46)
No. reflections		32921 (3242)
R_{work} / R_{free}		0.2067/0.2459 (0.2846/0.3533)
No. atoms		4158
Protein		3952
Water		164
Ligands		42
B factors		
Protein		47.96
Water		44.78
Ligands		77.20
R.m.s. deviations		
Bond lengths (Å)		0.010
Bond angles (°)		1.23

[a] Values in parentheses are for highest-resolution shell.

Supplemental information

Figure S1. Related to Figure 1.

(A) GST pulldown experiments of 293T cells transiently co-expressing GST-CHMP4B, HA-CHMP7 and YFP-CC2D1B.

(B) GST pulldown experiments of 293T cells transiently co-expressing GST together with HA-CC2D1B full length, HA-CC2D1B (residues 162-493) or HA-CC2D1B (residues 162-594).

(C) GST pulldown experiments of 293T cells transiently co-expressing GST-CHMP4B together with HA-CC2D1B full length, HA-CC2D1B (residues 162-493) or HA-CC2D1B (residues 162-594).

(D) GST pulldown experiments of 293T cells transiently co-expressing GST-CHMP7 together with HA-CC2D1B full length, HA-CC2D1B (residues 162-493) or HA-CC2D1B (residues 162-594).

(E) GST pulldown experiments of 293T cells transiently co-expressing GST-CC2D1B (residues 528-594) together with HA-CHMP7 or HA-CHMP4B.

(F) Representation the nuclear expansion rate in HCT116 cells stably co-expressing GFP-NLS and H2B-mCherry. Mean±SEM; siControl n=15; siCC2D1B n=21, p=0.5941. Significance compared with the control was calculated at 90 minutes using a two-tailed unpaired t-test.

(G-I) Analysis of nuclear morphology in siRNA transfected RPE-1 cells. (G) Representative images corresponding to the quantifications shown in H. Scale bars 10µm. (H) Percentage of cells showing an aberrant nuclear morphology. Mean±SEM; siControl n=1192; siCC2D1B n=924, p=0.0057. Significance of NE invaginations compared to the control was calculated using a two-tailed unpaired t-test. (I) Representative WB corresponding to cells shown in G.

(J-L) Analysis of nuclear morphology in siRNA transfected HDF cells. (J) Representative images corresponding to the quantifications shown in K. Scale bars 10µm. (K) Percentage of cells showing an aberrant nuclear morphology. Mean±SEM; siControl n=731; siCC2D1B n=736, p=0.0216. Significance of NE

invaginations compared to the control was calculated using a two-tailed unpaired t-test. (L) Representative WB corresponding to cells shown in J.

(M-O) Analysis of nuclear morphology in siRNA transfected MEF cells. (M) Representative images corresponding to the quantifications shown in N. Scale bars 10µm. (N) Percentage of cells showing an aberrant nuclear morphology. Mean±SEM; siControl n=1589; siCC2D1B n=1523, p=0.0387. Significance of NE invaginations compared to the control was calculated using a two-tailed unpaired t-test. (O) Representative WB corresponding to cells shown in M.

Figure S2. Related to Figure 3.

(A) Sequence alignment of the C-terminal domains of the Lgd/CC2D1 family of proteins. Secondary structure elements are shown based on the Lgd (residues 575-816) crystal structure. The sequence corresponding to the C2 domain is indicated as well as the conserved residues that may coordinate PIns(4,5)P_2 (blue triangles). The following sequences are shown: accession code AAF53069 lgd drosophila melanogaster (Dm) isoform A; XP_005270647 Homo sapiens (Hs) 1B isoform X1; JAA36276 CC2D1B Pan troglodytes (Pt); XP_004025849 gorilla gorilla (G) 1B; XP_006503215 mus musculus (Mm) 1B isoform x1; XP_002686449 Bos taurus (Bt) 1B ; XP_005171255 Danio rerio (Dr) 1B X1 ; XP_001235393 Gallus gallus (Gg) 1B ; XP_004371788 CC2D1B Trichechus manatus latirostris (Tm); XP_005260029 Homo sapiens (Hs) 1A isoform X1; XP_524131 Pan troglodytes (Pt) 1A; XP_004060195 gorilla gorilla (G) 1A; XP_006530873 mus musculus (Mm)1A isoform X1; XP_005208662 Bos taurus (Bt) 1A isoform x1.

(B) Close up of the interactions stabilizing the helical domain and its orientation towards the C2 domain; the helical hairpin interacts via a hydrophobic core and hydrogen bonds with the coil region connecting to the C2 domain and its orientation is stabilized by salt bridges and hydrogen bonds producing a rigid structure. Polar interactions are indicated by dashed lines.

(C) Cα superposition of the Lgd helical hairpin and the DM14-3 domain (pdb code 5vo5) shows high structural similarity of the C-terminal helical domain

(yellow) and the DM14-3 domain (light blue). Shrub/CHMP4 binds perpendicular to the tip of the helical hairpin of DM14-3 (indicated by the arrow).

(D) Analysis of Lgd (residues 358-816) binding to phosphatidylinositol by pull-down; Ctrl, control beads; P, pellet; S, supernatant. A significant pull down is observed with PIns(4,5)P_2 (*).

(E) Ribbon diagram of the superpositioning of the Cα atoms of Lgd (orange) and the C2 domains from protein kinase C (yellow, pdb entry 3GPE) and synaptotagmin C2A (polycyan, pdb entry 1RSY); the residues of the basic cluster implicated in PIns(4,5)P_2 binding are indicated and correspond to CC2D1B residues R721, K734, K736.

(F) HCT116 dividing cell stably expressing CHMP4B-L-GFP and stained with anti-GFP, -PIP2 and Lamin B1 antibodies. The nuclear inset is shown in Figure 3G.

(G) Representative images of the HCT116 cells used for quantification shown in Fig 3I. Scale bars 10µm.

Figure S3. Related to Figure 4.

Functional characterization of HCT116 cells stably expressing CHMP4B-L-GFP or CHMP2A-L-GFP.

(A) Representative images of dividing living cells stably co-expressing CHMP4B-L-GFP or CHMP2A-L-GFP along with mCherry-Tubulin. Scale bars 10µm.

(B) Midbody abscission time in cells stably co-expressing CHMP4B-L-GFP or CHMP2A-L-GFP along with mCherry-Tubulin. Bars indicate Median. Control n=74; CHMP4B-L-GFP n=97, p=0.5748; CHMP2A-L-GFP n=75, p=0.0015. Significance compared to the control was calculated using a two-tailed unpaired t-test.

(C-F) Time-lapse analysis of HCT116 cells stably co-expressing CHMP4B-L-GFP (C) or CHMP2A-L-GFP (D) along with H2B-mCherry. Cells were transfected with control or CHMP7 siRNAs. T0 was set at the beginning of furrow ingression. (E-F) Representative WB corresponding to cells shown in C and D. Scale bars 10µm in C and D.

(G) Representative images of HCT116 cells stably expressing CHMP4B-L-GFP or CHMP2A-L-GFP transfected with control or VPS4A and VPS4B siRNAs. Cells were stained with an anti-GFP antibody. Scale bars 10µm.

(H) Representative WB corresponding to a GFP immunoprecipitation experiment of HCT116 cells stably expressing CHMP4B-L-GFP or CHMP2A-L-GFP.

See also Movies 6 and 7.

Figure S4. Related to Figure 4.

(A) Representative WB corresponding to cells shown in Fig 4 A-C.

(B-D) Anaphase onset to furrow ingression time quantification of HCT116 cells stably co-expressing GFP-NLS with H2B-mCherry. Representative time lapse images corresponding to cells transfected with control (B) or CC2D1B (C) siRNAs. (D) Anaphase onset to furrow ingression time quantification. Bar indicates Median. siCtrl n = 348; siCC2D1B n = 362, $p<0.0001$. Significance compared to the control was calculated using a two-tailed unpaired t-test. Scale bars 10µm in B and C.

(E) Area under the curves corresponding to the total amount of GFP-CHMP7, CHMP4B-L-GFP or CHMP2A-L-GFP fluorescence recruited to the reforming NE. Cells were transfected with control or CC2D1B siRNA. Bar indicates Median. GFP-CHMP7 $p=0.9836$; CHMP4B-L-GFP $p=0.3858$; CHMP2A-L-GFP $p=0.8959$. Significance compared to the control was calculated using a two-tailed unpaired t-test.

(F) Number of GFP-CHMP7, CHMP4B-L-GFP or CHMP2A-L-GFP foci on the NE at peak recruitment. Cells were transfected with control or CC2D1B siRNA. Bar indicates Median. GFP-CHMP7 $p=0.8006$; CHMP4B-L-GFP $p=0.8180$; CHMP2A-L-GFP $p=0.3793$. Significance compared to the control was calculated using a two-tailed unpaired t-test.

(G) Persistence time of GFP-CHMP7, CHMP4B-L-GFP or CHMP2A-L-GFP fluorescence on the NE. Cells were transfected with control or CC2D1B siRNA.

Bar indicates Median. GFP-CHMP7 p=0.4986; CHMP4B-L-GFP p=0.5039; CHMP2A-L-GFP p=<0.0001. Significance compared to the control was calculated using a two-tailed unpaired t-test.

(H-I) Representative time lapse analysis corresponding to HCT116$^{\delta CC2D1B}$ cells co-expressing CHMP2A-L-mCherry and GFP-CC2D1B Wt (H) or C2M (I). Scale bars 10µm.

See also Movie 11 and 12.

Figure S5. Related to Figure 5.

(A-B) Representative WB corresponding to cells shown in Figures 5A and 5B.

(C-E) Super resolution time-lapse analysis of HCT116 cells stably co-expressing CHMP4B-L-GFP and mCherry-Emerin corresponding to the images shown in Figure 5D. Cells were transfected with control (C) or CC2D1B (D) siRNAs. (E) Representative WB corresponding to cells shown in Figure 5D and Figure S5C-S5D.

(F) Time-lapse analysis of HCT116 cells stably co-expressing CHMP4B-L-GFP and mCherry-Sec61β. Cells were transfected with control or CC2D1B siRNAs. Scale bars 10µm.

See also Movies 13.

Figure S6. Related to Figure 6.

(A) Quantification of CHMP2A-L-GFP recruitment to the reforming NE in HCT116 cells stably co-expressing CHMP2A-L-GFP with mCherry-Tubulin and transfected with control, Spastin or CC2D1B siRNAs. T0 was set at the beginning of furrow ingression (FI).

(B) Representative WB corresponding to cells shown in Figure 6E**.**

Figure S7. Related to Figure 7.

(A) Z-stack series corresponding to a HCT116 cell stably co-expressing mCherry-Tubulin with CHMP2A-L-GFP, transfected with control siRNA and incubated with Hoechst 33258 30’ before imaging to stain DNA. The signal corresponding to Hoechst 33258 is shown in green. The signal corresponding to CHMP2A-L-GFP is not shown. This cell was selected as an example of a nucleus with normal rounded morphology. Scale bar 10µm.

See also Movie 16.

Table S1. Related to Figure 3.

Data collection and refinement statistics of Lgd (residues 575-816) solved structure.
